# Zr(IV) Catalyst for the Ring-Opening Copolymerization of Anhydrides (A) with Epoxides (B), Oxetane (B), and Tetrahydrofurans (C) to Make ABB- and/or ABC-Poly(ester-*alt*-ethers)

**DOI:** 10.1021/jacs.2c01225

**Published:** 2022-04-07

**Authors:** Ryan W. F. Kerr, Charlotte K. Williams

**Affiliations:** Chemistry Research Laboratory, University of Oxford, Oxford OX1 3TA, U.K.

## Abstract

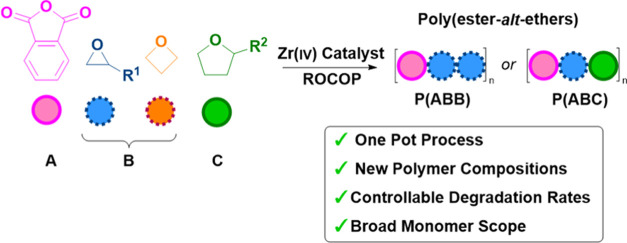

Poly(ester-*alt*-ethers) can combine beneficial ether linkage flexibility and polarity with ester linkage hydrolysability, furnishing fully degradable polymers. Despite their promising properties, this class of polymers remains underexplored, in part due to difficulties in polymer synthesis. Here, a catalyzed copolymerization using commercially available monomers, butylene oxide (BO)/oxetane (OX), tetrahydrofuran (THF), and phthalic anhydride (PA), accesses a series of well-defined poly(ester-*alt*-ethers). A Zr(IV) catalyst is reported that yields polymer repeat units comprising a ring-opened PA (**A**), followed by two ring-opened cyclic ethers (**B/C**) (−**ABB**– or −**ABC**−). It operates with high polymerization control, good rate, and successfully enchains epoxides, oxetane, and/or tetrahydrofurans, providing a straightforward means to moderate the distance between ester linkages. Kinetic analysis of PA/BO copolymerization, with/without THF, reveals an overall second-order rate law: first order in both catalyst and butylene oxide concentrations but zero order in phthalic anhydride and, where it is present, zero order in THF. Poly(ester-*alt*-ethers) have lower glass-transition temperatures (−16 °C < *T*_g_ < 12 °C) than the analogous alternating polyesters, consistent with the greater backbone flexibility. They also show faster ester hydrolysis rates compared with the analogous **AB** polymers. The Zr(IV) catalyst furnishes poly(ester-*alt*-ethers) from a range of commercially available epoxides and anhydride; it presents a straightforward method to moderate degradable polymers’ properties.

## Introduction

Polyesters are important in packaging, consumer products, clothing, and medicine.^1,2^ Many are sustainable polymers with monomers that are/could be bio-based or waste-sourced and with structures amenable to controlled hydrolysis providing routes to chemical recycling and, in some cases, to biodegradation.^3−5^ Nonetheless, their properties and scalable synthesis still require research;^6^ this work concerns a well-controlled, catalyzed polymerization yielding amorphous, flexible polyesters, showing low glass-transition temperatures. These materials could be useful, in future, as elastomers, polyester polyols, additives, and surfactants.

Poly(ester-*alt*-ether) structures combine the flexibility typical of ether linkages with the degradability of ester moieties. For example, the ring-opening polymerization (ROP) of 1,4-dioxan-2-one (PDX) yields poly(ester-*alt*-ether) with perfectly alternating glycolic acid and ethylene oxide repeat units.^7,8^ PPDX shows useful thermal properties (*T*_g_ = −13 °C, *T*_m_ = 106 °C) and a tensile strength of >48 MPa with high elongation at break >500%; it is tougher than polylactide (PLA) or polyethylene. Nonetheless, its use appears somewhat limited by its high temperature instability caused by thermodynamics, which favor PDX, hampering polymer processing.^9^ The ROP of 3-methyl-1,4-dioxan-2-one (MDO) affords poly(ester-*alt*-ether) with alternating lactic acid and ethylene oxide repeat units. It is an amorphous polymer, which can be blended into commercial PLA, showing potential as a plasticizer.^10^ Once again, the polymerization thermodynamics favor depolymerization at higher temperatures and the monomer synthesis has a low overall yield (20%). The alternating ROP of epoxides and low-ring strain lactones, e.g., dihydrocoumarin, can also form poly(ester-*alt*-ether).^11,12^ The polymerization is feasible because the low lactone polymerization free energy precludes lactone ROP and drives the reaction with the high free-energy epoxides.^11,13,14^

The ring-opening copolymerization (ROCOP) of anhydrides (**A**) and epoxides (**B**) is a controlled polymerization route to polyesters showing alternating **AB** sequences.^4,15−17^ Both monomers have attractions including a large number being existing commercial products and many are accessible from bio-based raw materials and wastes.^18−21^ The polymerization thermodynamics are driven toward polymers, allowing for the production of aliphatic, semiaromatic, and functionalized polyesters. Almost all ROCOP catalysts yield highly alternating polyesters.^4,15−17^ Some catalysts form uncontrolled ether linkages but such structures are inherently irregular. A few catalysts also allow for epoxide ROP, but this occurs after anhydride consumption, forming poly(ester-*b*-ethers).^15,22,23^

The alternating polyesters, prepared by ROCOP, typically feature C2 linkers between ester moieties. There are indications that such closely spaced ester groups slow hydrolysis and polymer degradation.^24^ The ester linkage separation could be increased if alternating polyesters of anhydrides with oxetane (C3)^25−27^ or tetrahydrofuran (THF) (C4)^28^ could be prepared, but such catalyzed polymerizations are underexplored. A pioneering report of oxetane (OX)/anhydride ROCOP by Endo and co-workers describes a Ti(IV)bisphenolate complex that shows a turnover frequency (TOF) of ∼3 h^–1^ ([Cat]/[OX]/[anhydride], 1:60:60, 120 °C, DCM).^25^ Another report describes a cationic bistriflimidic acid catalyst for THF/anhydride ROCOP, but it requires a high catalyst loading and has a low TOF (3–10 h^–1^, [Cat]/[THF]/[anhydride] 1:20:20, 130 °C).^28^ There is just one report, from 1973, of a tri(isobutyl)Al(III)/water catalyst system, affording, under specific conditions, **ABC** poly(ester-*alt*-ether) structures from anhydrides (**A**), epoxides (**B**), and THF (**C**).^29^ Curiously, the **ABC** sequences only formed for reactions in excess THF, and without it, alternating **AB** polyesters form. In 2021, Phomphrai and co-workers reported an Sn(II) catalyst for anhydride and cyclohexene oxide (CHO) ROCOP, which produced polymers with mixtures of **ABB** (70%) and **AB** + **BBB** linkages.^30^ DFT calculations suggested that the unusual selectivity might arise from steric hindrance and the Sn(II) lone pair. Last year, we reported that the commercially used Sn(Oct)_2_/alcohol catalyst system exposed to epoxides/anhydrides yields polyesters with random ether linkages, i.e., poly(PO-*ran*-MA) (PO/MA = ∼3:1).^31^ Combining the new polymers with l-lactide resulted in polymers showing potential as PLLA plasticizers.

**Scheme 1:**
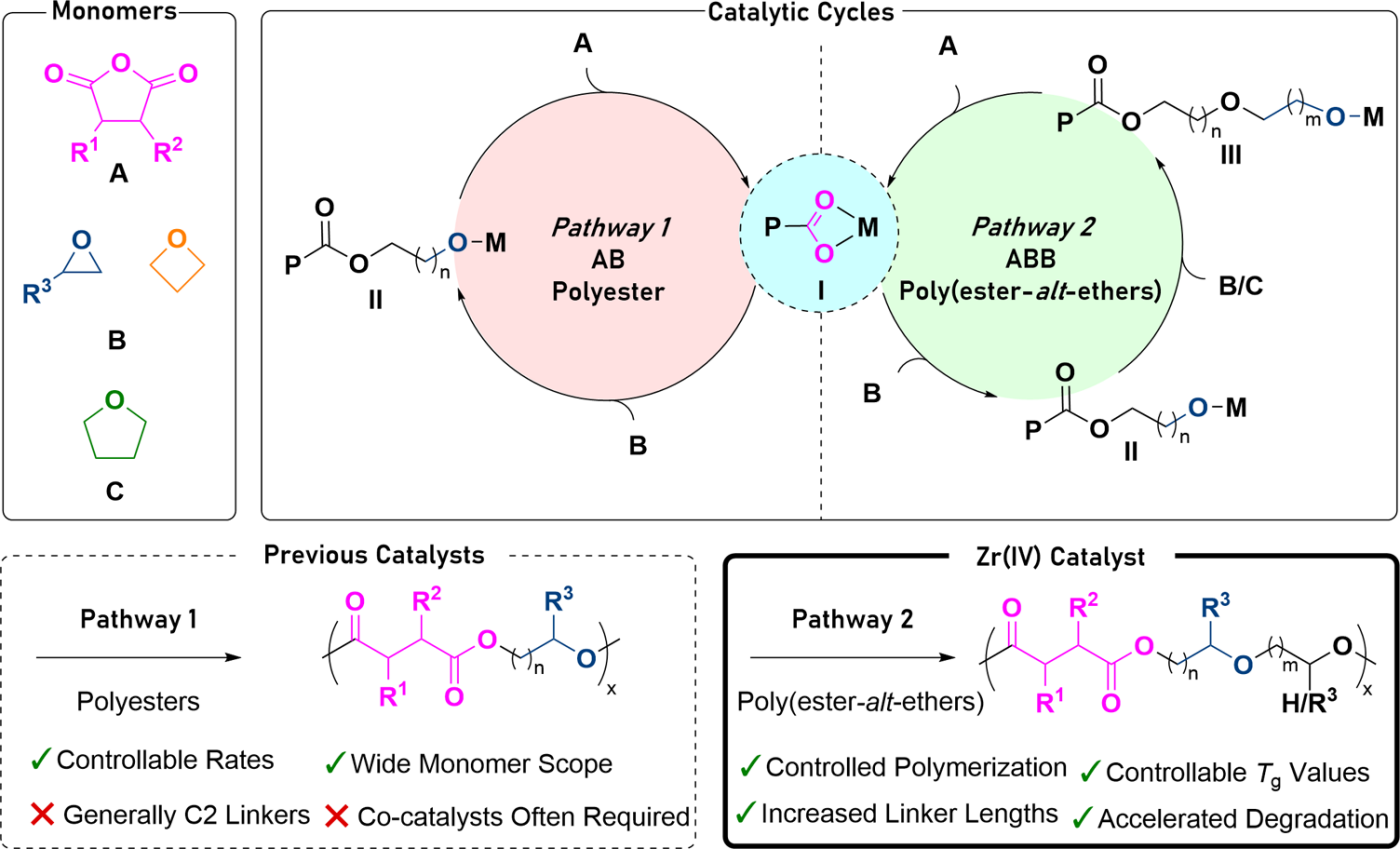
Top Left: Monomers Used Within This Work, Top Right: Catalytic Cycles for Anhydride/Cyclic Ether Ring-Opening Copolymerization (ROCOP), Bottom Left: **AB** Polyester Formed by Most ROCOP Catalysts, and Bottom Right: **ABB** or **ABC** Poly(ester-*alt*-ethers) Formed by the Zr(IV) Catalyst P: polymeryl chain; M: metal active site; R_1_ = R_2_ = phenylene, R_3_ = Me, Et.

Here, a new Zr(IV) catalyst is reported for phthalic anhydride (**A**) and epoxide/oxetane (**B**) ROCOP, which yields polymers with **ABB** sequences. Usually, anhydride/epoxide ROCOP catalysts show high selectivity for **AB** sequences.^16^ To understand the unusual Zr(IV) catalyst selectivity, the generalized ROCOP catalytic cycle should be considered. During polymerization, an alkoxide initiator reacts with anhydride (**A**) to form a carboxylate intermediate **I**, which reacts with an epoxide (**B**) to form an alkoxide species **II**. Repetition of the sequential monomer insertion cycles builds up the **AB** polymers (Scheme 1, pathway 1).^32^ We hypothesized that to construct the **ABB**/**ABC** repeat unit sequences observed in this work, the catalytic cycle should be identical until the formation of the alkoxide intermediate **II**, which then must preferentially ring-open another cyclic ether (**B** or **C**). Such a ring opening would furnish a second alkoxide intermediate **III** (Scheme 1, pathway 2). Next, the alkoxide intermediate **III** must ring-open an anhydride (**A**) to regenerate the carboxylate intermediate (**I**) (Scheme 1, pathway 2). The precedent for such selectivity is very limited, presumably because any successful system must overcome rapid anhydride insertion kinetics (rate laws are usually zero order in anhydride concentration).^16^ Most ROCOP catalysts feature first-row s-block, transition metals, or main-group Lewis acids. We hypothesized that larger ionic radii metals might reduce active site steric hindrance, potentially enabling *cis*-mononuclear mechanisms^33^ and obviating the use of co-catalysts, and could accelerate rates.

Epoxide/anhydride ROCOP catalysts must balance sufficient Lewis acidity to coordinate the monomers, with the right metal-alkoxide/carboxylate lability to accelerate monomer insertions.^34,35^ Group 4 metal complexes meet these criteria and often show low toxicity, cost, redox stability, and lack of color and they are surprisingly little explored in this field of catalysis.^25,36^ Following the hypothesis that larger ionic radii metal active sites could be beneficial, we targeted Zr(IV) complexes. So far, there is only one Zr(IV) catalyst for epoxide/anhydride ROCOP, coordinated by a benzoxazole ligand, which turned over slowly and produced perfectly alternating AB polyesters.^36^ Group 4 phenoxy-imine complexes are an important class of olefin polymerization precatalysts, and they can feature alkyl, halide, or imido co-ligands. These olefin polymerization catalysts generally enchain by a cationic active site after activation.^37−43^ We envisioned that related complexes, featuring alkoxide co-ligands and a neutral active site, might be effective ring-opening polymerization catalysts.

## Results and Discussion

### Zr(IV) Catalyst 1

**Figure 1:**
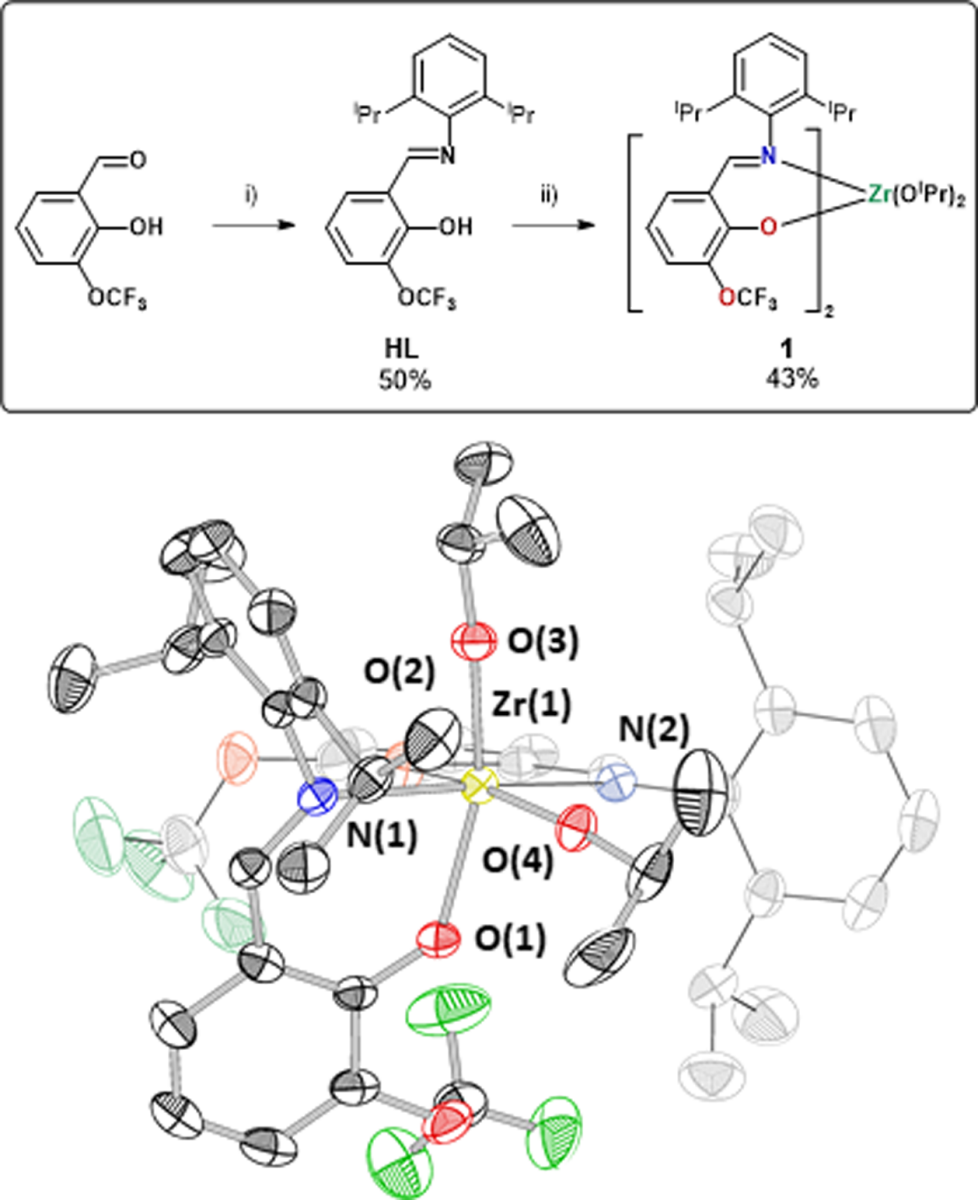
Top: Synthesis of Zr(IV) catalyst **1**. Reagents and conditions: (i) 2,6-(CH(CH_3_)_2_)_2_C_6_H_3_NH_2_ (1 equiv), EtOH, 80 °C, 18 h and (ii) [Zr(O*^i^*Pr)_4_(HO*^i^*Pr)], toluene, 40 °C, 24 h. Bottom: Molecular structure of **1** from single-crystal X-ray diffraction; thermal ellipsoids presented at 50% probability and H-atoms omitted for clarity (atom color scheme: Zr (yellow), O (red), N (blue), C (grayscale/black), and F (green)).

Previously, a series of Schiff base ligands, derived from ortho-vanillin, were used to make polymerization catalysts (HL^Me^, see the Supporting Information).^34,44−47^ Ti(IV) complexes, coordinated by two ligands and two isopropoxide groups, show coordination-isomer-dependent activities for lactone ROP.^45^ First, we tested these Ti(IV) complexes, i.e., [Ti(IV)(*o*-vanillin schiff base)_2_(OR)_2_] in epoxide/anhydride ROCOP but they were inactive. Next, a Zr(IV) complex, coordinated by the Schiff base ligand, featuring 2,6-diisopropylphenyl substituents (HL^Me^), was targeted. The synthetic route used to make the Ti(IV) complexes was initially explored using Zr(IV) precursors but was unsuccessful (see the Supporting Information, Section S1.1).^45^ For example, [Zr(O*^i^*Pr)_4_(HO*^i^*Pr)] was treated with 2 equiv of proligand HL^Me^, in toluene, at −30 °C and allowed to warm to room temperature overnight. Using various different ortho-vanillin ligands, either no reaction took place or an undesired homoleptic complex Zr(L^Me^)_4_ was formed. The homoleptic complex was proposed to be stabilized by the large ionic radius Zr(IV), which has increased oxophilicity (ionic radii of six-coordinate Ti^4+^ = 0.61 Å and Zr^4+^ = 0.72 Å and the bond dissociation energies of Ti–O vs Zr–O = 662 kJ mol^–1^ vs 760 kJ mol^–1^).^48^ The ligand was modified by replacing the ortho-methoxy ether group with −OCF_3_ to reduce its donor ability and increase its steric bulk. The new proligand, HL_1_, was synthesized by adapting the reported ligand protocols (see the Supporting Information). A new heteroleptic Zr(IV) complex, **1**, was synthesized by the reaction of 2 equiv of **HL**_**1**_ with [Zr(O*^i^*Pr)_4_(HO*^i^*Pr)], in toluene (90% conversion, 40 °C, 18 h), and was isolated in 43% yield after recrystallization in hexane/THF (Figure 1). Control over the reaction temperature was critical: no reaction occurred at 0 °C and undesired tris- and tetrakis complexes formed at temperatures above 80 °C. The desired bis(alkoxide) complex **1** was characterized by NMR spectroscopy, elemental analysis, and X-ray crystallography. ^1^H and ^13^C NMR spectra show a single set of ligand resonances, indicative of *C*_2_-symmetric solution structures.^19^F NMR spectroscopy was also consistent with a single resonance for the ether group. Single-crystal X-ray diffraction of **1** reveals a solid-state structure with *C*_2_ symmetry. The Zr(IV) center adopts a distorted octahedral conformation (Figure 1). The ligands coordinate to it with the nitrogen atoms adopting *trans* conformations (N–Zr–N = 166.1°) and the isopropoxide ligands occupying mutually *cis* coordination sites (O–Zr–O = 98.5°). The *cis*-alkoxide coordination is emphasized since these are the sites occupied by the growing polymer(s).^41^ We also note that the active site structures closely resemble those of group 4 (Zr or Ti) phenoxy-imine complexes, which are very successful in olefin polymerizations. The geometry of **1** is quite different from previously reported Ti(IV) complexes, which show coordination isomerism dependent upon the imine substituents (N–O:N–O vs N–O:O–O chelates).^45^

### Anhydride and Epoxide Ring-Opening Copolymerization Catalysis

**Figure 2:**
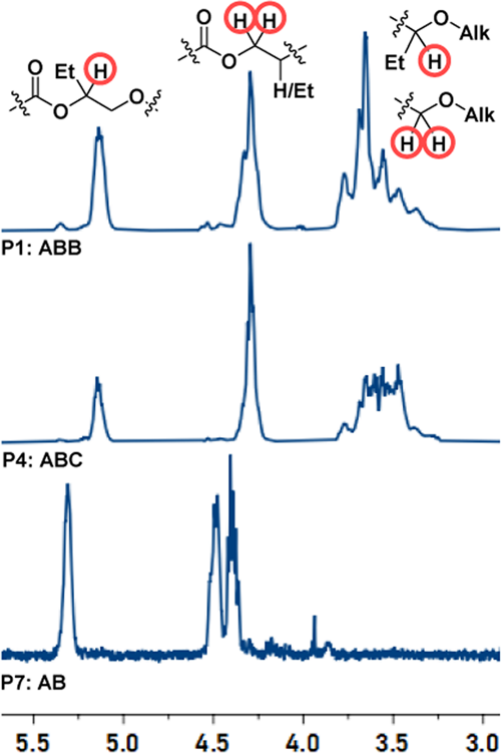
Selected regions of the ^1^H NMR spectra (400 MHz, CDCl_3_ referenced at 7.26 ppm), illustrating the methine and methylene resonances for **P1** (**ABB**), **P4** (**ABC**), and **P7** (**AB**), Alk = −CH_2_– or −CH(Et)–. Full spectra are available in Figures S9, S13, and S17.

Zirconium(IV) catalyst **1** was tested using phthalic anhydride (PA) and butylene oxide (BO) (Table 1, #1). The polymerization proceeded over 5 h to yield a polymer that incorporated ∼2 equiv of epoxide for every anhydride, as determined by ^1^H NMR spectroscopy (Figures 2 and S9). Notably, the diagnostic methine-ester (−COOC*H*(CH_2_CH_3_)CH_2_−) and methylene-ester (−COOC*H*_2_CH−) resonances of **P1** (δ_H_ = 5.14 and 4.30 ppm, respectively) are shifted upfield compared to those for the comparative alternating (AB) polymer, **P7** (δ_H_ = 5.31 and 4.43 ppm). The distinctive ester group chemical shifts suggest a different linkage sequence in **P1** (Figure S17). In addition, **P1** shows a new broad resonance at lower chemical shift, assigned to methine-ether (−OC*H*(CH_2_CH_3_)CH_2_−) and methylene-ether (−OC*H*_2_CH−) linkages (δ_H_ = 3.18–3.88 ppm). To confirm that the new ether linkages were contained within the same polymer (and not mixtures of products), ^1^H COSY NMR spectroscopy shows correlations between the ester and ether regions, but consistent with the proposed linkage structure, there were no correlations between the methine- and methylene-ester resonances (Figures S11 and S12). In contrast, **P7** shows correlations between all ester resonances (Figure S17). The ^13^C NMR spectrum for **P1** shows several carbonyl peaks ranging from 167.8 to 167.0 ppm (Figure S10).

**Table 1:**
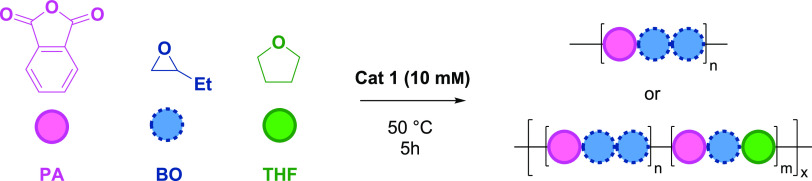
Ring-Opening Copolymerization (ROCOP) of Phthalic Anhydride (PA), Butylene Oxide (BO), and Tetrahydrofuran (THF) with Catalyst **1**^a^

polymer no.	starting monomer stoichiometry [Cat]/[PA]/[BO]/[THF]	degrees of polymerization (DP): [PA]/[BO]/[THF]^b^^,^^c^	polymer selectivity for BO [%]^c^	polymer selectivity for THF [%]^c^	*M*_n_ (*Đ*) [kg mol^–1^]^d^	*M*_n_ (NMR)^e^	*M*_n_ (theo)^f^	*T*_g_ (°C)^g^
**P1**	1:50:1150:0	50:110:0	69	0	8.2 (1.26)	7.5	7.7	4
**P2**	1:50:863:308	50:81:28	51	18	8.4 (1.17)	8.1	7.6	–6
**P3**	1:50:575:616	50:67:38	43	24	8.7 (1.15)	8.8	7.5	–8
**P4**	1:50:288:924	38:44:34	38	29	6.5 (1.18)	6.6	5.6	–8
**P5**	1:50:0:1233	0:0:0	N.D.	N.D.	N.D.	N.D.	N.D.	N.D.
**P6**^h^	1:50:50:0	<5:<5:0	N.D.	N.D.	N.D.	N.D.	N.D.	N.D.
**P7**^i^	1:100:800:0	60:60:0	50	0	6.7 (1.25)	6.4	7.1	41
**P8**^j^^,^(49)	1:250:250:0	250:250:0	50	0	14.7 (1.26)	N.D.	55.1	45

a ROCOP conditions: [**1**] = 10 mM, [PA] = 0.5 M, BO = 0–1 mL, THF = 0–1 mL, total volume of THF + BO = 1 mL, 50 °C, 5 h.

b DP of PA measured by integration of the aromatic resonances of PA (7.98 ppm) and P(PA) (7.59 ppm) in the ^1^H NMR spectra of crude polymers (Figure S8).

c Determined by integration of the ^1^H NMR spectra of purified polymer against P(PA) (Figures S9 and S18).

d Determined by gel permeation chromatography (GPC), using THF as the eluent, and calibrated using narrow MW polystyrene standards (Figure S19).

e Determined by integration of initiator −OCH(C*H*_3_)_2_ (1.32–1.36 ppm) in the ^1^H NMR spectra against the purified polymer (Figures S9 and S18).

f Theoretical *M*_n_ are calculated from the monomer conversion data and it is assumed that both isopropoxides initiate the reaction.

g Glass-transition temperature obtained by differential scanning calorimetry (DSC, second heating cycle at a heating rate of 10 °C min^–1^) (Figure 3D).

h ROCOP conditions: [**1**] = 10 mM, [PA] = 500 mM, [BO] = 500 mM, [PhMe] = 9.4 M (1 mL), 50 °C, 24 h.

i ROCOP conditions: [**CoK**] = 14 mM, [PA] = 1.43 M, [BO] = 16.9 M, 1 mL, 60 °C, 1 h (Chart S1).

j ROCOP conditions: [Cat] = 1:1 **CrSalen**: DMAP = 20 mM, [PA] = 5 M, [BO] = 5 M, [PhMe] = 9.4 M, 0.5 mL, 110 °C, 1 h (Chart S1).^49^

To understand the **ABB** polymer linkage selectivity, the polymerization was conducted with regular removal of aliquots (every 30 min). These samples were dried to remove any unreacted epoxide: the 2:1 ring-opened epoxide/anhydride selectivity remained constant throughout the polymerization, conveniently measured using PA and P(PA) as an internal standard (where P(PA) refers to a ring-opened PA unit in the polymer chain, Figures 3A and S20). No additional epoxide was polymerized once the anhydride was consumed. This result suggests that catalysis occurred selectively, i.e., rather than the formation of an ether end block or random incorporation of ether linkages, the catalyst produced polymers showing −(PA–BO-BO)– repeat units. Changing the relaxation times in the NMR spectroscopy experiments (*D*_1_ = 1–60 s) did not change the integrals, suggesting that the composition data could be reliably interpreted from relative integrals. The polymers were analyzed by gel permeation chromatography (GPC), which showed a steady evolution in molar mass with narrow, monomodal molar mass distributions (*Đ* = 1.11–1.26) throughout the polymerizations. Chains were initiated from the catalyst alkoxide groups, as confirmed by ^1^H NMR spectroscopy, where isopropoxide end-groups were clearly observed (Figures S9 and S13). Experimental molar mass values (obtained from both GPC and NMR) were in close agreement with theoretical values indicative of high initiator efficiency and controlled polymerizations.

**Figure 3:**
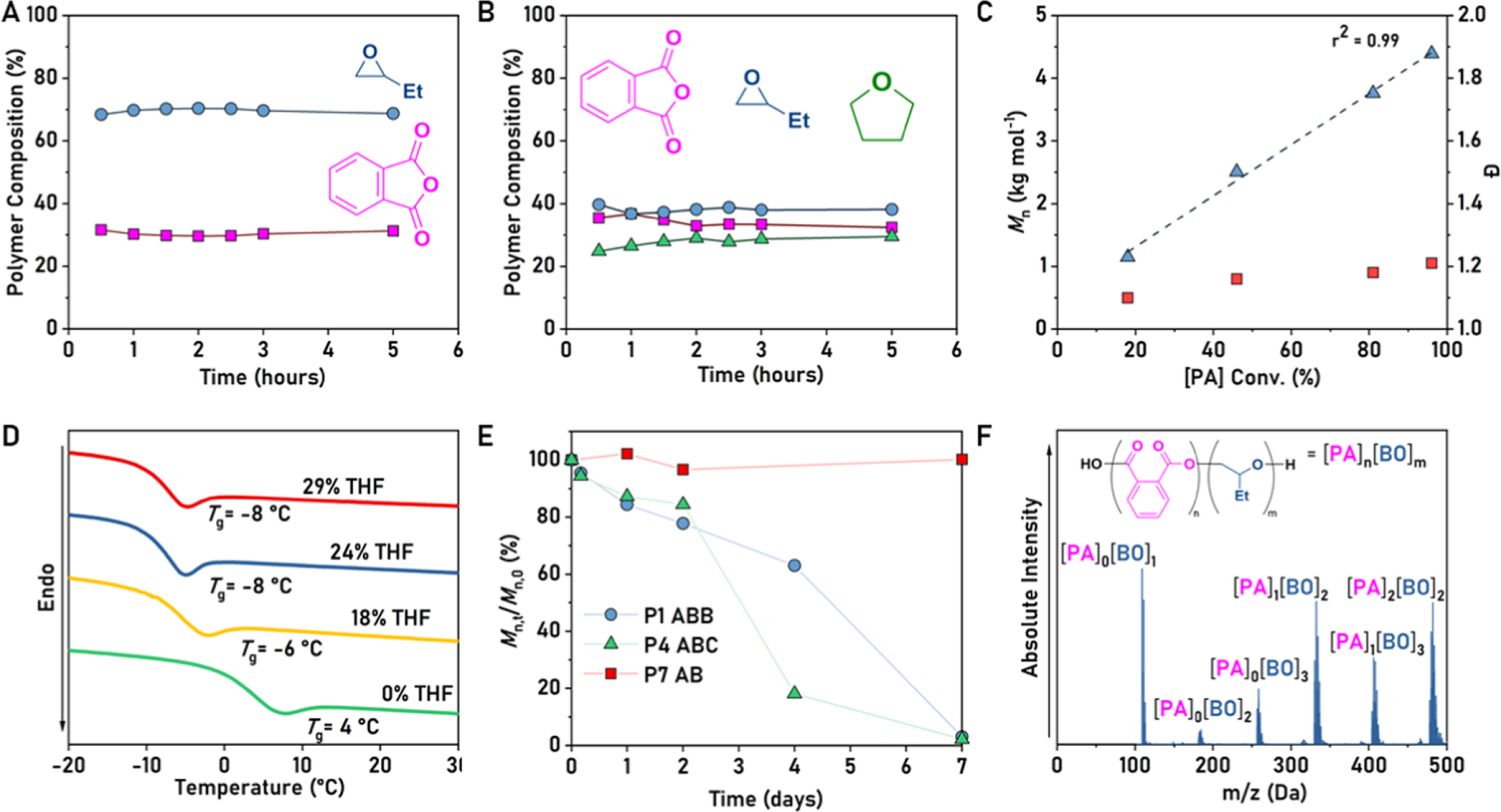
Selected data for **P1**–**P7**, showing polymer composition, molar mass, glass-transition temperature, and degradation rates and products. (A) **P1**, monitoring of PA and BO ROCOP over time with monomer conversions being determined by ^1^H NMR spectroscopy (Table 1, #1). (B) **P4**, monitoring of PA, BO, and THF ROCOP over time with monomer conversions being determined by ^1^H NMR spectroscopy (Table 1, #2). (C) **P1**, plot of the polymer molar mass (blue triangles) and dispersity (red squares) against phthalic anhydride conversion (Table 1, #1). (D) Normalized DSC data showing the changes to glass-transition temperature for **P1** (green), **P2** (yellow), **P3** (blue), and **P4** (red) (Table 1, #1–4). (E) Plot showing changes to polymer molar mass over time during alkaline degradation. Degradation profiles were compared for **P7** (P(PA-*alt*-BO), red squares), **P4** (green triangles), and **P1** (blue circles). Degradations were conducted by immersing polymers in KOH (5 M) at 70 °C over 7 days. (F) Mass spectrum of **P1** degradation products (TOF-MS-ESI mode, see Figure S25 for full spectra and details).

Polymerizations were next conducted using PA (**A**) and BO (**B**), dissolved in THF (**C**) to understand the influences of BO concentration on the polymer structures. The resulting polymer showed THF copolymerization and formed poly(ester-*alt*-ethers) with both **ABB** and **ABC** linkages (Table 1, #2–4). A series of polymerizations were conducted with variable quantities of BO and THF but at a constant overall concentration of catalyst and PA, i.e., constant total reaction volume. The resulting polymers were analyzed using ^1^H NMR spectroscopy and all showed similar poly(ester-*alt*-ethers) structures. All samples showed a constant ratio of **A**/[**B** + **C**], i.e., ester/ether repeat units. The monomer consumption over time was determined by regular aliquot analysis (Table 1, #4). The uptake of BO and THF was constant throughout the reaction, and there was no subsequent polymerization of either monomer once PA was consumed (Figure 3B). Similar to **P1**, the methine-ester (COOC*H*(CH_2_CH_3_)CH_2_−) and methylene-ester (COOC*H*_2_CH−) resonances for **P2**–**P4**, in the ^1^H NMR spectra, are at lower chemical shifts compared to the perfectly alternating polymer **P7** (**P2**–**P4** δ_H_ = 5.13 and 4.29 ppm vs **P7** δ_H_ = 5.31 and 4.43 ppm, respectively, Figures 2 and S13). For **P2**–**P4**, integration of the −CH_3_ resonance, assigned to ring-opened BO (δ_H_ = 0.96 ppm), against the methine-ester, and diagnostic aromatic resonances of ring-opened PA (δ_H_ = 7.67 and 7.45 ppm), shows reduced uptake of BO into the polymer with a lower initial concentration of BO in the monomer mixture. The ^1^H NMR spectra for **P2**–**P4** show similar chemical shifts but different peak shapes in the ether region compared to **P1**. The integrals for the ether region indicate that both BO and THF are enchained. Further, a new broad resonance was observed at 1.77 ppm, which is assigned to −OCH_2_C*H*_2_C*H*_2_CH_2_O–.

The polymerizations were well controlled as evidenced by a linear increase in molar mass (*M*_n_) vs time and narrow, monomodal distributions (Figures 3C, S22, and S23). The selectivity for THF uptake into the polymer backbones was always lower than that for BO even when excess THF was present in the starting mixtures (*S*(THF) ≤29%; *S*(BO) ≥38%, *S*(THF + BO) = 67–69%). To understand whether PA/THF ROCOP was feasible, polymerization was undertaken without any BO present but resulted in no polymer formation (Table 1, #5). The data are consistent with THF uptake only occurring during the second heterocycle insertion and with THF showing lower reactivity than BO in the ether forming step.

### Poly(ester-*alt*-ethers) Properties

All of the poly(esters-*alt*-ethers) are amorphous, as determined by DSC, and all showed significantly lower glass-transition temperatures compared to a perfectly alternating polymer analogue, i.e., P(PA-*alt*-BO) (*T*_g_ = 45 °C, *M*_n_ = 14.7 kg mol^–1^, Figure 3D).^49^ Poly(ester-*alt*-ethers) incorporating increasing THF linkages showed progressively lower *T*_g_ values (S(THF) = 18–29%, *T*_g_ = −6 to −8 °C). One limitation of semiaromatic polyesters, such as those produced by AB alternating ROCOP, is the relatively slow rate of ester hydrolysis.^50,51^ These rates can be overcome by conducting ester hydrolysis processes at above ambient temperature, such as those which might be proposed during chemical recycling.^50^ It is hypothesized that increasing the distance between ester linkages might increase degradation rates, especially if such linkers also increase the chain flexibility.^52^ Nonetheless, structure–degradation insights are limited by the lack of control in conventional polyester synthesis and the narrow differentiation of structures. A model set of degradation conditions were applied to allow for manageable hydrolysis and monitoring under laboratory conditions. As such, polymer suspensions were stirred in a basic aqueous solution (KOH = 5 M) at 70 °C (10 mg mL^–1^) with aliquots regularly removed for analysis using GPC (Figure 3E). The degradation reactions were benchmarked using the AB polymer, **P7**, i.e., P(BO-*alt*-PA), (*M*_n_ = 7.1 kg mol^–1^, *Đ* = 1.31). Under the reaction conditions, the alternating polyester did not degrade, remaining at the same molar mass over 7 days. In contrast, poly(ester-*alt*-ethers) with **ABB** and **ABC** sequences underwent complete degradation over the same period. The degradation products were analyzed for **P1** (**ABB**) to provide further evidence for the structures. After complete degradation, the reaction solution was neutralized (by adding HCl until pH = 7) and samples were analyzed using liquid chromatography–mass spectrometry (LC–MS) (Figures 3F and S24–S26). Analytes were observed in negative mode, and anions corresponding to monomers and dimers were observed in each case with the characteristic ionization patterns of diols (see the Supporting Information for further details, Table S2).^53^ The degradation products, although complex, were all consistent with the products expected after degradation of **ABB** sequences. All of the signals can be assigned to repeat units comprising a single PA and two BO monomers; note that the fragment with three BO units is consistent with BO–PA–BO–BO– fragmentation. No fragment for the perfectly alternating sequence, [PA]_1_[BO]_1_, is observed.

Finally, the thermal degradation of **P1**, **P4**, and **P7** was compared (Figure S27). **P1** and **P7** showed very similar, high thermal stability values (*T*_d,5_ = 318 and 309 °C, respectively). Both polymers are comprised of P(PA) and P(BO) units only, suggesting that the common ester linkages are cleaved during thermal degradation. In contrast, **P4**, comprised of 29% P(THF) units, showed lower stability (*T*_d,5_ = 194 °C). In comparison to poly(ester-*alt*-ether) PPDX, which thermally degrades at 180 °C,^7^**P1** has greater high temperature stability and would be the structure to target for processing investigations.

### Monomer Scope

Given that catalyst **1** shows unusual selectivity in PA/BO ROCOP, yielding poly(ester-*alt*-ethers) with **ABB** (or **ABC** for PA, BO, THF) sequences, it was of interest to investigate its performance using other monomers (Table 2). Polymerizations conducted using propylene oxide (PO) instead of BO, i.e., monomers = PA, PO, and THF, resulted in poly(ester-*alt*-ether) formation. Once again, the new polymers were characterized using ^1^H, ^13^C, and COSY NMR spectroscopies (Table 2, **P9**–**P12**). For example, **P9** shows the methine-ester (−COOC*H*(CH_3_)CH_2_−) and methylene-ester (−COOC*H*_2_CH−) resonances (δ_H_ = 5.27 and 4.22 ppm respectively) without any proton correlations (Figures S28–S35). All of the new polymers are amorphous and show glass-transition temperatures that get progressively lower with increasing THF selectivity (*T*_g_ = −3 to −5 °C, Figure S51). Bio-derived 2-methyl tetrahydrofuran (MeTHF), synthesized from sugars, was also investigated in reactions with PA and BO (Table 2, #6–8).^54^ Using this monomer likewise resulted in successful formation of poly(ester-*alt*-ethers) with up to 29% MeTHF incorporation (Figures S36–S39). Its NMR spectra show, in addition to the expected key assignments (no ^1^H–^1^H COSY correlation between the methine-ester and methylene-ester, δ_H_ = 5.13 and 4.35 ppm, respectively), the characteristic −CH_3_ resonance from ring-opened MeTHF at 1.20 ppm. Oxetane (OX), which has a lower ring strain than PO/BO but a higher ring strain than THF, is seldom investigated in ROCOP.^25,27^ Using the new Zr catalyst, the copolymerization of PA and OX is successful and gives poly(ester-*alt*-ether), i.e., poly(PA-OX-OX) (Table 2, **P15**). The polymer’s composition was confirmed by ^1^H NMR spectroscopy, for example, methylene-ester (−COOC*H*_2_CH−) and methylene-ether (−OC*H*_2_CH−) are observed at δ_H_ = 4.38 and 4.52 ppm, respectively, with ∼1:1 integrals. The polymer experimental molar mass values were in good agreement with theoretical values (Figures S40 and S41). For reactions between OX, THF, and PA, the selectivity for THF linkages was around 10%, which is lower than the equivalent reaction conducted using BO (Table 2, **P16** vs Table 1, **P4**, Figures S44–S47).

**Table 2:**
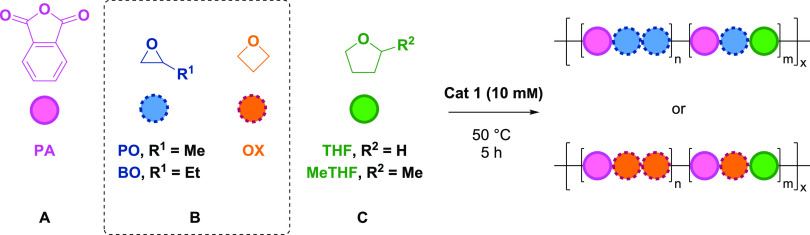
Ring-Opening Copolymerization of PA, Epoxides, Oxetane, and THF^a^

polymer no.	starting monomer stoichiometry [Cat]/[A]/[B]/[C]	degrees of polymerization (DP): [A]/[B]/[C]^b^^,^^c^	polymer selectivity for B [%]^c^	polymer selectivity for C [%]^c^	*M*_n_ (*Đ*) [kg mol^–1^]^d^	*M*_n_ (theo)^e^	*T*_g_ (°C)^f^
**[A]/[B]/[C] = [PA]/[PO]/[THF]**							
**P9**^g^	1:50:1419:0	33:75:0	69	0	3.2 (1.32)	4.6	7
**P10**	1:50:1064:308	50:88:17	57	11	7.1 (1.20)	6.9	–3
**P11**	1:50:710:616	50:67:40	43	26	6.7 (1.19)	7.1	–5
**P12**	1:50:355:924	50:63:46	40	29	8.3 (1.24)	7.2	–5
**[A]/[B]/[C] = [PA]/[BO]/[MeTHF]**							
**P13**	1:50:863:248	50:87:14	58	9	4.9 (1.17)	7.5	1
**P14**	1:50:288:744	50:60:39	40	26	6.1 (1.13)	8.5	1
**[A]/[B]/[C] = [PA]/[OX]/[THF]**							
**P15**	1:50:1527:0	43:90:0	68	0	2.3 (1.16)	5.8	–15
**P16**^h^	1:50:382:924	50:83:14	56	10	5.9 (1.05)	6.6	–16

a Conditions: [**1**] = 10 mM, [PA] = 0.5 M, **B** = 0.25–1 mL, **C** = 0–0.75 mL, total volume of **B** + **C** = 1 mL, 50 °C, 5 h.

b DP of PA measured by integration of the aromatic resonances of PA (7.98 ppm) and P(PA) (7.59 ppm) in the ^1^H NMR spectra of crude polymers (Figure S8).

c Determined by integration of the ^1^H NMR spectra of purified polymer against P(PA) (Figures S28–S47).

d Determined by gel permeation chromatography (GPC), using THF as the eluent, and calibrated using narrow MW polystyrene standards (Figures S48–S50).

e Theoretical *M*_n_ are calculated from the monomer conversion data, and it is assumed that both isopropoxides initiated the reaction.

f Glass-transition temperature obtained by DSC (second heating cycle at a heating rate of 10 °C min^–1^) (Figures S51–S53).

g Reaction stopped after 1 h.

h Reaction stopped after 18 h.

### Polymerization Kinetics

**Figure 4:**
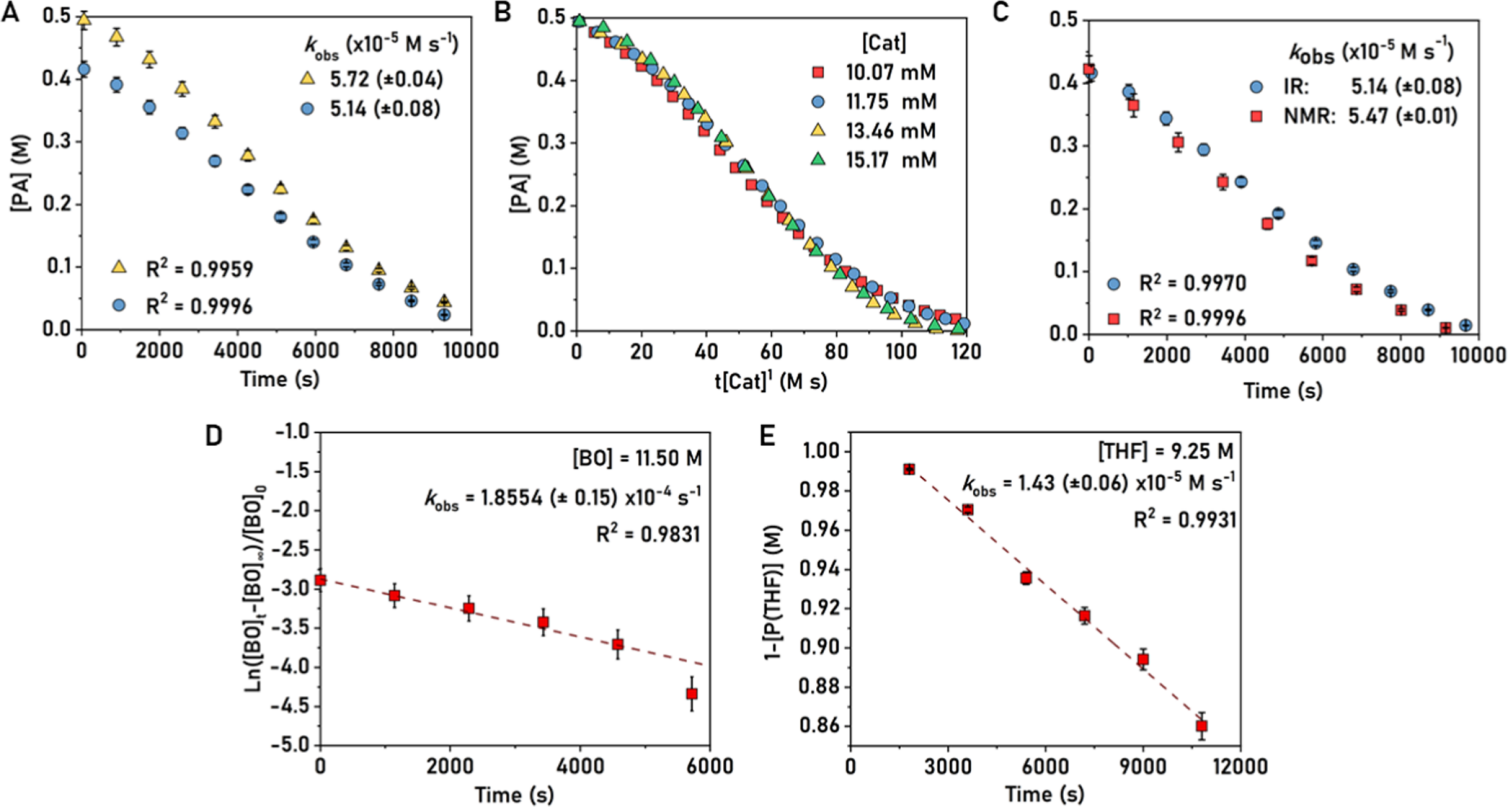
Polymerization kinetics for the ROCOP of PA, BO, and THF using catalyst **1**. (A) Plot of anhydride concentration vs time showing a linear fit to the data, [PA]_0_ = 0.42 (blue circles) or 0.48 M (yellow triangles), where [**1**] = 10 mM, [BO] = 11.50 M, and [THF] = 0 mM, 50 °C. (B) Plot of phthalic anhydride concentration vs catalyst t[catalyst]*^x^*, *x* = 1; the fit suggests a first order in catalyst concentration. [**1**] = 10.07 (red), 11.75 (blue), 13.46 (yellow), or 15.17 mM (green), where [PA] = 0.48 M, [BO] = 11.50 M, and [THF] = 0 mM, 50 °C. (C) Plots showing phthalic anhydride concentration vs time for polymerizations monitored using in situ IR (blue circles) or ^1^H NMR spectroscopy (red squares), where [**1**] = 10 mM, [PA] = 0.42 M, [BO] = 11.50 M, and [THF] = 0 mM, 50 °C. (D) Semilogarithmic plot of butylene oxide concentration vs time, with linear fit to the data, where [**1**] = 10 mM, [PA] = 0.42 M, [BO] = 11.50 M, and [THF] = 0 mM, 50 °C. (E) Plot of 1-P[THF] concentration vs time, where [**1**] = 10 mM, [PA] = 0.48 M, [BO] = 3.69 M, and [THF] = 9.25 mM, 50 °C.

To investigate the polymerization kinetics, catalyst **1** was reacted with PA and BO both with and without THF and using *in situ* IR spectroscopy to monitor changes in monomer concentration over time. To determine the order in PA concentration, its conversion against time data was acquired at two different starting concentrations, with all other species being held at a constant concentration (using attenuated total reflection-infrared (ATR-IR) spectroscopy, with data acquisition every 120 s, >90% conversion of PA, λ = 1779 cm^–1^); reactions were run in duplicate (Figure 4A). Both reactions show linear fits to the data, which are consistent with a rate law which is zero order in anhydride concentration. To determine the order in catalyst concentration, the evolution of PA concentration over time was assessed at four different catalyst concentrations, with otherwise identical conditions (Figure 4B). For each run, the PA concentration was plotted against catalyst normalized time, and the best fits were found for first orders in catalyst concentration. Alternative fits, trialing higher and lower catalyst orders, were much less effective (Figures S54 and S55). To measure the order in epoxide concentration, which does not absorb strongly in the IR spectroscopic region, *in situ*^1^H NMR spectroscopy was used. Polymerizations were conducted in a J Youngs Tap NMR tube with signals due to BO (δ_H_ = 3.00, 2.82 and 2.58 ppm) used for concentration vs time monitoring. To ensure that NMR spectroscopy was a reliable method, two polymerizations were compared under identical conditions: (1) Using IR spectroscopy (with stirring) and (2) using NMR spectroscopy (without stirring) (Figure 4C). There was a good agreement between the two rate constants, giving confidence that under the selected conditions, *in situ* NMR spectroscopy can be used to accurately determine rates. Polymerizations were subsequently conducted using four different epoxide concentrations (diluted with THF), but with otherwise identical conditions (Figures 4D, S56, and S57). In each case, the data showed exponential fits to conversion vs time data. Semilogarithmic plots of epoxide vs time are linear, consistent with a first order in epoxide concentration. It was not possible to directly measure the THF consumption due to overlapping resonances of THF and ring-opened THF in both IR and ^1^H/^2^D NMR spectra. However, carefully dried polymer samples analyzed by ^1^H NMR spectroscopy allowed determination of the ring-opened THF concentration (i.e., P(THF)) using PA/P(PA) as the internal standard (Figure 4E). Plots of 1-P[THF] against time showed linear fits, consistent with a zero order in THF concentration.

**Scheme 2:**
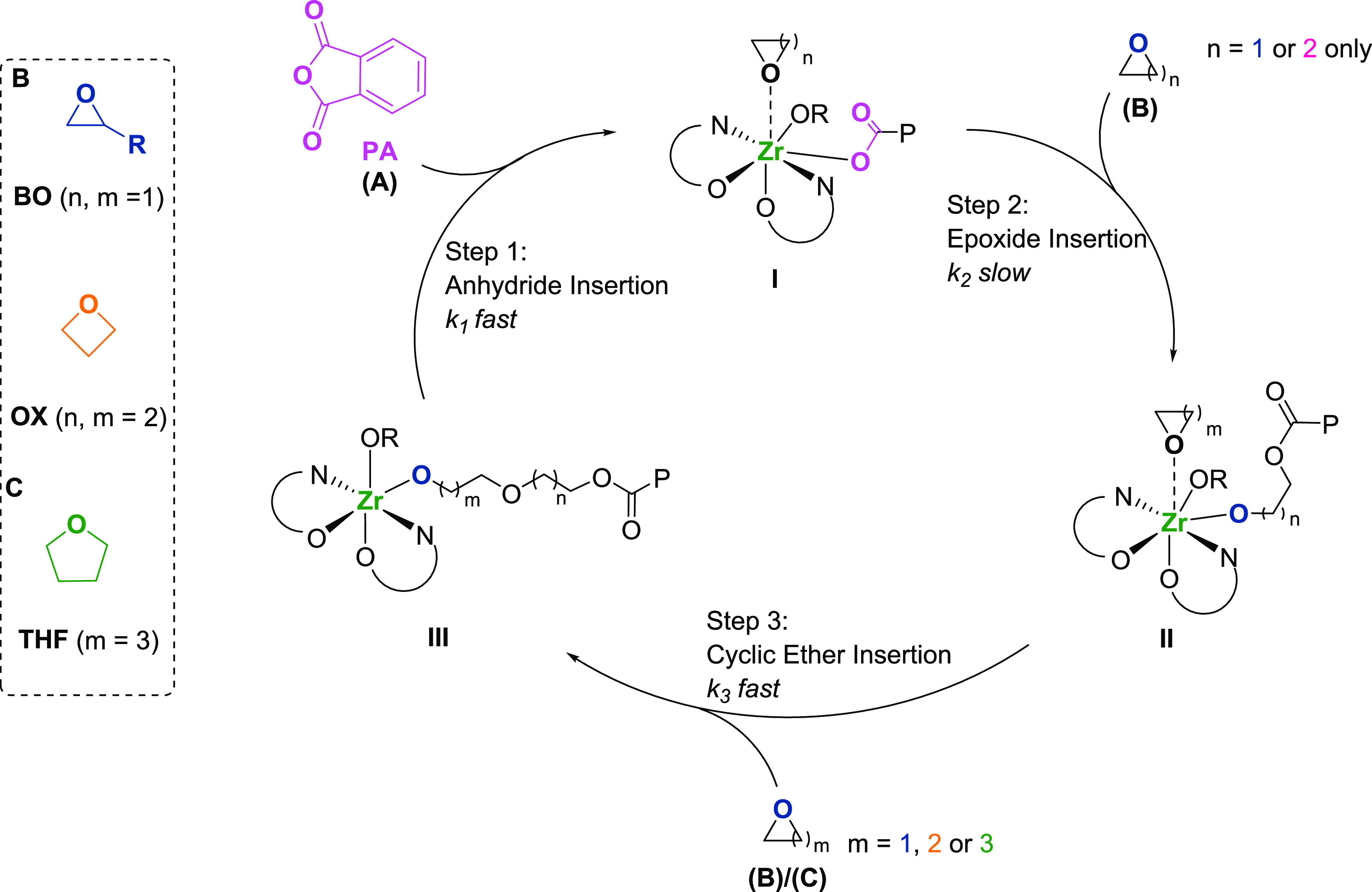
Proposed Catalytic Cycle for the Formation of Poly(ester-*alt*-ethers) by Ring-Opening Copolymerization of Phthalic Anhydride (PA), Cyclic Ethers (B), and Tetrahydrofuran (C) OR represents a second growing polymer chain.

Taken together, the kinetic data indicate that the rate-limiting step involves only the catalyst and the epoxide. Both polymerizations operate by the same second-order rate law, i.e., for both (i) PA and BO ROCOP and (ii) PA, BO, and THF ROCOP
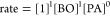
1
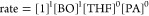
2The rate laws indicate that in all cases, the rate-limiting step involves butylene oxide-catalyzed ring opening. The limiting step was tentatively attributed to step 2 in the proposed catalytic cycle, i.e., Zr-carboxylate **I** undergoes nucleophilic attack on butylene oxide to form Zr-alkoxide intermediate **II** (Scheme 2). Accordingly, the other two steps in the catalytic cycle must occur faster to rationalize the zero-order rate dependencies in PA and THF. In epoxide/anhydride ROCOP catalysis, anhydride insertion is often zero order (fast).^16^ The finding that there is also a zero-order dependence on THF concentration, despite its high selectivity in step 3 (vide infra), suggests that the second alkoxide intermediate **III** is formed during a fast step (Scheme 2).

To investigate the proposed mechanism further, a series of stoichiometric reactions were performed. First, relevant to step 1, attempts were made to isolate Zr-carboxylate intermediate **I** (Section S3.3; Figures S58–S60). Catalyst **1** was reacted with stoichiometric quantities of PA in *d*_8_-toluene with the solvent being chosen to avoid any side reactions, e.g., solvent coordination. At 50 °C, no reaction occurred over 24 h, but at 80 °C and over 24 h, ∼50% of PA was consumed giving complex ^1^H and ^19^F NMR spectra. The species in solution are assigned as Zr-carboxylate aggregates and isomers; attempts to isolate them through crystallization only yielded unreacted PA. When the reaction mixture was analyzed using LC–MS signals consistent with isopropoxide, ring-opened PA was observed, i.e., the expected initiation product. Although we could not directly isolate Zr-carboxylate **I**, related Zr-Salan complexes featuring a tridentate-dicarboxylate co-ligand have been characterized in the solution and solid state (see Chart S2).^55^

To understand whether the Zr(IV) catalyst was capable of epoxide ROP (homopolymerization), **1** was reacted with neat BO, at 50 °C, over 24 h, but no polymer formed. Only when the reaction temperature was increased to 80 °C, poly(butylene oxide) begins to form but at a very low rate (TOF = 1 h^–1^). The comparative ROCOP catalysis shows a significantly higher TOF of 42 h^–1^ at 50 °C (Table 1, #1). Further, the ROP of THF did not occur even at 80 °C and over 24 h. These results show that while the catalyst can and does selectively form ether linkages during anhydride/epoxide copolymerization, the barriers to sequential epoxide enchainment without any anhydride present are prohibitive.

**Figure 5:**
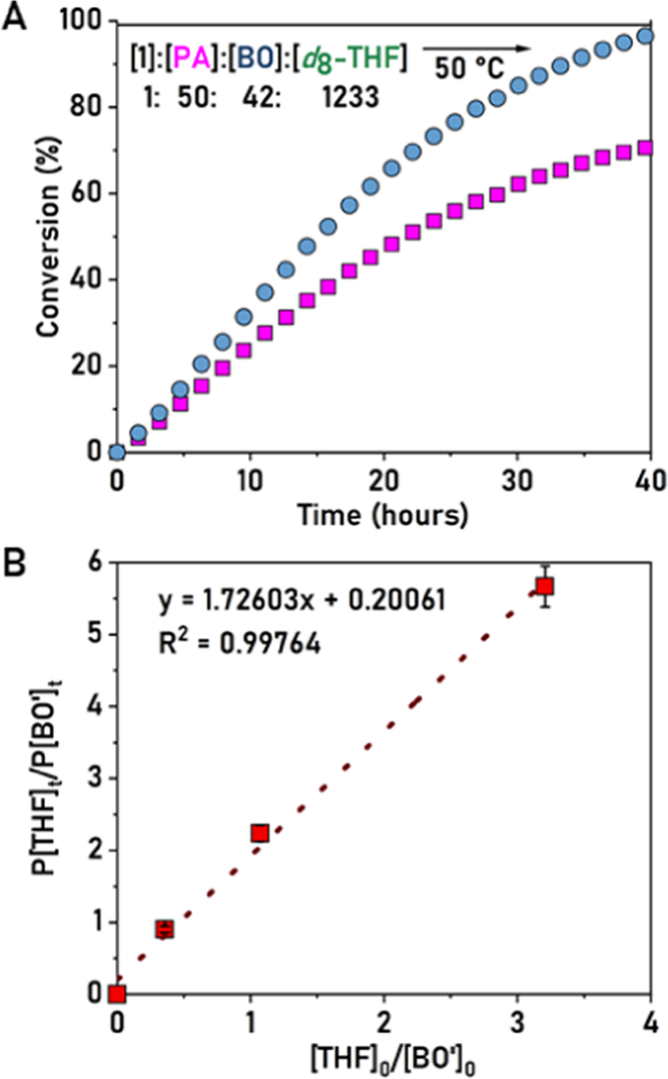
Top: Plot of time vs conversion of PA (pink squares) and substiochiometric BO (blue circles), where [**1**]/[PA]/[BO]/[*d*_8_-THF] = 1:50:42:1233. Bottom: Plot of final molar concentration of P[THF]/P[BO′] vs the initial molar concentration of [THF]/[BO′].

Next, step 2 was investigated by reacting PA with substoichiometric quantities of epoxide but with excess THF present. It is hypothesized that in step 2, the carboxylate intermediate **I** can only react with an epoxide. Polymerization using [**1**]/[PA]/[BO]/[*d*_8_-THF] = 1:50:42:1233 was monitored using *in situ*^1^H NMR spectroscopy (Figure 5A).

Over 40 h, complete epoxide consumption occurred but only ∼73% conversion of PA was observed, consistent with the limiting BO stoichiometry preventing complete PA consumption. Some *d*_8_-THF was also polymerized as would be expected (in step 3), but its conversion could not be quantified due to the overlapping monomer/polymer resonances. Further support for the hypothesis that only the epoxide is ring-opened in step 2 comes from the finding that the reaction of only PA and THF fails to yield any polymer (Table 1, #5).

In step 3, the alkoxide intermediate **II** reacts either with another BO or with THF to generate the second alkoxide intermediate **III**. The monomer selectivity in this step was compared against the starting monomer stoichiometries to estimate the relative reactivities (Figure 5B). To determine the selectivity for BO during step 3, the BO conversion during the second step must be accounted for. This is feasible by recognizing that in step 2, the same amount of BO is converted as PA. Thus, the conversion of BO in step 3 is given by [BO]–[PA], hence referred to as [BO′]. Plotting P[THF]*_t_*/P[BO′]*_t_* vs [THF]_0_/[BO′]_0_ resulted in data, which could be linearly fit, with a gradient 1.73. The linear relationship implies that there is ∼2-fold selectivity for THF over BO in the third step. It also shows that in step 3, selectivity correlates with the starting monomer concentrations.

Combining the rate law and experimental data, a mechanism for the poly(ester-*alt*-ethers) is proposed (Scheme 2). During initiation, the Zr-isopropoxide groups rapidly ring-open PA to form Zr-carboxylate intermediate **I**. The carboxylate intermediate ring-opens the epoxide to form Zr-alkoxide species **II**; this is the rate-determining step. In the case of anhydride/oxetane ROCOP, the Zr-carboxylate intermediate ring-opens the oxetane to generate a similar Zr-alkoxide intermediate. The alkoxide intermediate **II** does not react with PA but rather inserts an additional cyclic ether monomer, either an epoxide, oxetane, or THF molecule, to form the second alkoxide species **III**. The formation of the second alkoxide is also a fast step in the cycle. Next, intermediate **III** reacts with PA to form **ABB** or **ABC** sequence poly(ester-*alt*-ethers). It cannot be ruled out that a small proportion of intermediate **III** reacts with another cyclic ether to form low quantities of **ABBB** sequence errors, but such processes appear restricted to just one additional cyclic ether and do not involve extended poly(ether) sequences.

The Zr(IV) complex is a rare example of an anhydride/epoxide ROCOP catalyst that selectively yields **ABB** sequence selectivity. The only other catalysts showing similar behavior are an Al(III) alkoxide aggregate and a Sn(II) complex, comparably catalyst **1** is significantly more active and selective.^29,30^ For example, catalyst **1** achieves a TOF of 42 h^–1^ at a catalyst loading of 0.09 mol % ([1]/[PA]/[BO] = 1:50:1150, 50 °C, 1 h). The Al(III) cluster shows a 10-fold lower TOF of 4 h^–1^ at a much higher, 5 mol %, catalyst loading ([Al]/[PA]/[EO]/[THF] = 1:20:20:411, 3.5 h, 70 °C) and the Sn(II) catalyst shows a TOF of 2 h^–1^ at 1 mol % loading ([Sn]/[PA]/[CHO] = 1:50:100, 110 °C, 24 h).^29,30^ One rationale for the unusual selectivity of these catalysts is that the use of larger ionic radii metals, such as Zr(IV) or Sn(II), may facilitate growing polymer chain coordination back to metal, perhaps through ester moieties, which position the chain to stabilize **ABB** enchainment. In lactide ROP catalysis, such metallocyclic intermediates have long been known to be critical to mediating selectivity and activity.^56,57^

## Conclusions

In conclusion, a new Zr(IV) catalyst for epoxide and anhydride ROCOP shows good rates, unusual selectivity, and high polymerization control to produce poly(ester-*alt*-ethers). The catalysis applies commercially available monomers, such as phthalic anhydride, butylene oxide/oxetane, and tetrahydrofurans, and yields the alternating polymers with high selectivity. The colorless polymers are all amorphous with controllable low glass-transition temperatures, and the increased chain flexibility accelerates base-catalyzed degradation reactions. Polymerization kinetics and reactivity studies underpin a new type of **ABB** sequence control and polymerization mechanism. Further investigations of catalyst structure–activity/selectivity relationships, including catalysts featuring other larger ionic radius M(III)/M(IV) active sites, are in progress. These amorphous, “soft” polymers may be relevant in phase-separated block polymer structures, e.g., to make thermoplastic elastomers or plastomers.^50^ The Zr(IV) complex may also undergo switchable polymerization catalysis to access more complex polymer architectures and morphologies.^47,58,59^ In the biomedical context, these polymers could represent degradable alternatives to widely used polyethers.^60^

## Supporting Information Available

Characterization data of complexes (NMR, X-ray crystallography, elemental analysis) and polymerization data (GPC chromatograms, LC–MS) (PDF)

## Accession Codes

CCDC 2145249 contains the supplementary crystallographic data for this paper. These data can be obtained free of charge via www.ccdc.cam.ac.uk/data_request/cif, or by emailing data_request@ccdc.cam.ac.uk, or by contacting The Cambridge Crystallographic Data Centre, 12 Union Road, Cambridge CB2 1EZ, UK; fax: +44 1223 336033.

Crystal data (CCDC number: 2145249).

## Abbreviations

ROP: ring-opening polymerization
ROCOP: ring-opening copolymerization
PA: phthalic anhydride
EO: ethylene oxide
PO: propylene oxide
BO: butylene oxide
CHO: cyclohexene oxide
OX: oxetane
THF: tetrahydrofuran
MeTHF: 2-methyltetrahydrofuran
DP: degree of polymerization
TOF: turnover frequency

## References

[ref1] Häußler M.; Eck M.; Rothauer D.; Mecking S. Closed-loop recycling of polyethylene-like materials. Nature 2021, 590, 423–427. 10.1038/s41586-020-03149-9. 33597754

[ref2] Zhu Y.; Romain C.; Williams C. K. Sustainable polymers from renewable resources. Nature 2016, 540, 354–362. 10.1038/nature21001. 27974763

[ref3] Zhang X.; Fevre M.; Jones G. O.; Waymouth R. M. Catalysis as an Enabling Science for Sustainable Polymers. Chem. Rev. 2018, 118, 839–885. 10.1021/acs.chemrev.7b00329. 29048888

[ref4] Longo J. M.; Sanford M. J.; Coates G. W. Ring-Opening Copolymerization of Epoxides and Cyclic Anhydrides with Discrete Metal Complexes: Structure–Property Relationships. Chem. Rev. 2016, 116, 15167–15197. 10.1021/acs.chemrev.6b00553. 27936619

[ref5] Hillmyer M. A.; Tolman W. B. Aliphatic Polyester Block Polymers: Renewable, Degradable, and Sustainable. Acc. Chem. Res. 2014, 47, 2390–2396. 10.1021/ar500121d. 24852135

[ref6] Vilela C.; Sousa A. F.; Fonseca A. C.; Serra A. C.; Coelho J. F. J.; Freire C. S. R.; Silvestre A. J. D. The quest for sustainable polyesters – insights into the future. Polym. Chem. 2014, 5, 3119–3141. 10.1039/C3PY01213A.

[ref7] Raquez J.-M.; Degée P.; Narayan R.; Dubois P. Synthesis of melt-stable and semi-crystalline poly(1,4-dioxan-2-one) by ring-opening (co)polymerisation of 1,4-dioxan-2-one with different lactones. Polym. Degrad. Stab. 2004, 86, 159–169. 10.1016/j.polymdegradstab.2004.04.007.

[ref8] Raquez J.-M.; Degée P.; Narayan R.; Dubois P. Some Thermodynamic, Kinetic, and Mechanistic Aspects of the Ring-Opening Polymerization of 1,4-Dioxan-2-one Initiated by Al(O^*i*^Pr)_3_ in Bulk. Macromolecules 2001, 34, 8419–8425. 10.1021/ma010396e.

[ref9] Libiszowski J.; Kowalski A.; Szymanski R.; Duda A.; Raquez J.-M.; Degée P.; Dubois P. Monomer–Linear Macromolecules–Cyclic Oligomers Equilibria in the Polymerization of 1,4-Dioxan-2-one. Macromolecules 2004, 37, 52–59. 10.1021/ma030244e.

[ref10] Bechtold K.; Hillmyer M. A.; Tolman W. B. Perfectly Alternating Copolymer of Lactic Acid and Ethylene Oxide as a Plasticizing Agent for Polylactide. Macromolecules 2001, 34, 8641–8648. 10.1021/ma0114887.

[ref11] Uenishi K.; Sudo A.; Endo T. Anionic Alternating Copolymerizability of Epoxide and 3,4-Dihydrocoumarin by Imidazole. Macromolecules 2007, 40, 6535–6539. 10.1021/ma070433n.

[ref12] Van Zee N. J.; Coates G. W. Alternating copolymerization of dihydrocoumarin and epoxides catalyzed by chromium salen complexes: a new route to functional polyesters. Chem. Commun. 2014, 50, 6322–6325. 10.1039/c4cc01566e. 24806107

[ref13] Uenishi K.; Sudo A.; Endo T. Anionic alternating copolymerization of 3,4-dihydrocoumarin and glycidyl ethers: A new approach to polyester synthesis. J. Polym. Sci., Part A: Polym. Chem. 2008, 46, 4092–4102. 10.1002/pola.22752.

[ref14] Uenishi K.; Sudo A.; Endo T. Synthesis of polyester having sequentially ordered two orthogonal reactive groups by anionic alternating copolymerization of epoxide and bislactone. J. Polym. Sci., Part A: Polym. Chem. 2009, 47, 6750–6757. 10.1002/pola.23714.

[ref15] Paul S.; Zhu Y.; Romain C.; Brooks R.; Saini P. K.; Williams C. K. Ring-opening copolymerization (ROCOP): synthesis and properties of polyesters and polycarbonates. Chem. Commun. 2015, 51, 6459–6479. 10.1039/C4CC10113H. 25688813

[ref16] Plajer A. J.; Williams C. K. Heterocycle/Heteroallene Ring-Opening Copolymerization: Selective Catalysis Delivering Alternating Copolymers. Angew. Chem., Int. Ed. 2021, 61, e202104495 10.1002/anie.202104495. PMC9298364 34015162

[ref17] Liang X.; Tan F.; Zhu Y. Recent Developments in Ring-Opening Copolymerization of Epoxides With CO_2_ and Cyclic Anhydrides for Biomedical Applications. Front. Chem. 2021, 9, 647245 10.3389/fchem.2021.647245. 33959588 PMC8093832

[ref18] Winkler M.; Romain C.; Meier M. A. R.; Williams C. K. Renewable polycarbonates and polyesters from 1,4-cyclohexadiene. Green Chem. 2015, 17, 300–306. 10.1039/C4GC01353K.

[ref19] Sanford M. J.; Carrodeguas L. P.; Van Zee N. J.; Kleij A. W.; Coates G. W. Alternating Copolymerization of Propylene Oxide and Cyclohexene Oxide with Tricyclic Anhydrides: Access to Partially Renewable Aliphatic Polyesters with High Glass Transition Temperatures. Macromolecules 2016, 49, 6394–6400. 10.1021/acs.macromol.6b01425.

[ref20] Liu B.; Chen J.; Liu N.; Ding H.; Wu X.; Dai B.; Kim I. Bio-based polyesters synthesized by ring-opening copolymerizations of eugenyl glycidyl ether and cyclic anhydrides using a binuclear [OSSO]CrCl complex. Green Chem. 2020, 22, 5742–5750. 10.1039/D0GC00469C.

[ref21] Chen T. T. D.; Carrodeguas L. P.; Sulley G. S.; Gregory G. L.; Williams C. K. Bio-based and Degradable Block Polyester Pressure-Sensitive Adhesives. Angew. Chem., Int. Ed. 2020, 59, 23450–23455. 10.1002/anie.202006807. PMC7756385 32886833

[ref22] Tsuruta T.; Matsuura K.; Inoue S. Preparation of some polyesters by organometallic-catalyzed ring opening polymerization. Makromol. Chem. 1964, 75, 211–214. 10.1002/macp.1964.020750119.

[ref23] Stößer T.; Sulley G. S.; Gregory G. L.; Williams C. K. Easy access to oxygenated block polymers via switchable catalysis. Nat. Commun. 2019, 10, 2668 10.1038/s41467-019-10481-w. 31209211 PMC6572807

[ref24] Zustiak S. P.; Leach J. B. Hydrolytically Degradable Poly(Ethylene Glycol) Hydrogel Scaffolds with Tunable Degradation and Mechanical Properties. Biomacromolecules 2010, 11, 1348–1357. 10.1021/bm100137q. 20355705 PMC3050024

[ref25] Takeuchi D.; Aida T.; Endo T. The first example of the copolymerization of cyclic acid anhydrides with oxetane by bulky titanium bisphenolates. Macromol. Rapid Commun. 1999, 20, 646–649. 10.1002/(SICI)1521-3927(19991201)20:12<646::AID-MARC646>3.0.CO;2-N.

[ref26] McGuire T. M.; Clark E. F.; Buchard A. Polymers from Sugars and Cyclic Anhydrides: Ring-Opening Copolymerization of a d-Xylose Anhydrosugar Oxetane. Macromolecules 2021, 54, 5094–5105. 10.1021/acs.macromol.1c00365.

[ref27] Kameyama A.; Ueda K.; Kudo H.; Nishikubo T. The First Synthesis of Alternating Copolymers of Oxetanes with Cyclic Carboxylic Anhydrides Using Quaternary Onium Salts. Macromolecules 2002, 35, 3792–3794. 10.1021/ma020106+.

[ref28] Tang T.; Oshimura M.; Yamada S.; Takasu A.; Yang X.; Cai Q. Synthesis of periodic copolymers via ring-opening copolymerizations of cyclic anhydrides with tetrahydrofuran using nonafluorobutanesulfonimide as an organic catalyst and subsequent transformation to aliphatic polyesters. J. Polym. Sci., Part A: Polym. Chem. 2012, 50, 3171–3183. 10.1002/pola.26103.

[ref29] Hsieh H. L. Terpolymerization of Cyclic Ethers with Cyclic Anhydride. J. Macromol. Sci., Part A 1973, 7, 1525–1535. 10.1080/10601327308060517.

[ref30] Ungpittagul T.; Jaenjai T.; Roongcharoen T.; Namuangruk S.; Phomphrai K. Unprecedented Double Insertion of Cyclohexene Oxide in Ring-Opening Copolymerization with Cyclic Anhydrides Catalyzed by a Tin(II) Alkoxide Complex. Macromolecules 2020, 53, 9869–9877. 10.1021/acs.macromol.0c01738.

[ref31] Yuntawattana N.; Gregory G. L.; Carrodeguas L. P.; Williams C. K. Switchable Polymerization Catalysis Using a Tin(II) Catalyst and Commercial Monomers to Toughen Poly(l-lactide). ACS Macro Lett. 2021, 10, 774–779. 10.1021/acsmacrolett.1c00216. 34306820 PMC8296665

[ref32] Fieser M. E.; Sanford M. J.; Mitchell L. A.; Dunbar C. R.; Mandal M.; Van Zee N. J.; Urness D. M.; Cramer C. J.; Coates G. W.; Tolman W. B. Mechanistic Insights into the Alternating Copolymerization of Epoxides and Cyclic Anhydrides Using a (Salph)AlCl and Iminium Salt Catalytic System. J. Am. Chem. Soc. 2017, 139, 15222–15231. 10.1021/jacs.7b09079. 28984455

[ref33] Thevenon A.; Cyriac A.; Myers D.; White A. J. P.; Durr C. B.; Williams C. K. Indium Catalysts for Low-Pressure CO_2_/Epoxide Ring-Opening Copolymerization: Evidence for a Mononuclear Mechanism?. J. Am. Chem. Soc. 2018, 140, 6893–6903. 10.1021/jacs.8b01920. 29782169

[ref34] Diment W. T.; Gregory G. L.; Kerr R. W. F.; Phanopoulos A.; Buchard A.; Williams C. K. Catalytic Synergy Using Al(III) and Group 1 Metals to Accelerate Epoxide and Anhydride Ring-Opening Copolymerizations. ACS Catal. 2021, 11, 12532–12542. 10.1021/acscatal.1c04020.

[ref35] Liu J.; Bao Y.-Y.; Liu Y.; Ren W.-M.; Lu X.-B. Binuclear chromium–salan complex catalyzed alternating copolymerization of epoxides and cyclic anhydrides. Polym. Chem. 2013, 4, 1439–1444. 10.1039/C2PY20842C.

[ref36] Pappuru S.; Chakraborty D.; Ramkumar V.; Chand D. K. Ring-opening copolymerization of maleic anhydride or L-Lactide with tert-butyl glycidyl ether by using efficient Ti and Zr benzoxazole-substituted 8-Hydroxyquinolinate catalysts. Polymer 2017, 123, 267–281. 10.1016/j.polymer.2017.06.073.

[ref37] Saito J.; Mitani M.; Mohri J.-i.; Yoshida Y.; Matsui S.; Ishii S.-i.; Kojoh S.-i.; Kashiwa N.; Fujita T. Living Polymerization of Ethylene with a Titanium Complex Containing Two Phenoxy-Imine Chelate Ligands. Angew. Chem., Int. Ed. 2001, 40, 2918–2920. 10.1002/1521-3773(20010803)40:15<2918::AID-ANIE2918>3.0.CO;2-S. 11500909

[ref38] Matsui S.; Mitani M.; Saito J.; Tohi Y.; Makio H.; Matsukawa N.; Takagi Y.; Tsuru K.; Nitabaru M.; Nakano T.; Tanaka H.; Kashiwa N.; Fujita T. A Family of Zirconium Complexes Having Two Phenoxy–Imine Chelate Ligands for Olefin Polymerization. J. Am. Chem. Soc. 2001, 123, 6847–6856. 10.1021/ja0032780.

[ref39] Hustad P. D.; Tian J.; Coates G. W. Mechanism of Propylene Insertion Using Bis(phenoxyimine)-Based Titanium Catalysts: An Unusual Secondary Insertion of Propylene in a Group IV Catalyst System. J. Am. Chem. Soc. 2002, 124, 3614–3621. 10.1021/ja0122593. 11929251

[ref40] Edson J. B.; Wang Z.; Kramer E. J.; Coates G. W. Fluorinated Bis(phenoxyketimine)titanium Complexes for the Living, Isoselective Polymerization of Propylene: Multiblock Isotactic Polypropylene Copolymers via Sequential Monomer Addition. J. Am. Chem. Soc. 2008, 130, 4968–4977. 10.1021/ja077772g. 18345670

[ref41] Matsugi T.; Fujita T. High-performance olefin polymerization catalysts discovered on the basis of a new catalyst design concept. Chem. Soc. Rev. 2008, 37, 1264–1277. 10.1039/b708843b. 18497937

[ref42] Suzuki Y.; Kinoshita S.; Shibahara A.; Ishii S.; Kawamura K.; Inoue Y.; Fujita T. Trimerization of Ethylene to 1-Hexene with Titanium Complexes Bearing Phenoxy–Imine Ligands with Pendant Donors Combined with MAO. Organometallics 2010, 29, 2394–2396. 10.1021/om1003368.

[ref43] Gao Y.; Christianson M. D.; Wang Y.; Chen J.; Marshall S.; Klosin J.; Lohr T. L.; Marks T. J. Unexpected Precatalyst σ-Ligand Effects in Phenoxyimine Zr-Catalyzed Ethylene/1-Octene Copolymerizations. J. Am. Chem. Soc. 2019, 141, 7822–7830. 10.1021/jacs.9b01445. 31017398

[ref44] Thevenon A.; Garden J. A.; White A. J. P.; Williams C. K. Dinuclear Zinc Salen Catalysts for the Ring Opening Copolymerization of Epoxides and Carbon Dioxide or Anhydrides. Inorg. Chem. 2015, 54, 11906–11915. 10.1021/acs.inorgchem.5b02233. 26605983

[ref45] Durr C. B.; Williams C. K. New Coordination Modes for Modified Schiff Base Ti(IV) Complexes and Their Control over Lactone Ring-Opening Polymerization Activity. Inorg. Chem. 2018, 57, 14240–14248. 10.1021/acs.inorgchem.8b02271. 30376308

[ref46] Rae A.; Gaston A. J.; Greindl Z.; Garden J. A. Electron rich (salen)AlCl catalysts for lactide polymerisation: Investigation of the influence of regioisomers on the rate and initiation efficiency. Eur. Polym. J. 2020, 138, 109917 10.1016/j.eurpolymj.2020.109917.

[ref47] Diment W. T.; Stößer T.; Kerr R. W. F.; Phanopoulos A.; Durr C. B.; Williams C. K. Ortho-vanillin derived Al(iii) and Co(iii) catalyst systems for switchable catalysis using ε-decalactone, phthalic anhydride and cyclohexene oxide. Catal. Sci. Technol. 2021, 11, 1737–1745. 10.1039/D0CY02164D.

[ref48] Shannon R. D. Revised effective ionic radii and systematic studies of interatomic distances in halides and chalcogenides. Acta Crystallogr., Sect. A 1976, 32, 751–767. 10.1107/S0567739476001551.

[ref49] Bester K.; Bukowska A.; Myśliwiec B.; Hus K.; Tomczyk D.; Urbaniak P.; Bukowski W. Alternating ring-opening copolymerization of phthalic anhydride with epoxides catalysed by salophen chromium(iii) complexes. An effect of substituents in salophen ligands. Polym. Chem. 2018, 9, 2147–2156. 10.1039/C8PY00048D.

[ref50] Gregory G. L.; Sulley G. S.; Carrodeguas L. P.; Chen T. T. D.; Santmarti A.; Terrill N. J.; Lee K.-Y.; Williams C. K. Triblock polyester thermoplastic elastomers with semi-aromatic polymer end blocks by ring-opening copolymerization. Chem. Sci. 2020, 11, 6567–6581. 10.1039/D0SC00463D. 34094122 PMC8159401

[ref51] Bornscheuer U. T. Feeding on plastic. Science 2016, 351, 1154–1155. 10.1126/science.aaf2853. 26965614

[ref52] Kricheldorf H. R.; Wahlen L.; Stukenbrook T. Biodegradable liquid-crystalline aromatic polyesters. Macromol. Symp. 1998, 130, 261–270. 10.1002/masy.19981300123.

[ref53] Schug K.; McNair H. M. Adduct formation in electrospray ionization. Part 1: Common acidic pharmaceuticals. J. Sep. Sci. 2002, 25, 759–766. 10.1002/1615-9314(20020801)25:12<759::AID-JSSC760>3.0.CO;2-M.

[ref54] Stadler B. M.; Tin S.; Kux A.; Grauke R.; Koy C.; Tiemersma-Wegman T. D.; Hinze S.; Beck H.; Glocker M. O.; Brandt A.; de Vries J. G. Co-Oligomers of Renewable and “Inert” 2-MeTHF and Propylene Oxide for Use in Bio-Based Adhesives. ACS Sustainable Chem. Eng. 2020, 8, 13467–13480. 10.1021/acssuschemeng.0c04450.

[ref55] Schneider F.; Zhao T.; Huhn T. Cytotoxic heteroleptic heptacoordinate salan zirconium(iv)-bis-chelates – synthesis, aqueous stability and X-ray structure analysis. Chem. Commun. 2016, 52, 10151–10154. 10.1039/C6CC05359A. 27459052

[ref56] Chamberlain B. M.; Cheng M.; Moore D. R.; Ovitt T. M.; Lobkovsky E. B.; Coates G. W. Polymerization of Lactide with Zinc and Magnesium β-Diiminate Complexes: Stereocontrol and Mechanism. J. Am. Chem. Soc. 2001, 123, 3229–3238. 10.1021/ja003851f. 11457057

[ref57] Wang L.; Kefalidis C. E.; Sinbandhit S.; Dorcet V.; Carpentier J.-F.; Maron L.; Sarazin Y. Heteroleptic Tin(II) Initiators for the Ring-Opening (Co)Polymerization of Lactide and Trimethylene Carbonate: Mechanistic Insights from Experiments and Computations. Chem. – Eur. J. 2013, 19, 13463–13478. 10.1002/chem.201301751. 23955851

[ref58] Stößer T.; Williams C. K. Selective Polymerization Catalysis from Monomer Mixtures: Using a Commercial Cr-Salen Catalyst To Access ABA Block Polyesters. Angew. Chem., Int. Ed. 2018, 57, 6337–6341. 10.1002/anie.201801400. PMC6391957 29518288

[ref59] Stößer T.; Mulryan D.; Williams C. K. Switch Catalysis To Deliver Multi-Block Polyesters from Mixtures of Propene Oxide, Lactide, and Phthalic Anhydride. Angew. Chem., Int. Ed. 2018, 57, 16893–16897. 10.1002/anie.201810245. PMC6391959 30370965

[ref60] Zhu Y.; Poma A.; Rizzello L.; Gouveia V. M.; Ruiz-Perez L.; Battaglia G.; Williams C. K. Metabolically Active, Fully Hydrolysable Polymersomes. Angew. Chem., Int. Ed. 2019, 58, 4581–4586. 10.1002/anie.201814320. PMC6492077 30720233

