# Supplementary material for: Zr(IV) Catalyst for the Ring-Opening Copolymerization of Anhydrides (A) with Epoxides (B), Oxetane (B), and Tetrahydrofurans (C) to Make ABB- and/or ABC-Poly(ester-alt-ethers)
Source: J Am Chem Soc. 2022 Apr 7;144(15):6882–93. doi: 10.1021/jacs.2c01225 (PMC9084548; doi:10.1021/jacs.2c01225)
Supplement: Supplementary file 1 — ja2c01225_si_001.pdf [file ja2c01225_si_001.pdf]

Supporting Information

Zr(IV) Catalyst for the Ring Opening Copolymerization of Anhydrides (A) with Epoxides (B), Oxetane (B) and Tetrahydrofurans (C) to Make ABB- and/or ABC- Poly(ester-*alt*-ethers)

Ryan W. F. Kerr, Charlotte K. Williams*

University of Oxford, Chemistry Research Laboratory, 12 Mansfield Road, Oxford OX1 3TA, UK.

General Methods 3
Materials 3
Methods 3
1.0 Catalyst Synthesis and Characterization 5
1.1.1 Attempted Synthesis of $[(L^{Me})Zr(O^iPr)_2]$ complexes 5
1.1.2 (*E*)-2-(((2,6-diisopropylphenyl)imino)methyl)-6-(trifluoromethoxy)phenol – HL_1 5
1.1.3 $[(L^1)_2Zr(O^iPr)_2]$ – 1 6
2.0 Ring-opening Copolymerization of Phthalic Anhydride, Oxiranes, Oxetanes and Tetrahydrofurans 10
2.1 Polymerization techniques 10
2.1.1 Sealed vial method: 10
2.1.2 ReactIR Spectroscopy method: 10
2.1.3 NMR Spectroscopy method: 10
2.2 NMR Spectroscopy Data Handling Procedures, relevant to entries in Table 1. 12
2.2.1 Crude NMR Spectra for P1 (PA-BO-BO) 13
2.2.2 NMR Spectra for P1, P(PA-BO-BO) (Table 1) 13
2.2.2 NMR Spectra for P4, P(PA-BO-BO)-(PA-BO-THF) (Table 1) 16
2.2.3 NMR Spectra for P7, P(PA-BO) (Table 1) 19
2.3 GPC Data for P1-4 (Table 1) 20
2.4 ^{1}H NMR Spectra of P1 reaction aliquots used to determine polymer composition versus time 21
2.5 GPC Data Showing changes to molar mass over time, during polymer formation 22
2.6 Polymer Degradation Experiments 23
2.6.1 LCMS Data from the Degradation of P1 (Table 1) 23
2.7 TGA Experiments for P1, P4 and P7 (Table 1) 27
2.8 NMR Spectra of Polymers Described in Table 2 28
2.8.1 NMR Spectra for P9, P(PA-PO-PO) (Table 2) 28
2.8.2 Spectra for P12, P(PA-PO-PO)-(PA-PO-THF) (Table 2) 30
2.8.3 Spectra for P14 P(PA-BO-BO)-(PA-BO-MeTHF) (Table 2) 32
2.8.4 Spectra of P15 P(PA-OX-OX) (Table 2) 34
2.8.5 Spectra for P16 P(PA-OX-OX)-(PA-OX-THF) (Table 2) 36
2.9 GPC Data for Polymers Described in Table 2 38
2.10 DSC Data for Polymers Described in Table 2 39
3.0 Kinetic Experiments 41
3.1 Alternative fits for order in catalyst 41
3.2 First Order Fits for Epoxide Concentration Dependence 42
3.3 Reaction of 1 and Phthalic Anhydride 43
4.0 Single Crystal X-ray Diffraction Data for Complex 1 45
5.0 References 46

General Methods

Materials

All solvents and reagents were purchased and used as obtained from commercial sources (Sigma Aldrich, Fluorochem and Apollo Scientific) unless stated otherwise. The synthesis of HL^{Me} was performed using literature procedures.[1] 2-Hydroxy-3-(trifluoromethoxy) benzaldehyde was stored under an inert (N_2) atmosphere until used. Commercial diisopropylaniline was fractionally distilled and was stored under an inert (N_2) atmosphere until used. All air-sensitive and moisture-sensitive reactions (attempted synthesis of $[(L^{Me})Zr(O^iPr)_2]$, synthesis of **1**, monomer purification, and polymerizations) were carried out under inert conditions, using standard Schlenk line techniques and a N_2-filled glovebox, unless otherwise stated. Propylene oxide (PO), butylene oxide (BO), oxetane (OX) were dried by stirring over CaH_2, followed by fractional distillation, samples were de-gassed by freeze-pump-thaw in triplicate and stored under an inert (N_2) atmosphere. 2-Methyltetrahydrofuran (MeTHF), and $CDCl_3$ were dried by stirring over CaH_2, followed by vacuum transfer, samples were degassed by freeze-pump-thaw in triplicate and stored over 3 Å molecular sieves under an inert (N_2) atmosphere. Tetrahydrofuran (THF), toluene, dichloromethane, chloroform and hexane were purified using an MB SPS800 solvent purification system, solvents were degassed by freeze-pump-thaw in triplicate and stored over 3 Å molecular sieves under an inert (N_2) atmosphere. Phthalic anhydride (PA) was purified by recrystallization from chloroform and sublimation in triplicate. The synthesis of **CoK** was performed using literature procedures.[2]

Methods

NMR Spectroscopy: ^1H, ^{13}C and ^{19}F NMR spectra were obtained using Bruker AV 400 MHz and 500 MHz instruments. In order to establish reproducible analysis of crude and pure polymers by ^1H NMR spectroscopy, sample **P1** was measured with a D_1 = 1 s, 15 s and 60 s. No differences between relative aryl/aliphatic integrals were found, therefore D_1 = 1 s was used for all ^1H NMR experiments, unless otherwise stated.

Gel Permeation Chromatography: GPC analysis was carried out using a Shimadzu LC-20AD instrument, equipped with PSS SDV 5 µm pre-column and two PSS SDV 5 µm linear M columns and a Refractive Index (RI) detector. Samples were dissolved in HPLC grade THF, filtered through 0.2 µm PTFE filters (VWR) and measurements were determined at 1 mL min^{-1} flow rate, at 30 °C. Monodisperse polystyrene standards were used to calibrate the instrument.

Elemental analyses were performed by Elemental Microanalysis Ltd, Hameldown Road, Okehampton Business Park, Exeter Road, Okehampton, Devon, EX20 1UB, UK

DSC: A sealed, empty crucible was used as a reference, and the DSC was calibrated using zinc and indium. Samples were heated from -50 °C to 100 °C, at a rate of 10 °C min^{-1} under N_2 flow (80 mL $min^{-1)}$. Samples were subsequently cooled to -50 °C, at a rate of 10 °C min^{-1}, and kept at -50 °C for a further 5 minutes, followed by a heating-cooling procedure from -50 °C to 100 °C, at a rate of 10 °C min^{-1}. Each sample was analyzed over two heating-cooling cycles. Glass transition temperatures (T_g) are reported as the midpoint of the transition taken from the second heating cycle.

TGA: Results were collected from Mettler-Toledo Ltd TGA/DSC 1 system. Powder polymer samples were heated from 30 to 500 °C at a rate of 5 °C min^{-1}, under N_2 flow (100 mL min^{-1}). Samples were held at 100 °C for 30 mins to remove potential residual solvent.

High-resolution mass spectra (ESI): Samples were recorded on a Bruker µTOF mass spectrometer, with 10 µg of sample dissolved in 1 mL of MeOH.

Liquid Chromatography Mass Spectrometry (LC-MS) analysis: The degraded polymers were analysed using a Waters LCT Premier bench-top orthogonal acceleration time-of-flight LC-MS system, connected to a CTC Analytics HTS PAL Sample Manager, Acquity PDA Detector, Acquity Column Heater/Cooler and Acquity Binary Solvent Manager.

1.0 Catalyst Synthesis and Characterization

1.1.1 Attempted Synthesis of [(L^{Me})Zr(O^iPr)$_2$] complexes

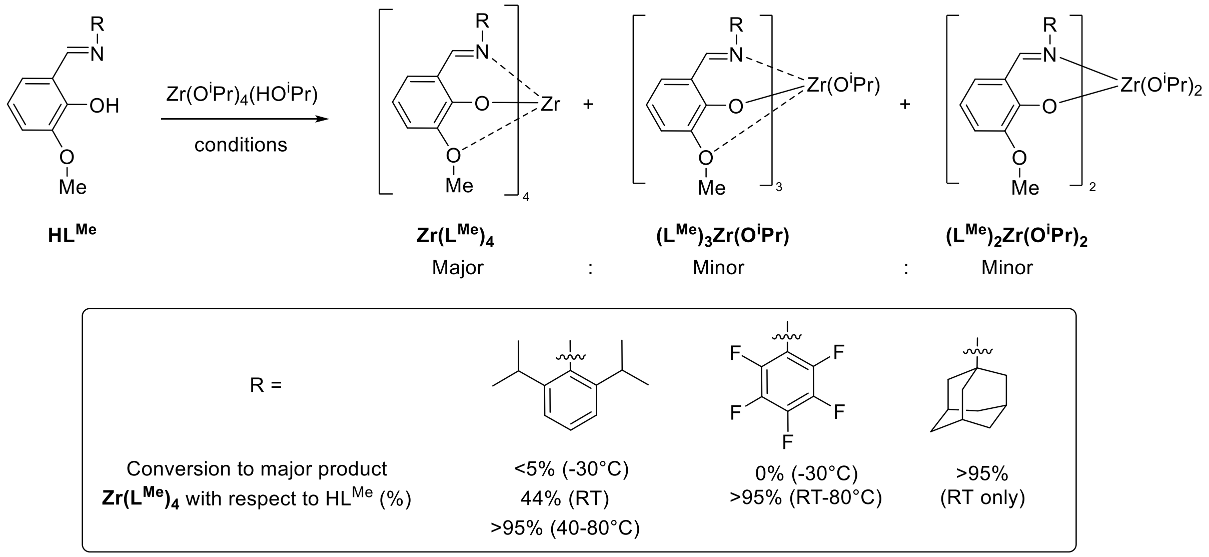

Using a modified literature procedure;[1] To an ampoule charged with HL^{Me} (1 mmol) dissolved in toluene (10 mL) was added [$Zr(O^iPr)_4(HO^iPr)$] (193 mg, 0.5 mmol) and stirred for 24 h, either at -30 °C, RT, 40 or 80 °C. At –30 °C and RT, low conversion (0-10 %) was observed by ^{1}H NMR spectroscopy. At 40 or 80 °C, a complex mixture of products was observed, with the major product assigned as [$Zr(L^{Me})_4$] and other species assigned as [$(L^{Me})_3Zr(O^iPr)$], [$(L^{Me})_2Zr(O^iPr)_2$] and unreacted [$Zr(O^iPr)_4(HO^iPr)$]. In each case, the reaction mixture was dried, and recrystallization was attempted with hexane/THF or hexane/DCM mixtures, at –30 °C, to try to isolate pure product. In all cases, yellow powders with the same composition as the crude product were isolated.

1.1.2 (*E*)-2-(((2,6-diisopropylphenyl)imino)methyl)-6-(trifluoromethoxy)phenol – HL_1

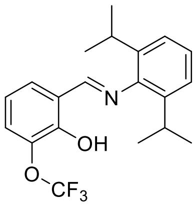

To a round bottom flask charged with 2-hydroxy-3-(trifluoromethoxy) benzaldehyde (500 mg, 2.43 mmol), dissolved in ethanol (15 mL), was added diisopropylaniline (430 mg, 2.43 mmol) and formic acid (0.02 mmol). The reaction was heated to reflux for 18 h, cooled to RT and concentrated. The yellow residue was dissolved in chloroform (3 mL) and recrystallized to give a yellow gum. The title was product was obtained as a yellow microcrystalline powder by washing the residue with minimal (drops of) cold EtOH (50 %, 439 mg, 1.2 mmol); **^{1}H NMR** (400 MHz, $CDCl_3$) δ_H: 13.67 (1H, s, Ar^1(O<u>H</u>)), 8.33 (1H, s, Ar^1C<u>H</u>=NAr^2), 7.49–7.37 (1H, m, Ar^1(5)<u>H</u>)), 7.36–7.31 (1H, dd, *J* 7.8, Ar^1(3)<u>H</u>)), 7.25–7.16 (3H, m, Ar^2(3,4,5)<u>H</u>), 6.96 (1H, t, *J* 7.8, Ar^1(4)<u>H</u>), 2.98 (2H, h, *J* 6.9 Ar^2(2,6)C<u>H</u>(CH_3)$_2$), 1.19 (12H, d, *J* 6.9, Ar^2(2,6)CH(C<u>H</u>$_3$)$_2$); **^{19}F NMR** (376 MHz, $CDCl_3$) δ_F: –58.04–(–)58.11 (m). **HRMS (ESI)**: ($[M+H]^+$) calculated for $C_{20}H_{23}F_3N_1O_2$: 366.1675; found: 366.1675.

1.1.3 $[(L^1)_2Zr(O^iPr)_2]$ – 1

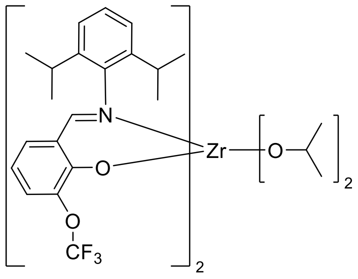

To an ampoule charged with **HL¹** (365 mg, 1 mmol) dissolved in toluene (10 mL) was added $[Zr(O^iPr)_4(HO^iPr)]$ (193 mg, 0.5 mmol) and stirred for 24 h, at 40 °C. The reaction mixture was dried, and recrystallized in hexane with a drop of THF (5 mL), at –30 °C, overnight, to give the title product as a pale yellow powder (43 %, 201 mg, 0.22 mmol); **^{1}H NMR** (400 MHz, $CDCl_3$) δ_H: 8.12 (2H, s, CH=NAr), 7.32 (2H, d, *J* 7.8, $Ar^1(3)H$), 7.29–7.19 (6H, m, Ar^2H), 7.15 (2H, d, *J* 7.8, $Ar^1(5)H$), 6.61 (2H, t, *J* 7.8, $Ar^1(4)H$), 3.61 (2H, p, *J* 6.8, $ArCH(CH_3)_2$), 3.44 (2H, p, *J* 6.7, $ArCH(CH_3)_2$), 2.92 (2H, p, *J* 6.2, $ZrOCH(CH_3)_2$), 1.45–1.24 (12H, m, $ArCH(CH_3)_2$), 1.06 (12H, dd, *J* 18.7, 6.7, $ArCH(CH_3)_2$), 0.52 (12H, dd, *J* 37.4, 6.2, $Zr(OCH(CH_3)_2)_2$); **^{13}C NMR** (126 MHz, $CDCl_3$) δ_C: 171.9 ($Ar^1CH=NAr^2$), 158.8 ($Ar^1(1)C$), 150.5 ($Ar^2(1)C$), 142.1 ($Ar^2(2)C$), 140.8 ($Ar^2(6)C$), 139.0 $Ar^1(6)C$, 134.3 ($Ar^1(5)C$), 129.7 ($Ar^1(3)C$), 126.8 ($Ar^2(4)C$), 124.1 ($Ar^2(3)C$), 123.9 ($Ar^2(5)C$), 122.7 ($Ar^1(2)C$), 120.9 (q, *J* 257.4, $ArOCF_3$), 114.6 ($Ar^1(4)C$), 70.2 $Zr(OCH(CH_3)_2)$, 27.8 ($Ar^2(5)CCH(CH_3)_2$), 27.5 ($Ar^2(2)CCH(CH_3)_2$), 25.9 ($Ar^2(2)CCH(CH_3)_a(CH_3)_b$), 25.7 ($Ar^2(6)CCH(CH_3)_a(CH_3)_b$), 25.5 ($Zr(OCH(CH_3)_a(CH_3)_b)$, 25.4 ($Zr(OCH(CH_3)_a(CH_3)_b)$, 22.9 ($Ar^2(2)CCH(CH_3)_a(CH_3)_b$), 22.8 ($Ar^2(6)CCH(CH_3)_a(CH_3)_b$). **^{19}F NMR** (376 MHz, $CDCl_3$) δ_F: –58.43. **Elemental analysis** $C_{36}H_{56}F_6N_2O_6Zr_1$: C 58.89%, H 6.02%, N 2.99% calculated. C 58.93%, H 5.87%, N 3.16% found.

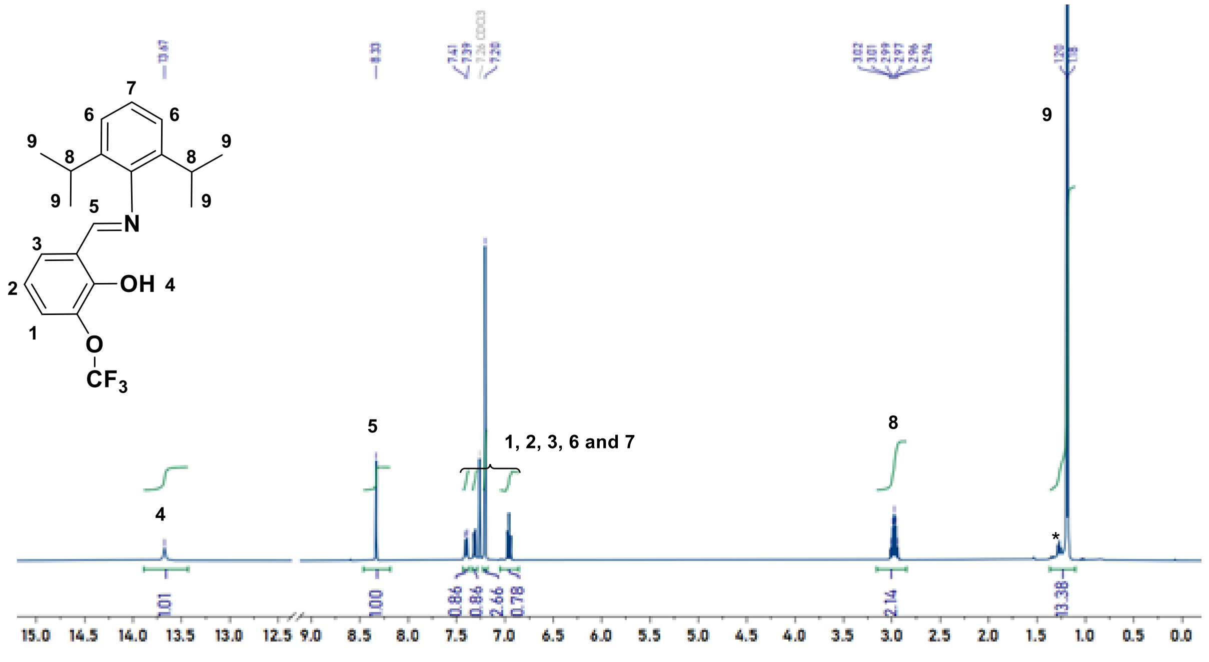

Figure S1: ^{1}H NMR spectrum of catalyst HL_1 (400 MHz, $CDCl_3$). * = Residual 2,6-diisopropylaniline after recrystallisation and washing with EtOH, <1%.

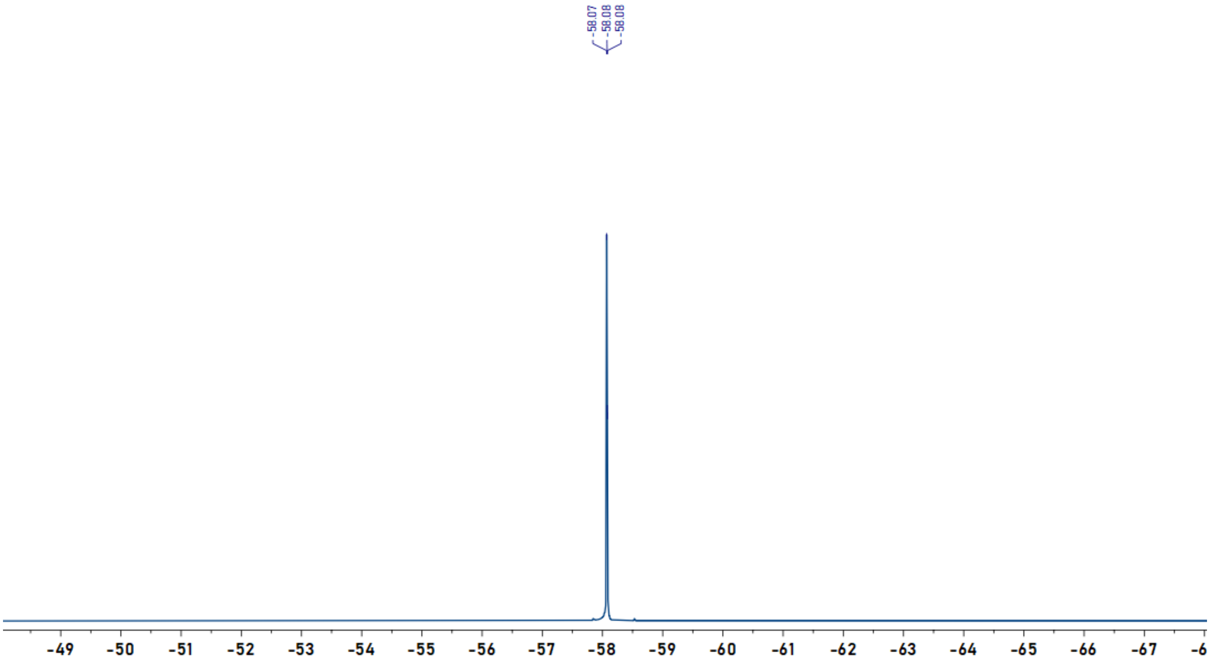

Figure S2: ^{19}F NMR spectrum of HL_1 (376 MHz, $CDCl_3$).

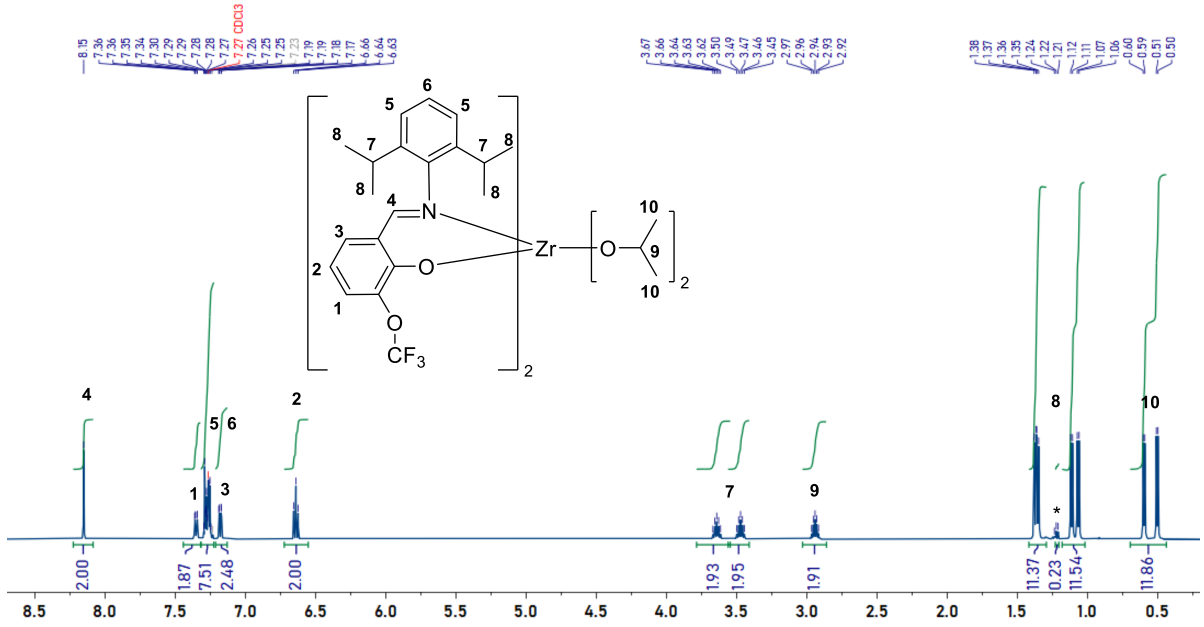

Figure S3: ^{1}H NMR spectrum of catalyst **1**, (400 MHz, $CDCl_3$). * = ^{i}PrOH produced by low levels of protonolysis ≈1%.

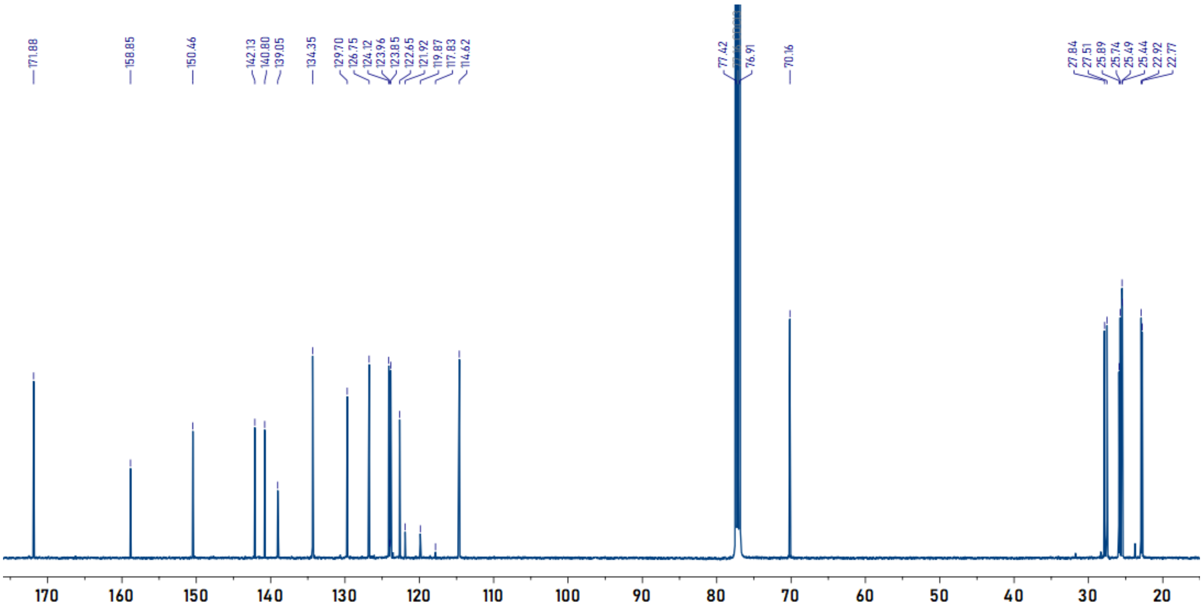

Figure S4: ^{13}C NMR spectrum of catalyst **1** (126 MHz, $CDCl_3$).

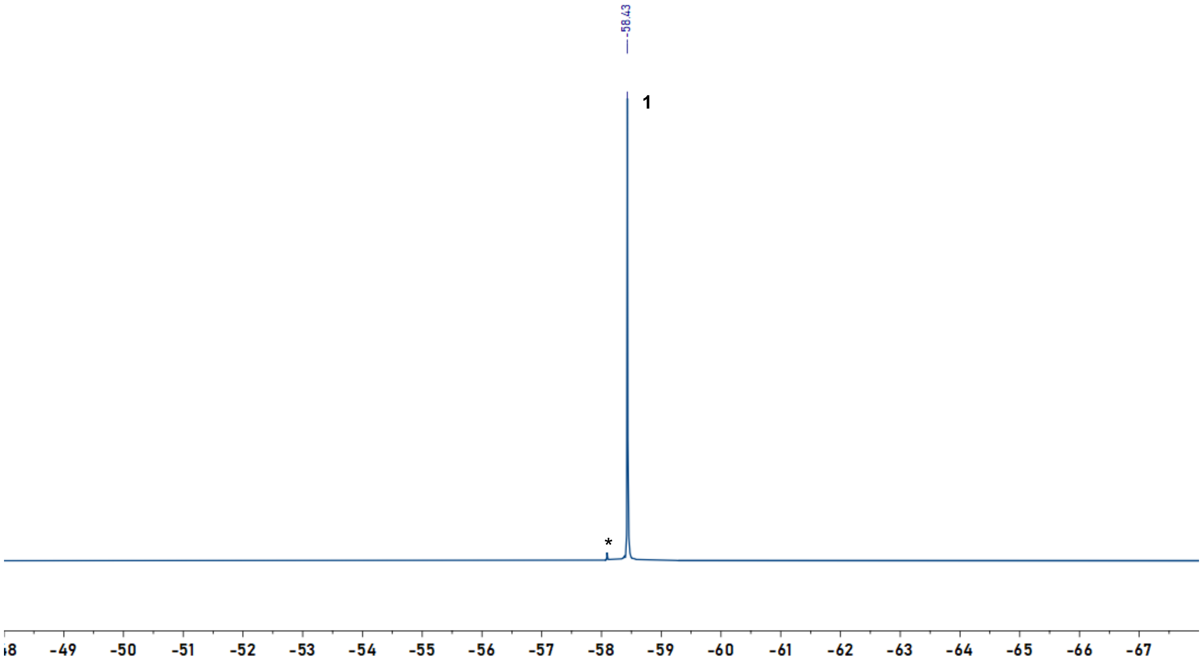

Figure S5: ^{19}F NMR spectrum of catalyst **1**, (376 MHz, $CDCl_3$). * = unknown impurity ≈1% intensity of main peak.

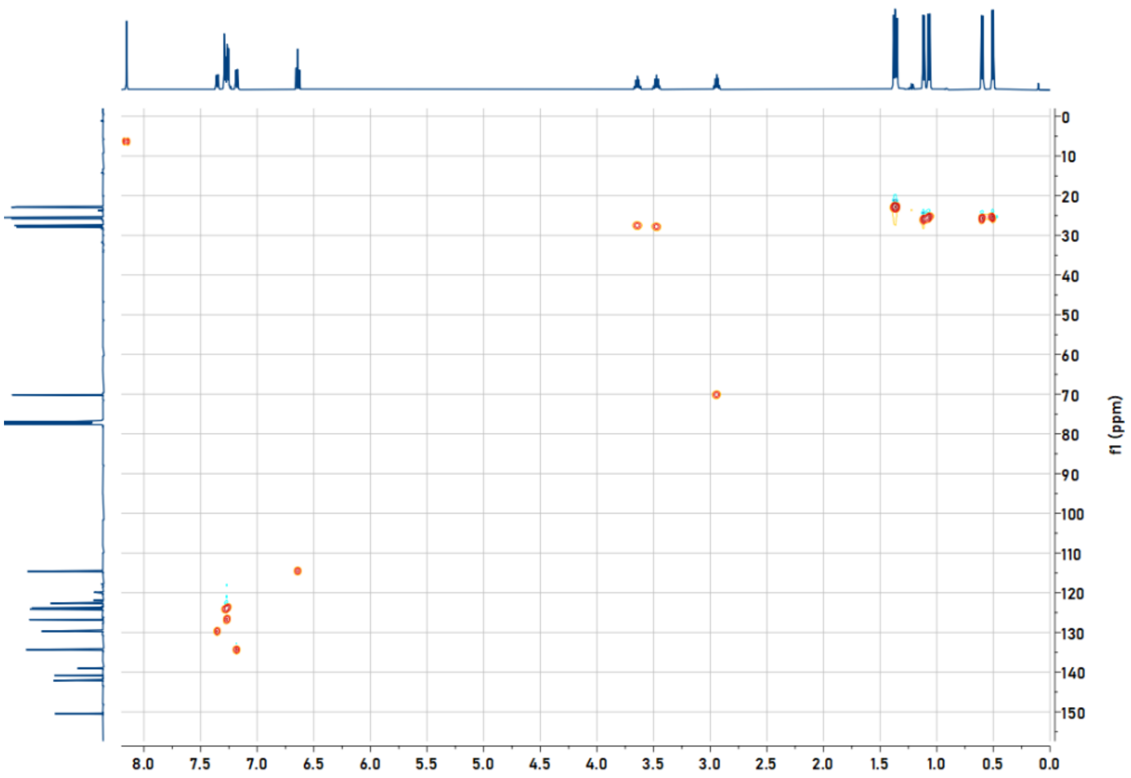

Figure S6: ^{1}H-^{13}C HSQC NMR spectrum of catalyst **1**.

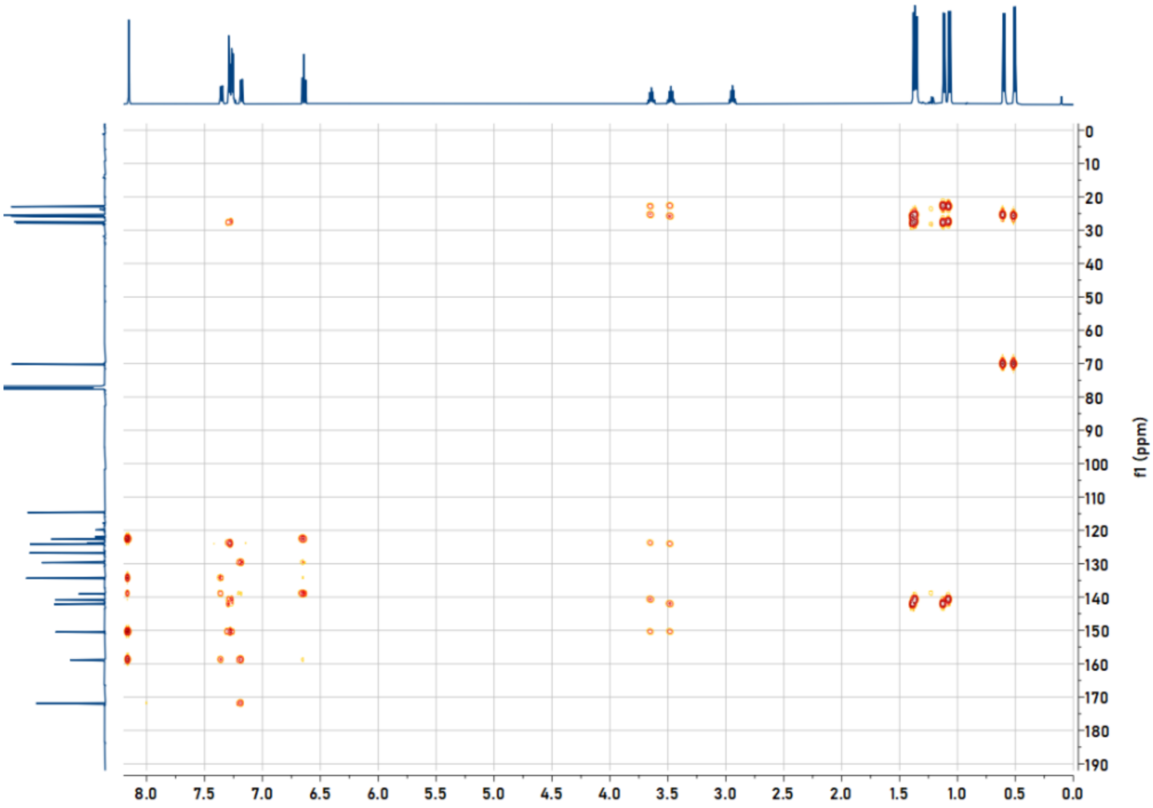

Figure S7: ^{1}H-^{13}C HMBC NMR spectrum of catalyst **1**.

2.0 Ring-opening Copolymerization of Phthalic Anhydride, Oxiranes, Oxetanes and Tetrahydrofurans

2.1 Polymerization techniques

2.1.1 Sealed vial method:

In a glovebox, the catalyst was weighed into a vial, then dissolved in the required volumes of (co)-monomer to reach the required solution concentration (10 mM in 1 mL). Phthalic anhydride (74 mg, 0.5 mmol) was added to the reaction mixture, the vial sealed, removed from the glovebox and heated to the stipulated temperature (50 °C for catalysis and some control reactions or 80 °C for some control reactions). After the desired time, samples were cooled to 0 °C, taken into the glovebox for removal of aliquots (20 µL) or exposed to air and evaporated to dryness. The crude polymer was characterized as approximately a 10 mg/mL THF solution for GPC and a 10 mg/mL $CDCl_3$ solution for ^1H NMR spectroscopy. The pure polymer was obtained by precipitation in DCM/MeOH or DCM/Pentane and drying under vacuum at 40 °C, overnight.

2.1.2 ReactIR Spectroscopy method:

In a glovebox, the desired quantity of catalyst and phthalic anhydride were weighed into an ampoule and removed from the glovebox. The ampoule was submerged in an oil bath to 50 °C and cycled three times with vacuum and N_2 and opened to a Schleck line. The ReactIR probe was warmed with a heatgun to remove residual moisture and purged, 10 times using a flow of N_2 from the ampoule, before being set into the vessel and beginning background scans. Finally, butylene oxide (1 mL) was injected into the reaction mixture, and scans were taken every 120 seconds thereafter. After the reaction was completed, the sample was removed from the oil bath and exposed to air. The crude polymer was characterized as approximately a 10 mg/mL THF solution for GPC and a 10 mg/mL $CDCl_3$ solution for ^1H NMR spectroscopy.

2.1.3 NMR Spectroscopy method:

In a glovebox, the catalyst was weighed into a vial, then dissolved in the required volumes of butylene oxide and tetrahydrofuran stock solutions to reach the required molarity of catalyst (10 mM, total volume = 0.7 mL after mixing, measured with a Hamiltonian syringe). Phthalic anhydride (50 mM) was added to the reaction mixture, the vial sealed and shaken until fully dissolved. The reaction mixture was transferred to a Young Taps NMR tube containing a sealed capillary of mesitylene in C_6D_6 (0.29 M). The NMR tube was submitted to the instrument preheated to 50°C and analysed by data acquisition every 10 minutes (8 scans, D_1 = 60 s).

Note: The stated D_1 was necessary to resolve and fit epoxide co-solvent integrations, shorter times for D_1 resulted in inferior data fit. Additionally, the epoxide resonances are better resolved when a wide capillary (containing the internal standard, mesitylene) is used.

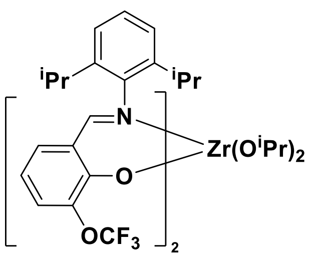

1

(This work)

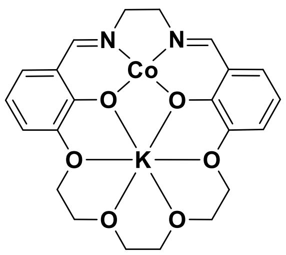

CoK[2]

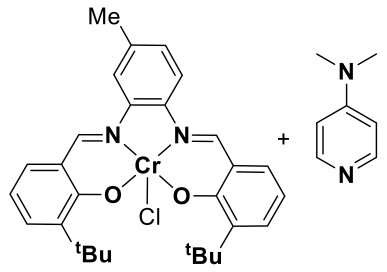

CrSalen: DMAP[3]

Chart S1: Structures of catalysts referenced in Table 1.[2,3]

2.2 NMR Spectroscopy Data Handling Procedures, relevant to entries in Table 1.

As described, monitoring of the reaction was critical to understanding polymer composition. Therefore, an example ^{1}H NMR spectra, obtained after 1.5 h, from a dried reaction aliquot is presented, as well the related purified product, Figure S8-9. Also, the methods used for calculating the degrees of polymerisation for each monomer are described:

The conversion of PA was measured against it's polymeryl species (PA = 7.98 ppm *vs* P(PA) 7.59 ppm, in pink). As well a useful indicator of how the reaction was progressing, the percentage of polymerised PA was vital for calculating the DP of other monomers:

$$PA\ conversion\ (\%) = \frac{Total\ integral\ of\ P(PA)}{Total\ integral\ of\ PA\ and\ P(PA)} \times 100\%$$

Next, to calculate the sum of polymerised butylene oxide, the P(PA) integral was set to 4, (the number of protons for 1 equivalent of P(PA)) and integrated against the P(BO) methyl peak (≈ 0.9 ppm). Division of this number by 3, gives relative equivalents of P(BO) in the polymer.

$$Equivalents\ of\ P(BO) = \frac{Integral\ of\ P(BO)\ methyl}{3}$$

From this, the DP for PA and BO can be calculated from the starting ratios of reagents ([Cat]:[PA]:[BO]:[THF]) where [cat] = 1.

$$DP\ for\ PA = PA\ Conversion\ (\%) \times [PA]_0$$

$$DP\ for\ BO = DP\ for\ PA \times Equivalents\ of\ P(BO)$$

Finally, in reactions containing THF, the equivalents of P(THF) and the DP for THF can be calculated. All the shifts of P(THF) overlap with P(BO) resonances, so DP cannot be calculated directly. Rather it is determined, after the DP P(BO) has been determined (see above). Once the number of P(BO) molecules are known, the value is multiplied by 8 (total protons in each P(BO) repeat unit). This value is then subtracted from the total integral for the signal for the combined P(BO) and P(THF) resonances (setting the integral for P(PA) = 4) to give the total integral of P(THF). The equivalents of THF is obtained by division of this number by 8 (total protons in each P(THF) repeat). To close, the DP of THF is then calculated using the DP for PA.

$$Total\ integral\ of\ P(THF) = [Total\ integrals\ of\ P(THF) + P(BO)] - [8\ \times Equivalents\ of\ P(BO)]$$

$$Equivalents\ of\ P(THF) = \frac{Total\ integral\ of\ P(THF)}{8}$$

$$DP\ for\ THF = DP\ for\ PA \times Equivalents\ of\ P(THF)$$

2.2.1 Crude NMR Spectra for P1 (PA-BO-BO)

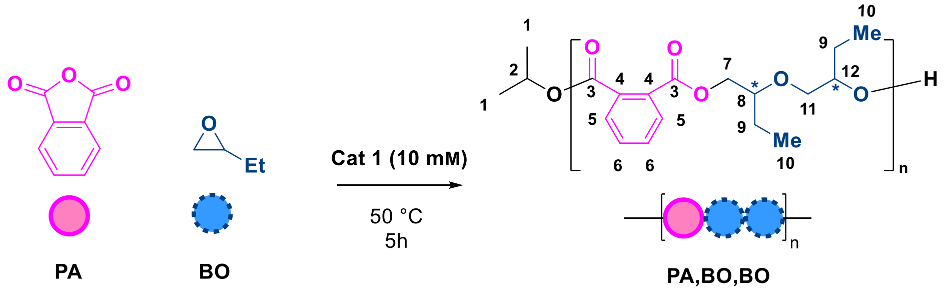

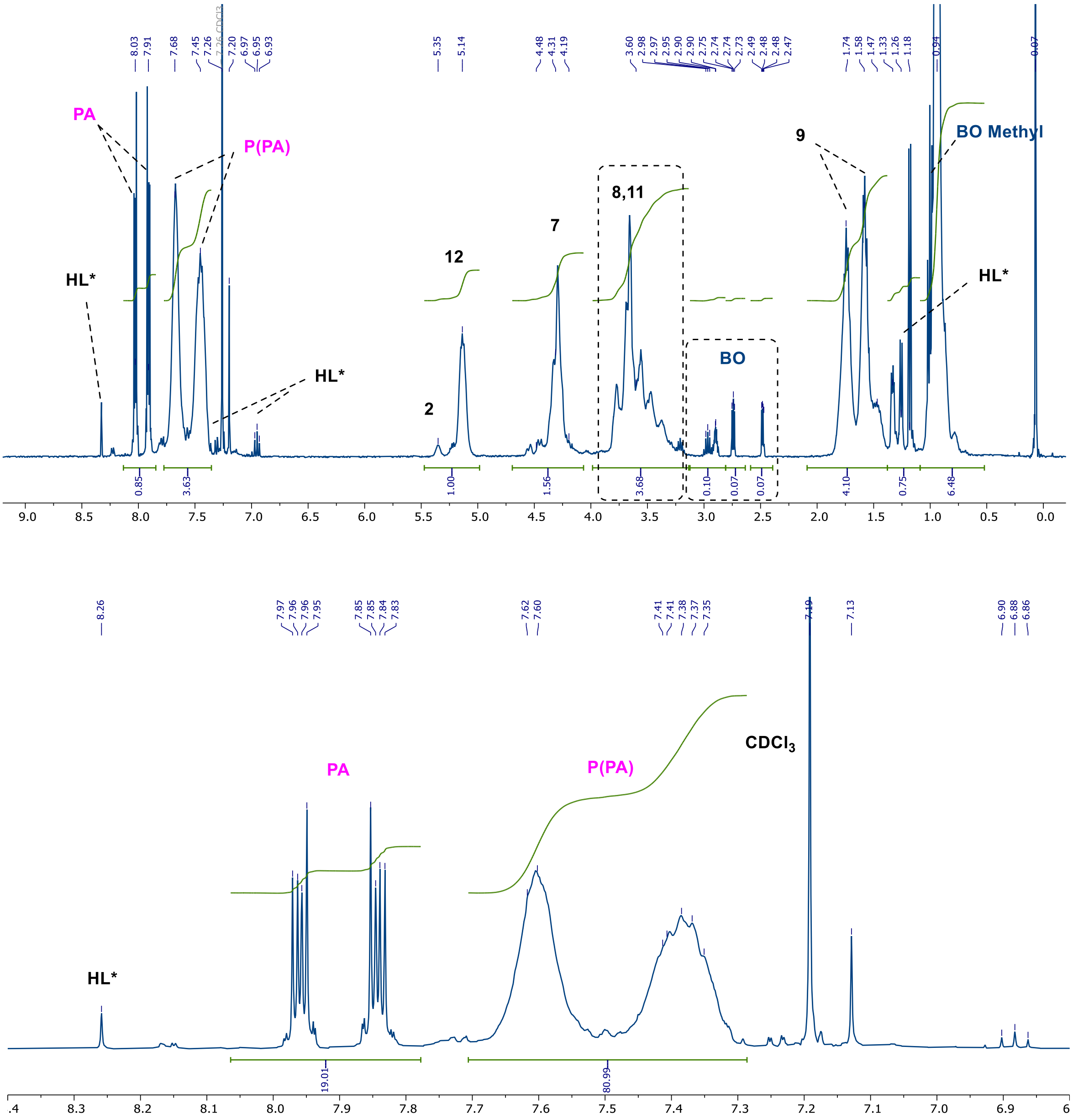

Figure S8: Example crude ^{1}H NMR spectrum produced from a dried aliquot of the reaction of [Cat]:[PA]:[BO]:[THF] = 1: 50: 1150: 0 after 1.5 h (top) and magnification of aromatic region used to calculate the conversion of PA (bottom) (400 MHz, $CDCl_3$). *Free ligand observed after the hydrolysis of **1** upon exposure to air and $CDCl_3$.

2.2.2 NMR Spectra for P1, P(PA-BO-BO) (Table 1)

PA,BO,BO

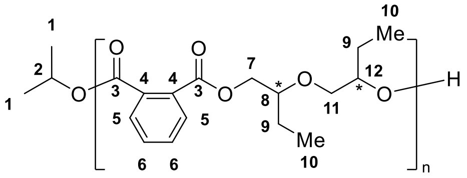

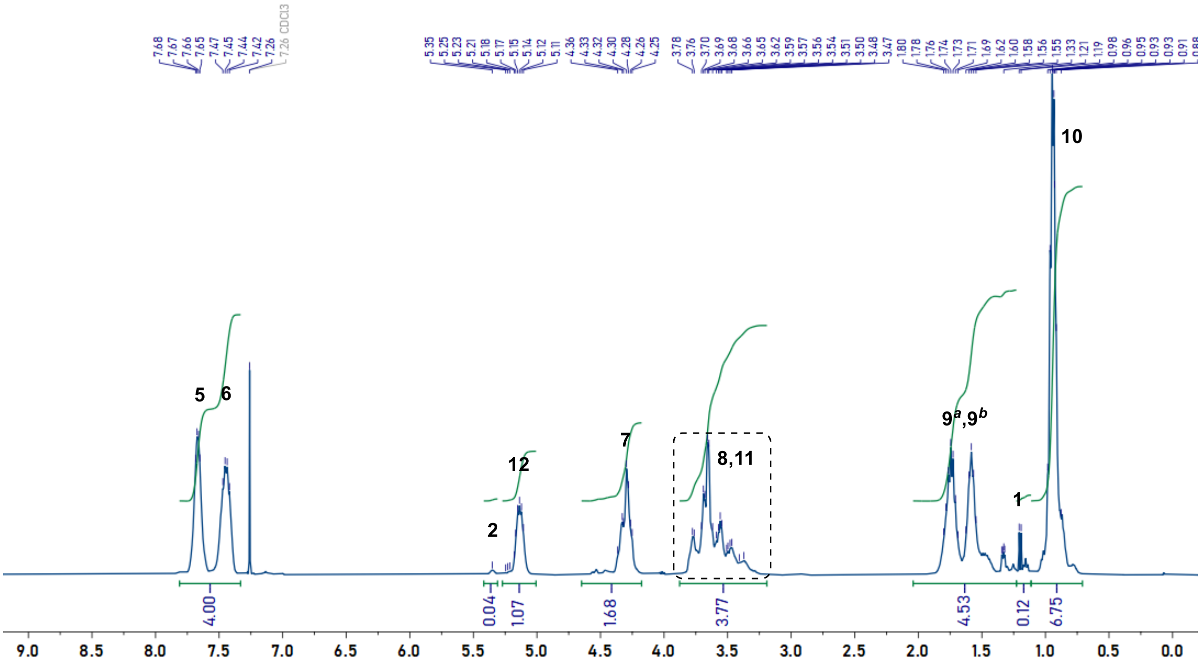

Figure S9: ^{1}H NMR spectrum from the reaction of [Cat]:[PA]:[BO]:[THF] = 1: 50: 1150: 0, after work-up in MeOH/DCM. Spectrum corresponds to Table 1, **P1** (400 MHz, $CDCl_3$).

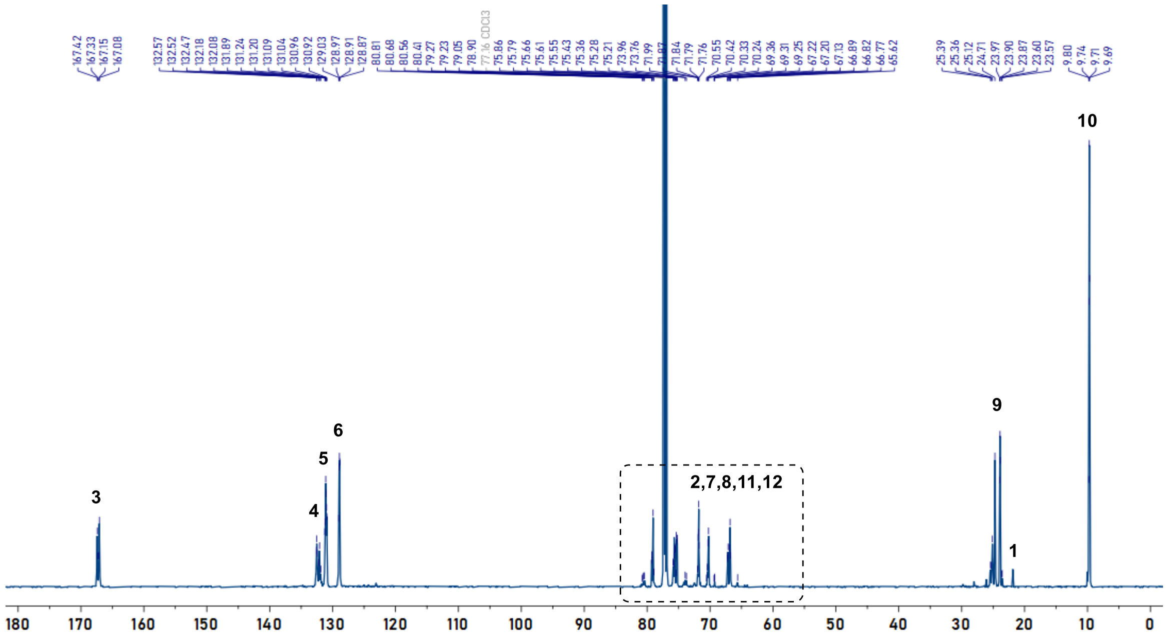

Figure S10: ^{13}C NMR spectrum from the reaction of [Cat]:[PA]:[BO]:[THF] = 1: 50: 1150: 0 after work-up in MeOH/DCM from Table 1, **P1** (128 MHz, $CDCl_3$).

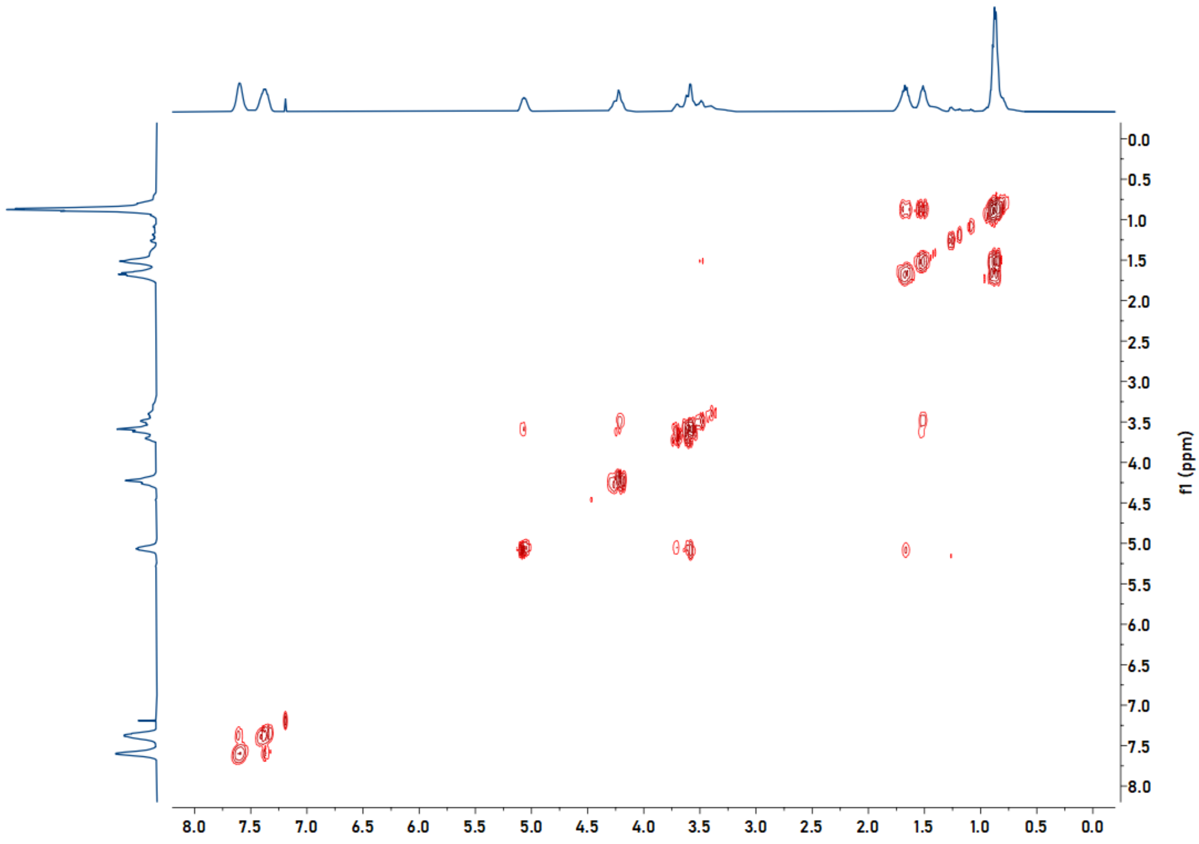

Figure S11: ^{1}H COSY NMR spectrum from the reaction of [Cat]:[PA]:[BO]:[THF] = 1: 50: 1150:0 after work-up in MeOH/DCM from Table 1, **P1** (400 MHz, $CDCl_3$).

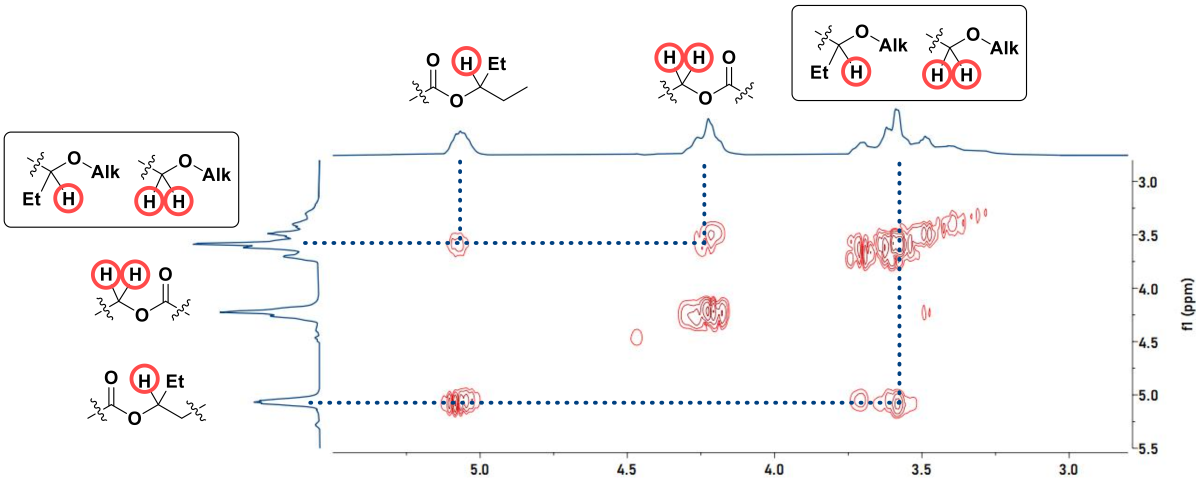

Figure S12: Magnified ^{1}H COSY NMR spectrum from the reaction of [Cat]:[PA]:[BO]:[THF] = 1: 50: 1150: 0 after work-up in MeOH/DCM from Table 1, **P1** (400 MHz, $CDCl_3$).

2.2.2 NMR Spectra for P4, P(PA-BO-BO)-(PA-BO-THF) (Table 1)

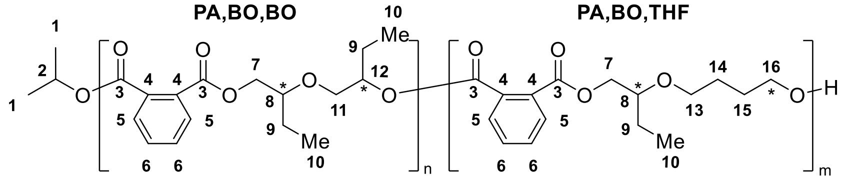

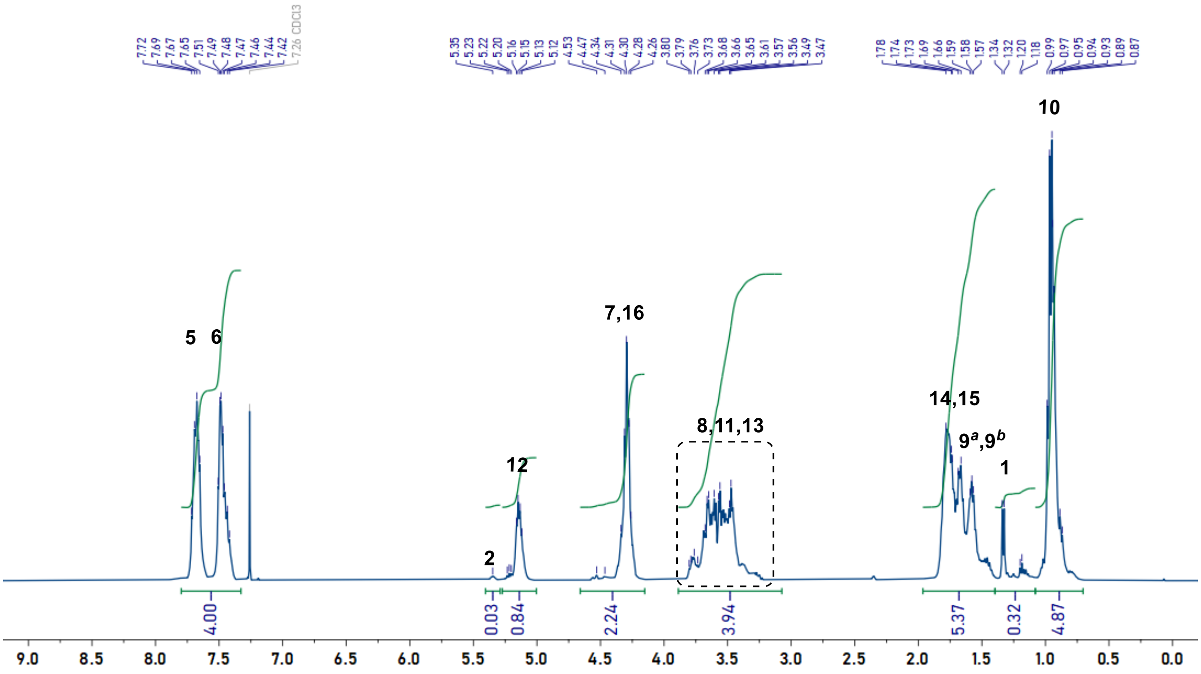

Figure S13: ^{1}H NMR spectrum from the reaction of [Cat]:[PA]:[BO]:[THF] = 1: 50: 288: 924 after work-up in MeOH/DCM from Table 1, **P4** (400 MHz, $CDCl_3$).

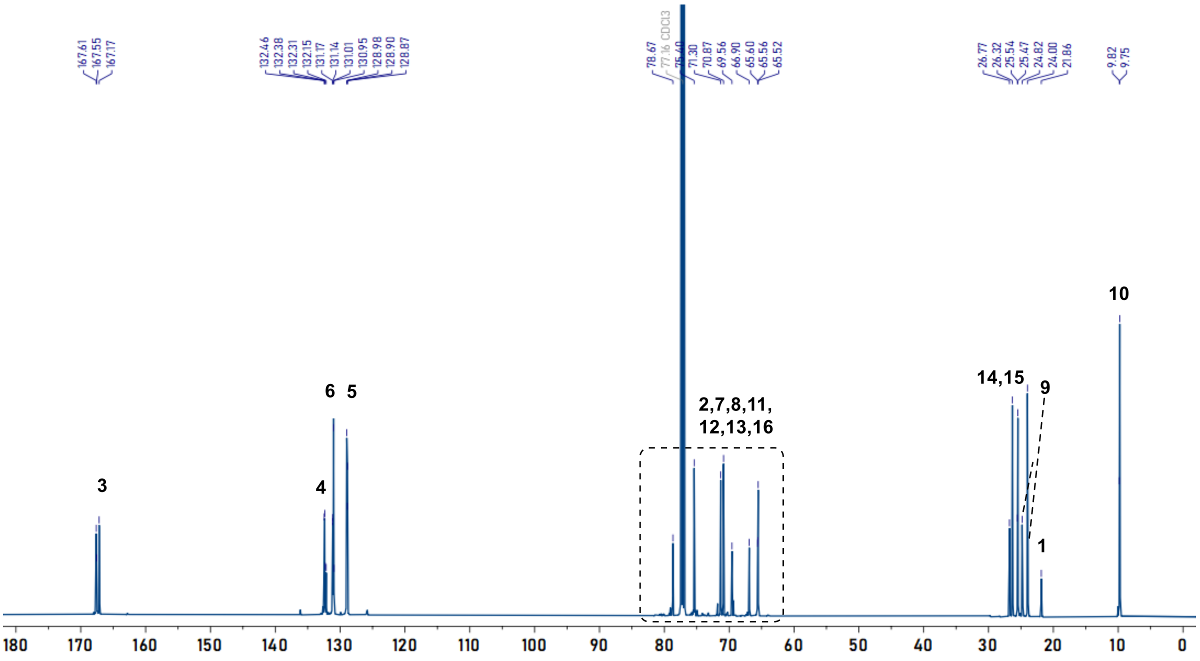

Figure S14: ^{13}C NMR spectrum from the reaction [Cat]:[PA]:[BO]:[THF] = 1: 50: 288: 924 after work-up in MeOH/DCM from Table 1, **P4** (128 MHz, $CDCl_3$).

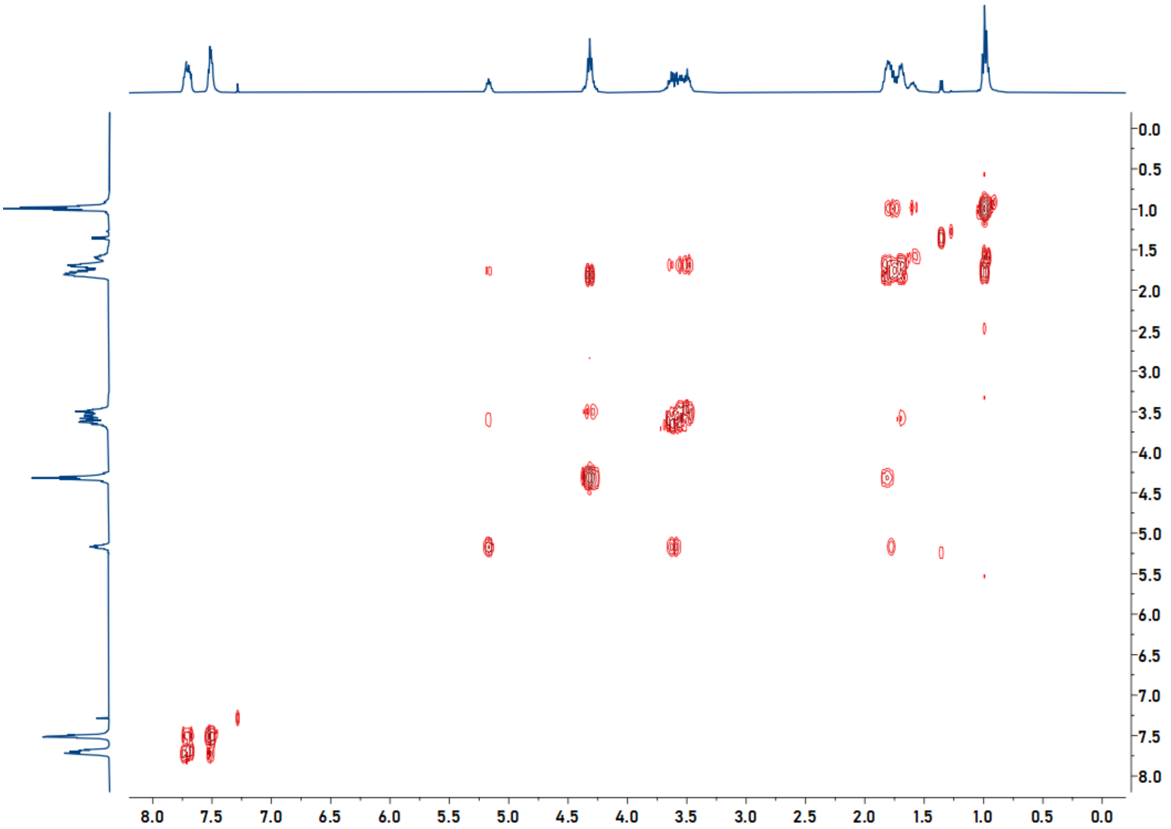

Figure S15: ^{1}H COSY NMR spectrum from the reaction of [Cat]:[PA]:[BO]:[THF] = 1: 50: 288: 924 after work-up in MeOH/DCM from Table 1, **P4** (400 MHz, $CDCl_3$).

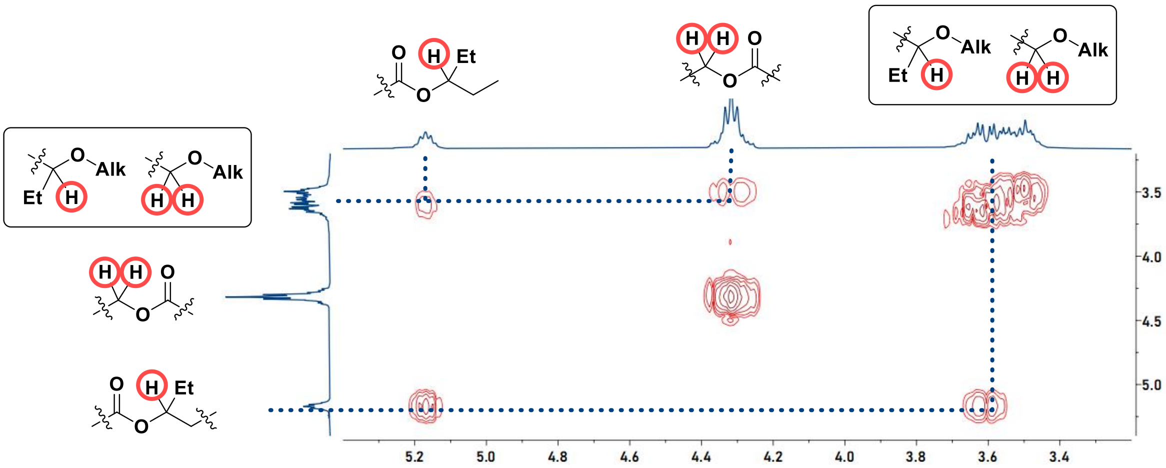

Figure S16: Enlarged ^{1}H COSY NMR spectrum from the reaction of [Cat]:[PA]:[BO]:[THF] = 1: 50: 288: 924 after work-up in MeOH/DCM from Table 1, **P4** (400 MHz, $CDCl_3$).

2.2.3 NMR Spectra for P7, P(PA-BO) (Table 1)

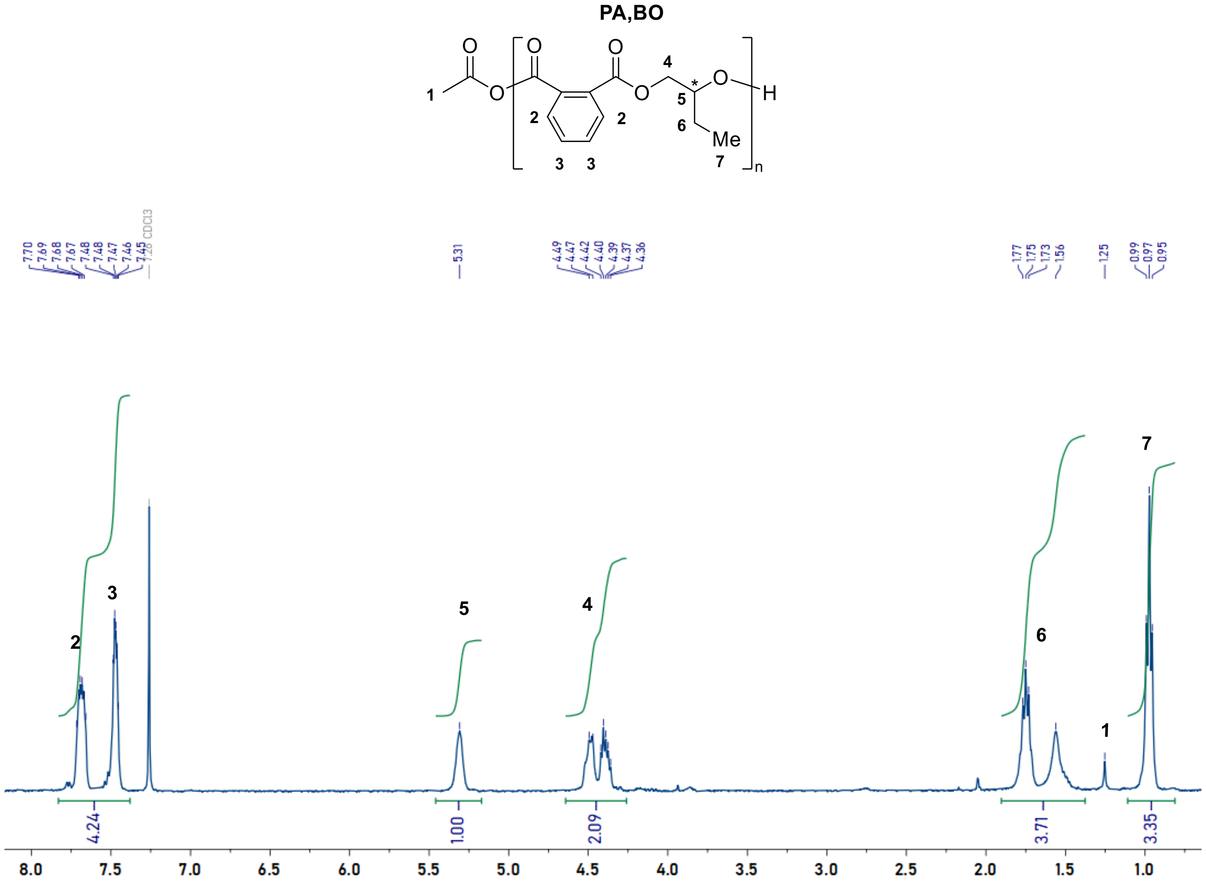

Figure S17: ^{1}H NMR spectrum from the reaction of [Cat]:[PA]:[BO] = 1: 100: 800 after work-up in MeOH/DCM from Table 1, **P7** (400 MHz, $CDCl_3$), in accordance with literature.[3]

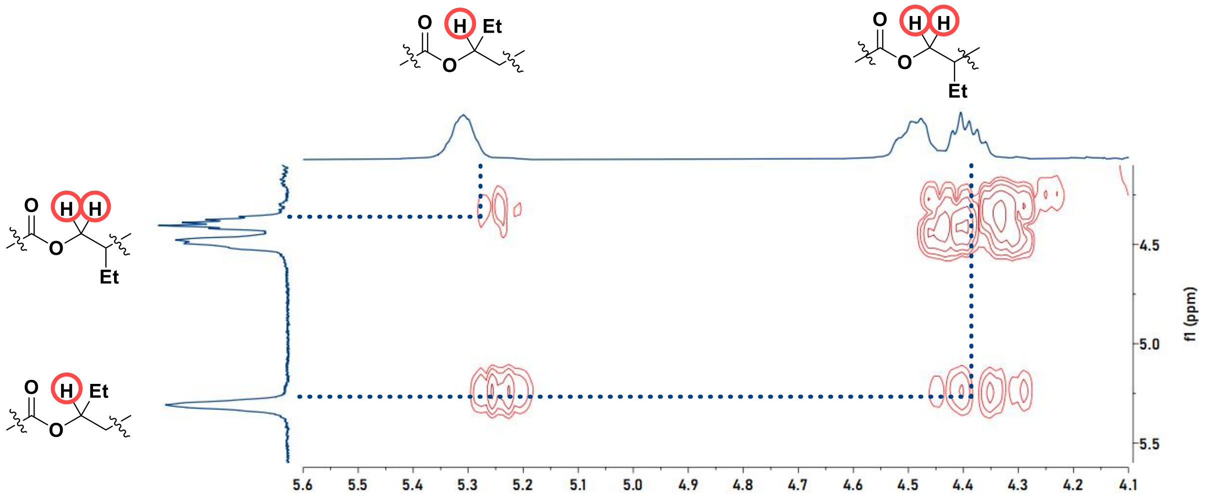

Figure S18: ^{1}H COSY NMR spectrum from the reaction of [Cat]:[PA]:[BO] = 1: 100: 800 after work-up in MeOH/DCM from Table 1, **P7** (400 MHz, $CDCl_3$). This shows the correlation in P(PA-*alt*-BO) between methine and methylene ester protons absent in the poly(ester-*alt*-ethers).

Table S1: Ring-opening copolymerization (ROCOP) of phthalic anhydride (PA), butylene oxide (BO) with **1** or **CoK**

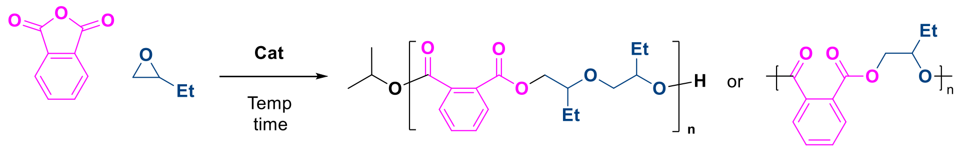

Entry #	Cat	Starting Monomer Stoichiometry [Cat]:[PA]:[BO]	Degrees of Polymerization (DP): [PA]:[BO][cd]	Polymer Selectivity for BO[%][d]	M_n (Đ) [kg mol^{-1}][e]	M_n (Theo)[f]	TOF (h^{-1})[g]
1 (**P2**-Table 1 **)**	**1**[a]	1: 50: 1150: 0	50: 110	69	8.2 (1.26)	7.7	42
2 (**P7**-Table 1 **)**	**CoK**[b]	1: 100: 800: 0	60: 60	50	6.7 (1.25)	7.1	60
3	**CoK**[a]	1: 50: 1150: 0	50: 50	50	6.2 (1.17)	5.5	44

[a]ROCOP Conditions: [**Cat**] = 10 mM, [PA] = 0.5 M, BO = 0 to 1 mL, THF = 0 to 1 mL, total volume of THF + BO = 1 mL, 50 °C, 5 h. [b]ROCOP Conditions: [**CoK**] = 14 mM, [PA] = 1.43 M, [BO] = 16.9 M, 1 mL, 60 °C, 1 h, (Chart S1; ESI section 2.2.3). [c]DP of PA measured by integration of the aromatic resonances of PA (7.98 ppm) and P(PA) (7.59 ppm) in the ^{1}H NMR spectra of crude polymers (Fig. S8). [d]Determined by integration of the ^{1}H NMR spectra of purified polymer against P(PA) (Figures S9 and S18). [e]Determined by gel permeation chromatography (GPC), using THF as the eluent, and calibrated using narrow MW polystyrene standards (Figs. S19). [f]Theoretical M_n are calculated from the monomer conversion data and assume both iso-propoxides initiate. [g]Turn over frequency (TOF) = (number of moles of monomer converted/number of moles of catalyst)/hour measured at 1 h.

2.3 GPC Data for P1-4 (Table 1)

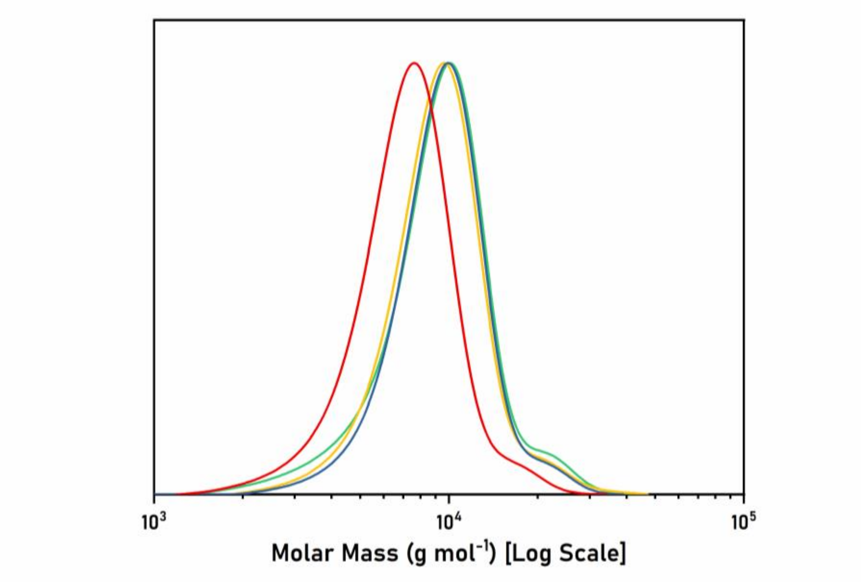

Figure S19: GPC chromatograms for the polymers described in Table 1: **P1** (♣) [Cat]:[PA]:[BO]:[THF] = 1: 50: 1150: 0, M_n = 8.2 kg mol^{-1}, Đ = 1.26, time = 5 h; **P2** (♦) [Cat]:[PA]:[BO]:[THF] = 1: 50: 863: 308, M_n = 8.4 kg mol^{-1}, Đ = 1.17, time = 5 h; **P3** (♥) [Cat]:[PA]:[BO]:[THF] = 1: 50: 575: 616, M_n = 8.7 kg mol^{-1}, Đ = 1.15, time = 5 h; **P4** (♠) [Cat]:[PA]:[BO]:[THF] = 1: 50: 288: 924, M_n = 6.5 kg mol^{-1}, Đ = 1.18, time = 5 h.

In some cases, the GPC data show a low intensity high molar mass shoulder (<2% overall peak intensity), which is attributed to chains initiated from 1,2-butanediol. The diol forms by hydrolysis of BO. Other authors have

written on the propensity for epoxide hydrolysis and the formation of bimodal molar mass distributions in such cases.[4]

2.4 ^{1}H NMR Spectra of P1 reaction aliquots used to determine polymer composition versus time

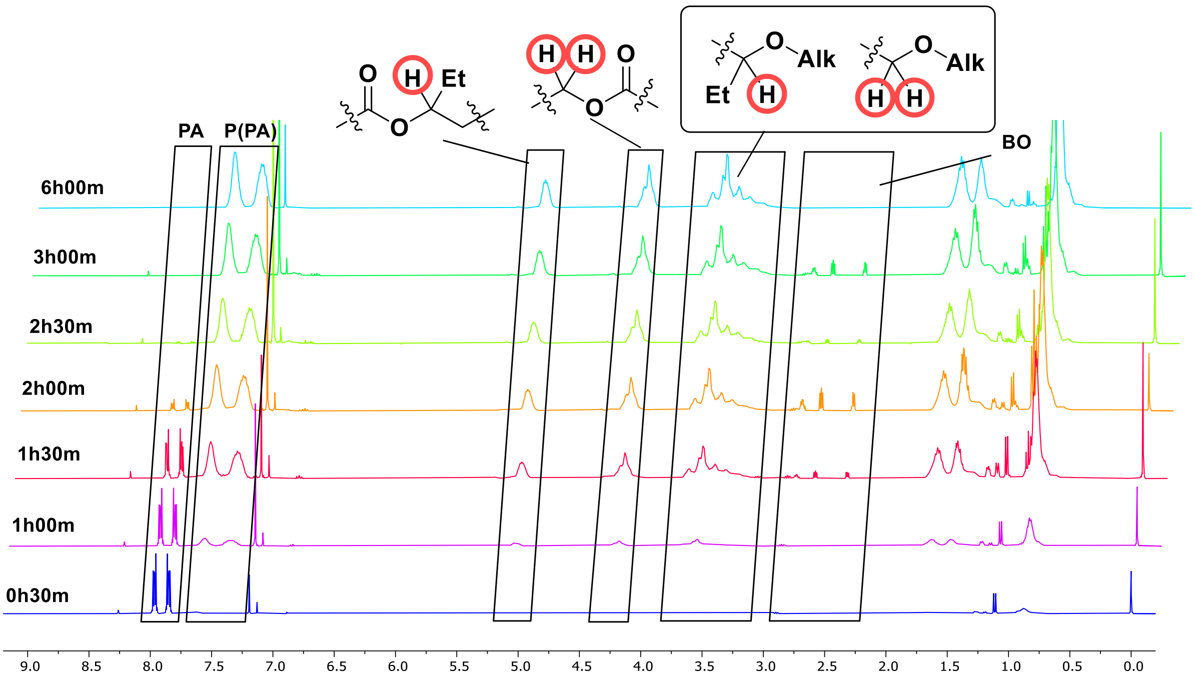

Figure S20: ^{1}H NMR spectra produced from 20 µL aliquots, from the reaction of [Cat]:[PA]:[BO]:[THF] = 1: 50: 1150: 0, after drying. Data corresponds to Table 1, **P1** and Figure 3A (400 MHz, $CDCl_3$).

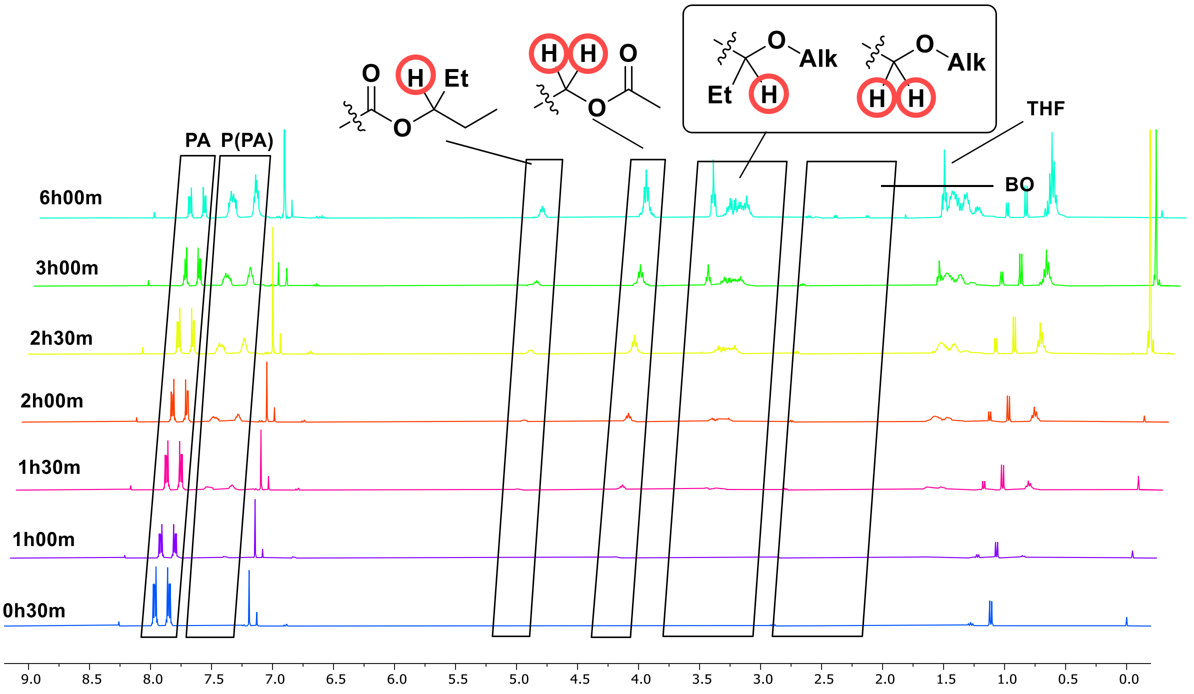

Figure S21: ^{1}H NMR spectra produced from 20 µL aliquots, from the reaction of [Cat]:[PA]:[BO]:[THF] = 1: 50: 288: 924, after drying. Data correspond to Table 1, **P4** and Figure 3B (400 MHz, $CDCl_3$).

2.5 GPC Data Showing changes to molar mass over time, during polymer formation

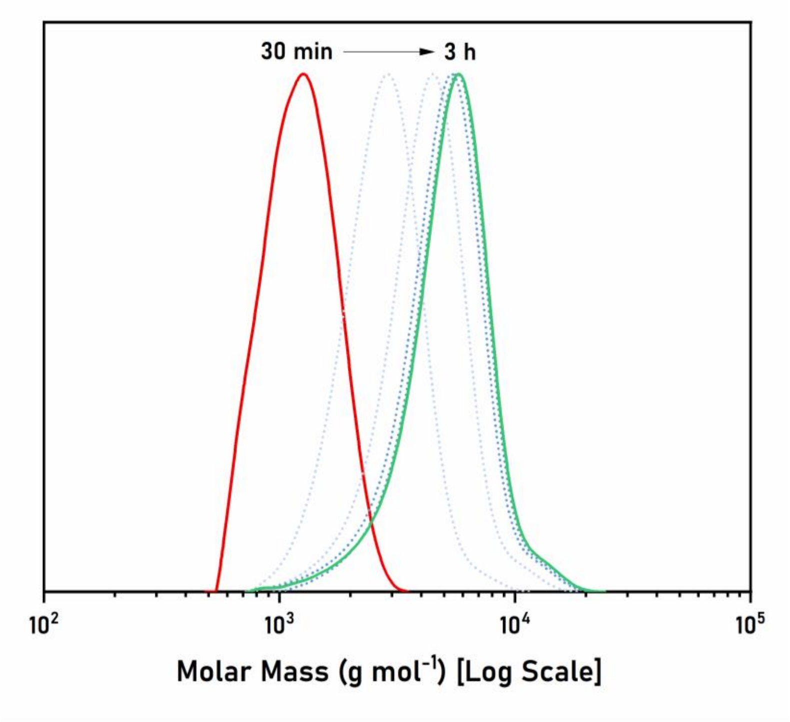

Figure S22: GPC trace produced from 20 µL aliquots, from the reaction of [Cat]:[PA]:[BO]:[THF] = 1: 50: 1150: 0. Data correspond to Table 1, **P1** and Figure 3A and 3C.

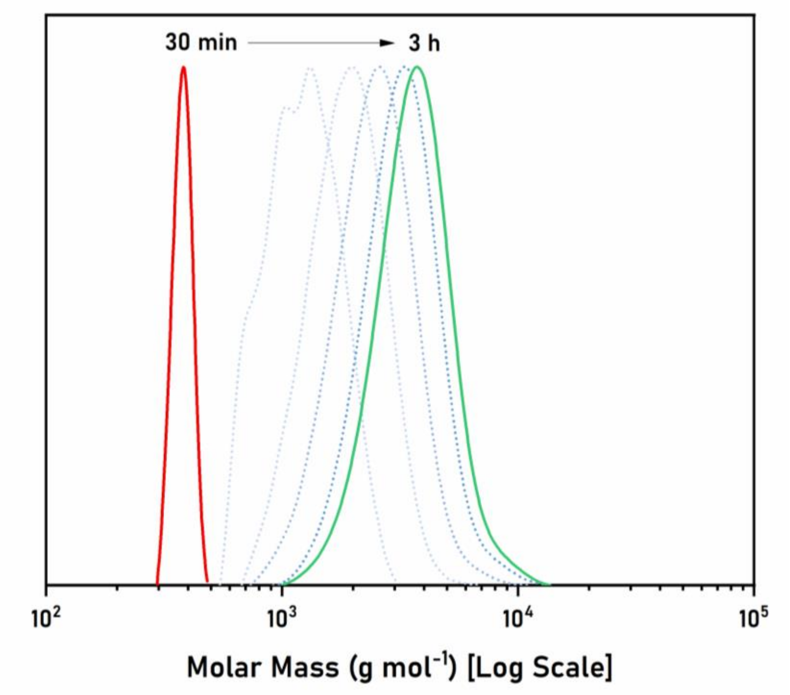

Figure S23: GPC trace produced from 20 µL aliquots, from the reaction of [Cat]:[PA]:[BO]:[THF] = 1: 50: 288: 924. Data correspond to Table 1, **P4** and Figure 3B.

2.6 Polymer Degradation Experiments

To a Schott bottle charged with **P1**, **P4** or poly(PA-*alt*-BO) (50 mg) was added an aqueous solution of KOH (5 M, 50 mL). The suspension was sealed, heated to 70 °C and stirred using a magnetic stirrer bar, at 1000 rpm. Aliquots of the suspension for GPC analysis were obtained by cooling the mixture to RT, cutting a piece from the coagulated polymer (approximately 2 mg), washing with water and dissolving in THF. After 7 days, the reaction was quenched with HCl until pH 7. In the case of **P1** and **P4** a completely homogeneous solution was observed.

LCMS analysis: To a 0.5 mL aliquot of degraded **P1** was added MeOH (0.5 mL) and the solution was submitted for LC-MS analysis. Note: Analysis was run on the crude sample in order to isolate as many fragments as possible thus analytes containing K^+, H_2O and MeOH from the crude mixture are observed as well as Na^+ from the instrument. These have been documented in **Table S1A-B**.

2.6.1 LCMS Data from the Degradation of P1 (Table 1)

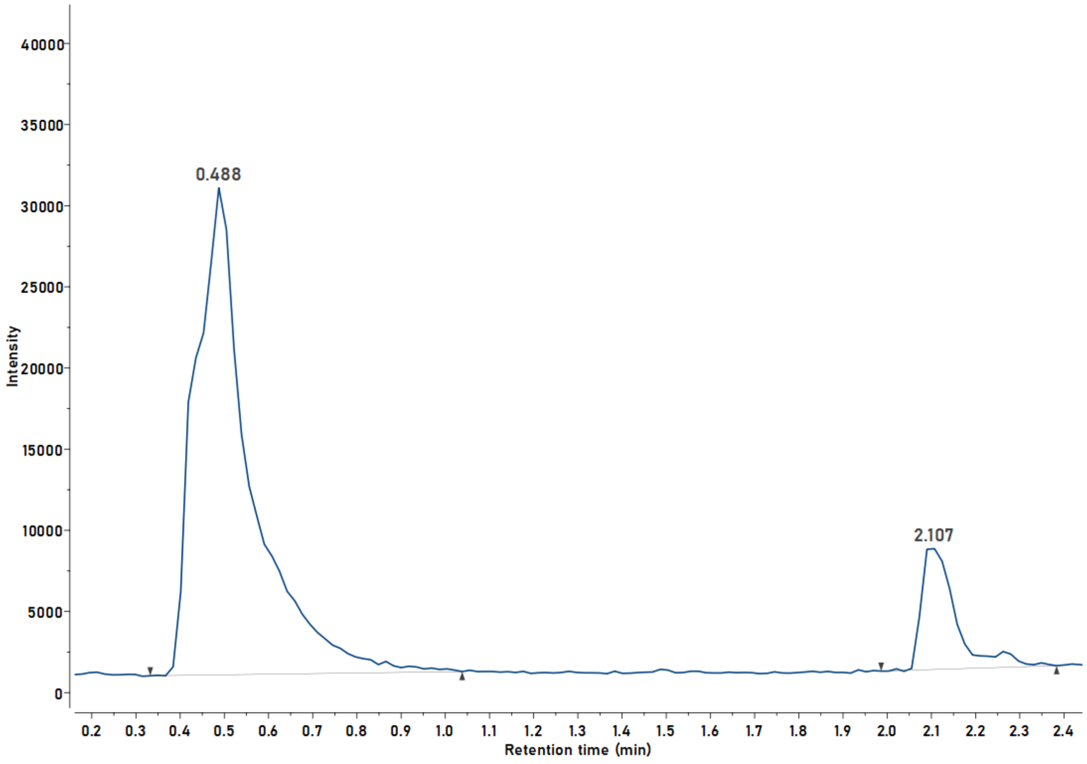

Figure S24: LC-MS Chromatogram of the degraded, **P1** (TOF MS ES- TIC). Degradation conditions: **P1** (50 mg) in KOH (5 M, 50 mL), 70 °C, 7 days.

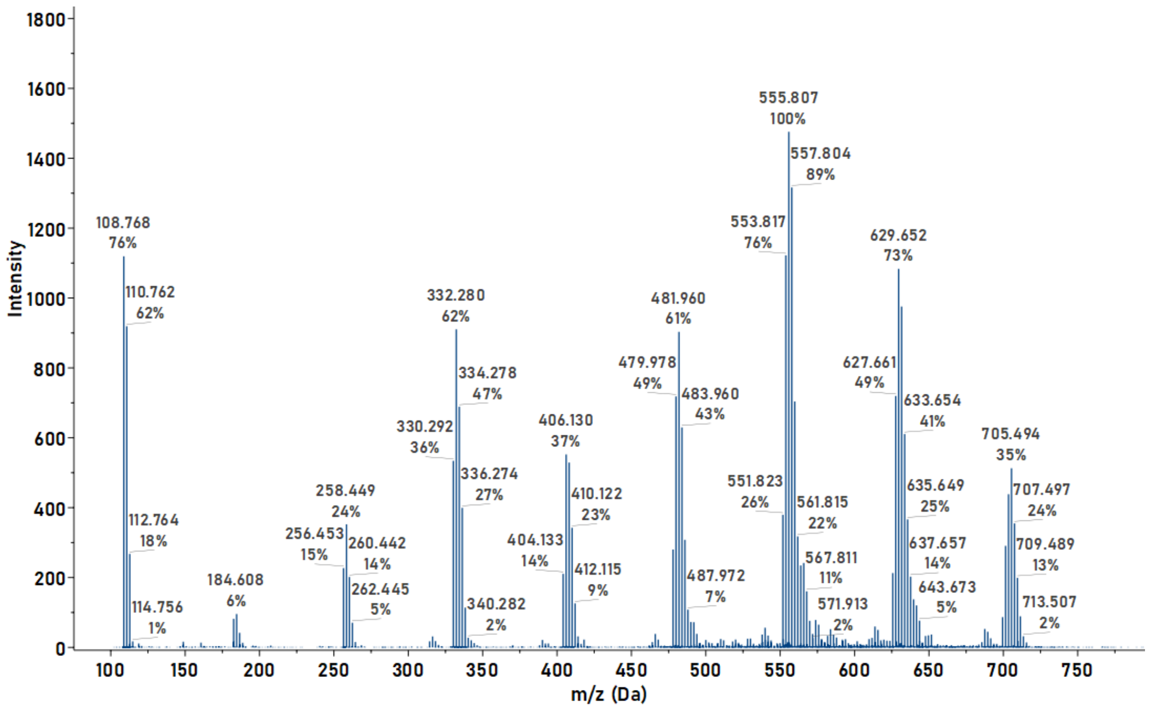

Figure S25: Negative ESI-MS spectrum of the products of **P1** degradation at 0.5 minutes. Degradation conditions: **P1** (50 mg) in KOH (5 M, 50 mL), 70 °C, 7 days.

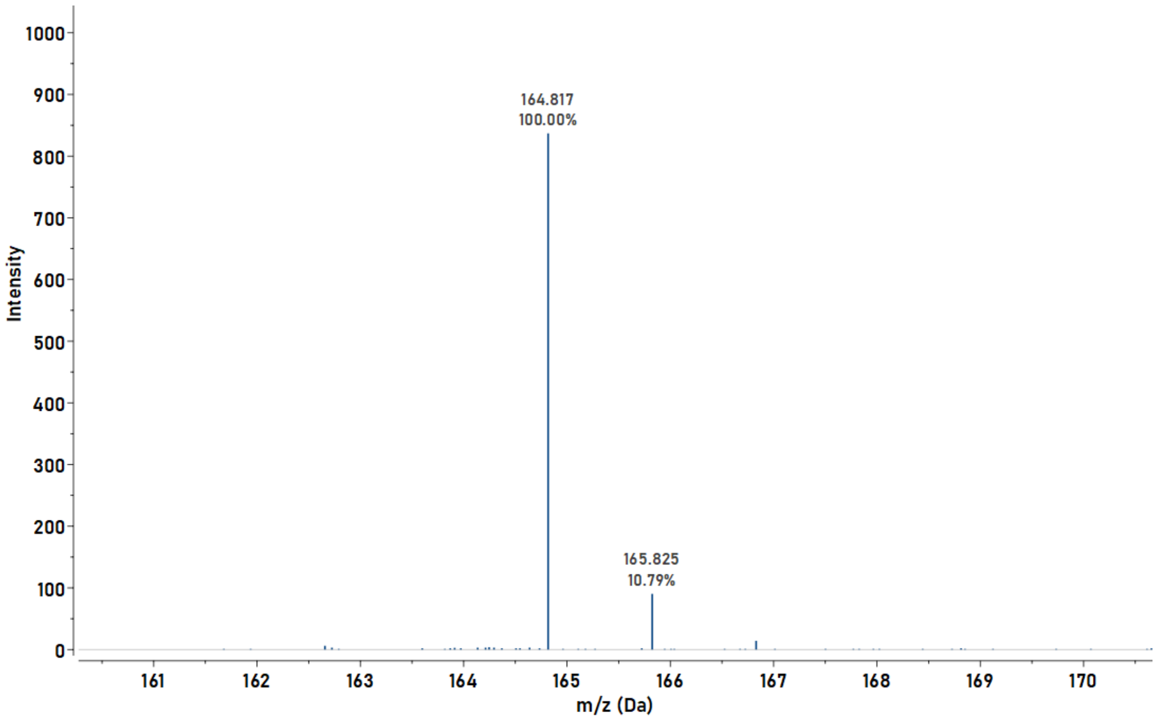

Figure S26: Negative ESI-MS spectrum of the degradation products from **P1**,at 2.1 minutes. Degradation conditions: **P1** (50 mg) in KOH (5 M, 50 mL), 70 °C, 7 days.

Table S2A: Complete Data and Assignments of the analytes observed from the negative ESI-MS spectrum for the degradation products of **P1**. Degradation conditions: **P1** (50 mg) in KOH (5 M, 50 mL), 70 °C, 7 days.

Product (M)	Analyte	Observed Mass	Expected Mass (relative intensity %)	Error (ppm)	Absolute Intensity	RT (min)
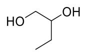 [HO-BO$_1$-H]	**[2M-3H+H$_2$O+Na]$^{2-}$**	**108.769**	**109.057 (100%)**	**-2640.821**	**1118**	**0.44**
	**[M-2H+Na]$^{1-}$**	**110.764**	**111.043 (100%)**	**-2509.845**	**919**	**0.44**
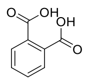 [HO-PA$_1$-H]	**[M-H]$^{1-}$**	**164.817**	**165.019 (100%)**	**-1225.917**	**837**	**2.09**
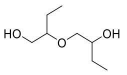 [HO-BO$_2$-H]	[M-2H+Na]$^{1-}$	182.611	183.100 (100%)	-2671.761	80	0.49
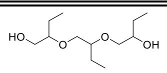 [HO-BO$_3$-H]	[M-2H+Na]$^{1-}$	256.453	255.158 (100%)	+5076.468	224	0.49
	[2M-2H+MeOH+H$_2$O]$^{2-}$	258.449	258.194 (100%)	+987.629	352	0.49
	[2M-2H+MeOH+Na]$^{2-}$	260.442	260.180 (100%)	+1006.995	201	0.49
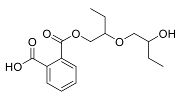 [HO-PA$_1$-BO$_2$-H]	[2M-3H+H$_2$O+Na]$^{2-}$	330.292	329.131 (100%)	+3528.690	534	0.49
	**[M-2H+Na]$^{1-}$**	**332.280**	**331.116 (100%)**<br>**332.120 (17%)**	**+3514.475**<br>**+481.754**	**909**	**0.49**
	[2M-2H+H$_2$O+MeOH]$^{2-}$	334.278	334.153 (100%)	+374.080137	686	0.49
	[2M-2H+MeOH+Na]^{2}	336.274	336.139 (100%)	+401.620	402	0.49
	[2M-3H+H$_2$O+K]$^{2-}$	338.273	337.118 (100%)<br>337.619 (35%)	+3427.291<br>+1937.095	113	0.49
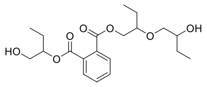 [HO-PA$_1$-BO$_3$-H]	[M-2H+Na]$^{1-}$	404.133	403.174 (100%)<br>404.178 (22%)	+2379.123<br>-111.337	208	0.49
	[2M-2H+MeOH+H$_2$O]$^{2-}$	406.130	406.210 (100%)	-196.942	551	0.49
	[2M-2H+MeOH+Na]^{2}	408.175	408.196 (100%)	-51.446	527	0.49
	[2M-3H+H$_2$O+K]$^{2-}$	410.122	409.175 (100%)<br>409.667 (43%)	+2314.168<br>+1110.658	343	0.49
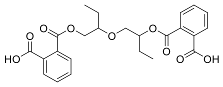 [HO-PA$_2$-BO$_2$-H]	[2M-3H+H$_2$O+Na]$^{2-}$	477.973	477.174 (100%)	+1674.442	281	0.49
	[M-2H+Na]$^{1-}$	479.978	479.113 (100%)	+1805.420	729	0.49
	**[2M-2H+MeOH+H$_2$O]$^{2-}$**	**481.960**	**482.169 (100%)**	**-433.458**	**904**	**0.49**
	[2M-3H+MeOH+Na]^{2}	483.960	484.155 (100%)	-402.764	633	0.49
	[2M-3H+H$_2$O+K]$^{2-}$	485.954	485.134 (100%)	+1690.256	309	0.49

Table S2B: Further Complete Data and Assignments of the analytes observed from the negative ESI-MS spectrum for the degradation products of **P1**. Degradation conditions: **P1** (50 mg) in KOH (5 M, 50 mL), 70 °C, 7 days.

Product (M)	Analyte	Observed Mass	Expected Mass (relative intensity %)	Error (ppm)	Absolute Intensity	RT (min)
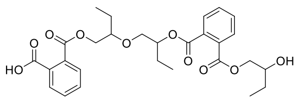 [HO-PA$_2$-BO$_3$-H]	$[M-2H+Na]^{1-}$	551.823	551.190 (100%)	+1148.424	379	0.49
	**$[2M-2H+MeOH+H_2O]^{2-}$**	**553.817**	**554.227 (100%)**	**-739.769**	**1122**	**0.49**
	**$[2M-2H+MeOH+Na]^{2-}$**	**555.807**	**556.212 (100%)**	**+1100.161**	**1476**	**0.49**
	**$[2M-3H+H_2O+K]^{2-}$**	**557.804**	**557.191 (100%)**	**-1098.952**	**1318**	**0.49**
	$[2M-2H+Na+K]^{2-}$	559.806	559.177 (100%)<br>559.676 (61%)	+1124.867<br>+232.277	709	0.49
	$[M-H+MeOH]^{1-}$	561.815	561.235 (100%)	+1033.435	317	0.49
	$[2M-2H+MeOH+K]^{2-}$	563.832	564.199 (100%)	650.480	236	0.49
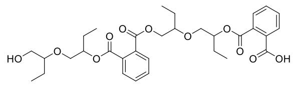 [HO-PA$_2$-BO$_4$-H]	$[2M-2H+MeOH+H_2O]^{2-}$	625.677	626.284 (100)%	-969.209	214	0.49
	$[2M-3H+MeOH+Na]^{2-}$	627.661	628.270 (100%)	-969.328	721	0.49
	**$[2M-3H+H_2O+K]^{2-}$**	**629.652**	**629.249 (100%)**	**+640.446**	**1084**	**0.49**
	**$[2M-2H+Na+K]^{2-}$**	**631.641**	**631.235 (100%)<br>631.736 (69%)**	**+643.184<br>-150.379**	**979**	**0.49**
	$[M-H+MeOH]^{1-}$	633.654	633.292 (100%)	+571.616	612	0.49
	$[2M-3H+MeOH+K]^{2-}$	635.649	636.257 (100%)	-955.589	368	0.49
	$[3M-5H+MeOH+2K]^{3-}$	637.657	637.579 (100%)	+122.338	201	0.49
	$[M-2H+K]^{1-}$	639.674	639.222 (100%)	+707.110	137	0.49
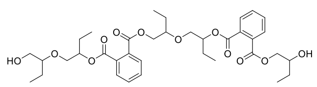 [HO-PA$_2$-BO$_5$-H]	$[2M-2H+MeOH+Na]^{2-}$	699.516	700.327 (100%)	+1158.030	87	0.49
	$[2M-3H+H_2O+K]^{2-}$	701.514	701.306 (100%)	+296.590	294	0.49
	$[2M-2H+Na+K]^{2-}$	703.501	703.292 (100%)	+297.174	438	0.49
	$[M-H+MeOH]^{-}$	705.494	705.349 (100%)	+205.572	512	0.49
	$[2M-3H+MeOH+K]^{2-}$	707.494	708.314 (100%)	+1157.679	356	0.49

2.7 TGA Experiments for P1, P4 and P7 (Table 1)

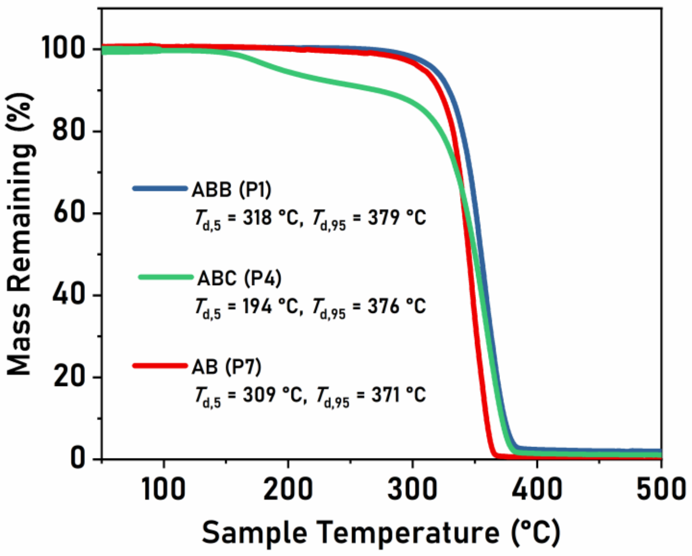

Figure S27: TGA trace of **P1**, **P4** and **P7**

2.8 NMR Spectra of Polymers Described in Table 2.

2.8.1 NMR Spectra for P9, P(PA-PO-PO) (Table 2)

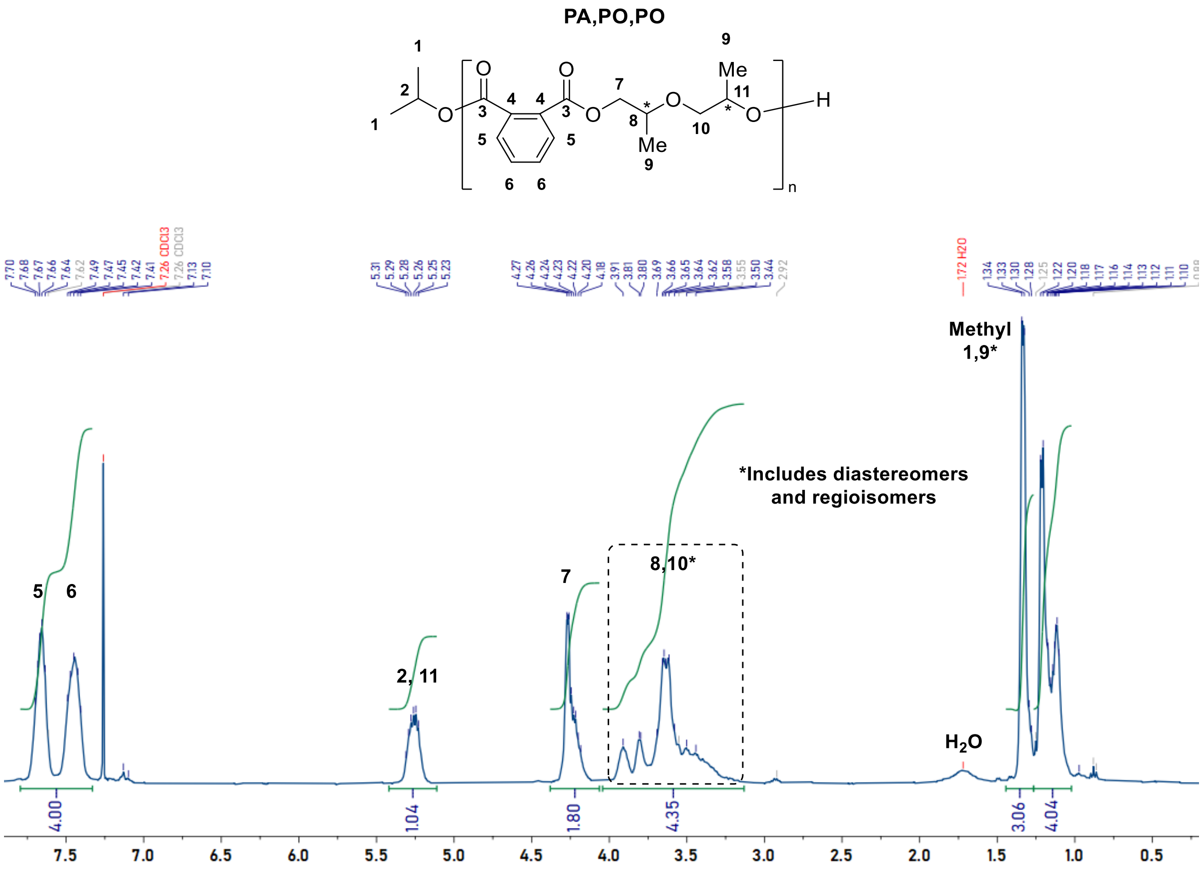

Figure S28: ^{1}H NMR spectrum (400 MHz, $CDCl_3$) of **P9** (Table 2). Reaction conditions: [Cat]:[PA]:[PO]:[THF] = 1: 50: 1419: 0 stopped at 60 mins after work-up in Pentane/DCM.

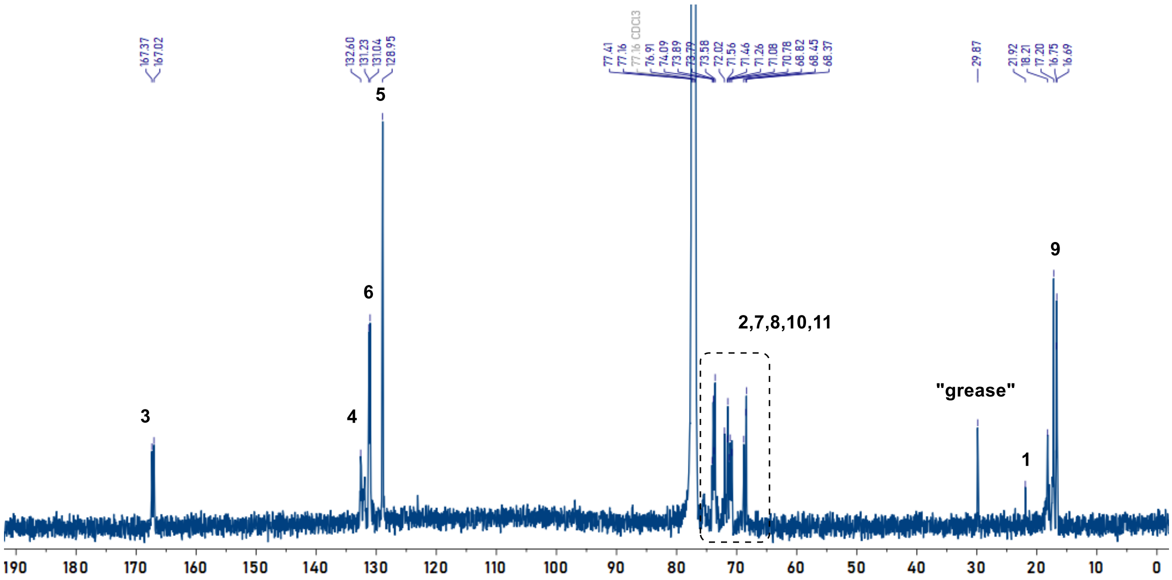

Figure S29: ^{13}C NMR spectrum (128 MHz, $CDCl_3$) of **P9** (Table 2). Reaction conditions: [Cat]:[PA]:[PO]:[THF] = 1: 50: 1419: 0 stopped at 60 mins after work-up in Pentane/DCM.

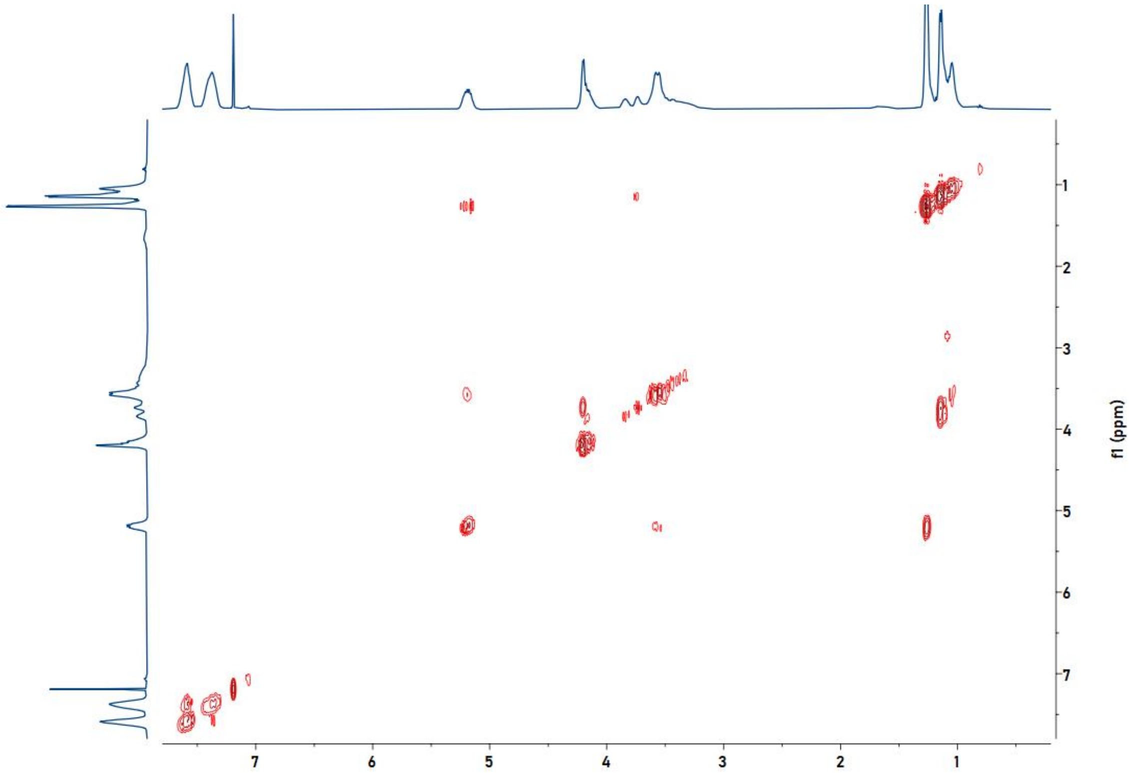

Figure S30: ^{1}H COSY NMR spectrum of **P9** (400 MHz, $CDCl_3$). Reaction conditions: [Cat]:[PA]:[PO]:[THF] = 1: 50: 1419: 0 stopped at 60 mins after work-up in Pentane/DCM.

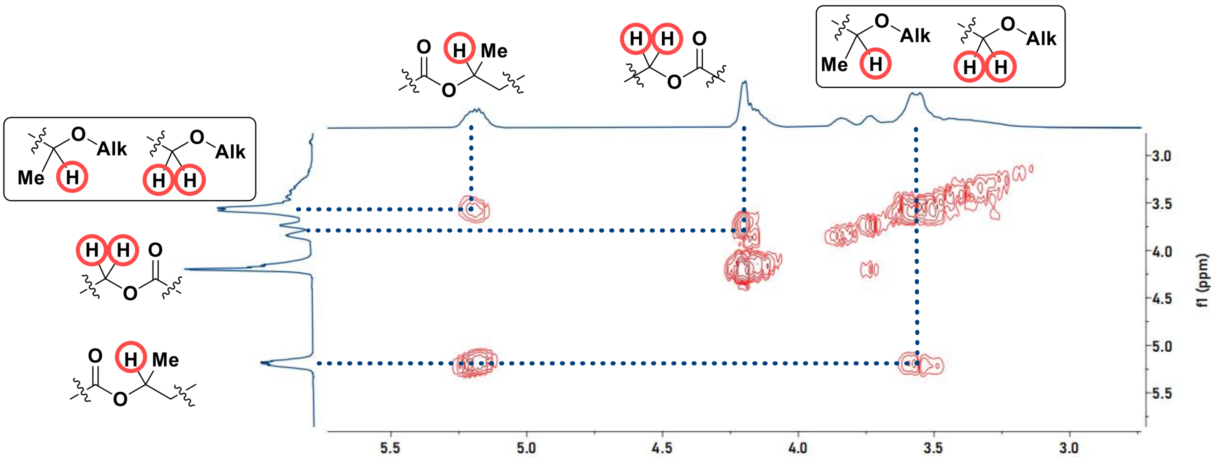

Figure S31: Magnified ^{1}H COSY NMR of **P9** (400 MHz, $CDCl_3$). Reaction conditions: [Cat]:[PA]:[PO]:[THF] = 1: 50: 1419: 0 stopped at 60 mins after work-up in Pentane/DCM.

2.8.2 Spectra for P12, P(PA-PO-PO)-(PA-PO-THF) (Table 2)

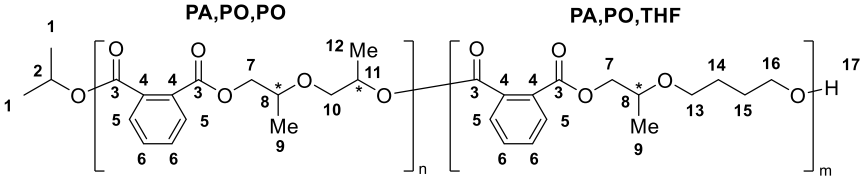

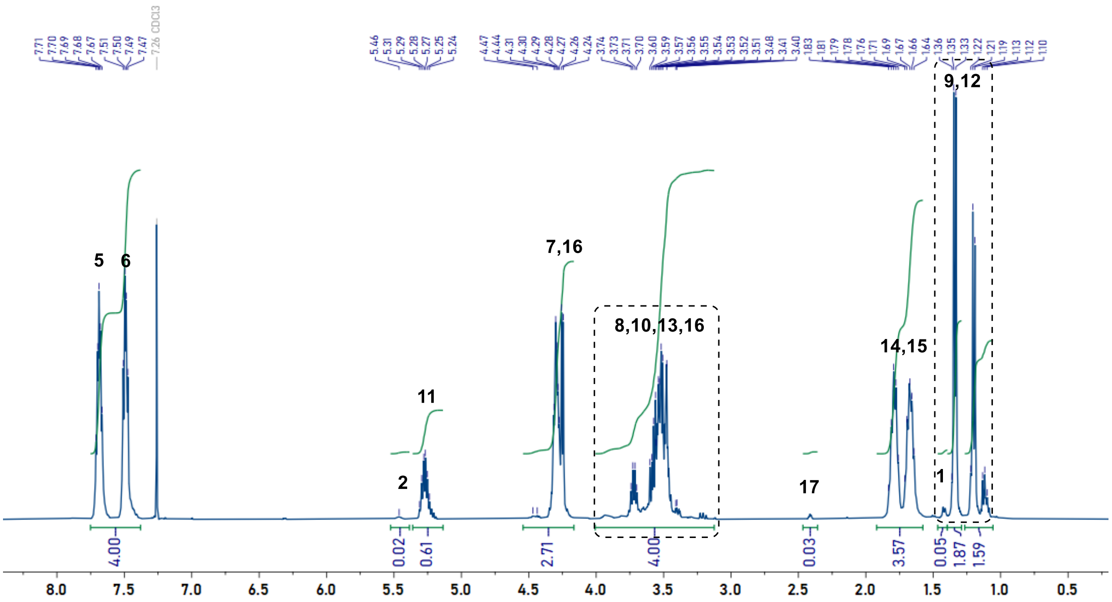

Figure S32: ^{1}H NMR spectrum of **P12** (400 MHz, $CDCl_3$). Reaction conditions: [Cat]:[PA]:[PO]:[THF] = 1: 50: 355: 924 after work-up in MeOH/DCM

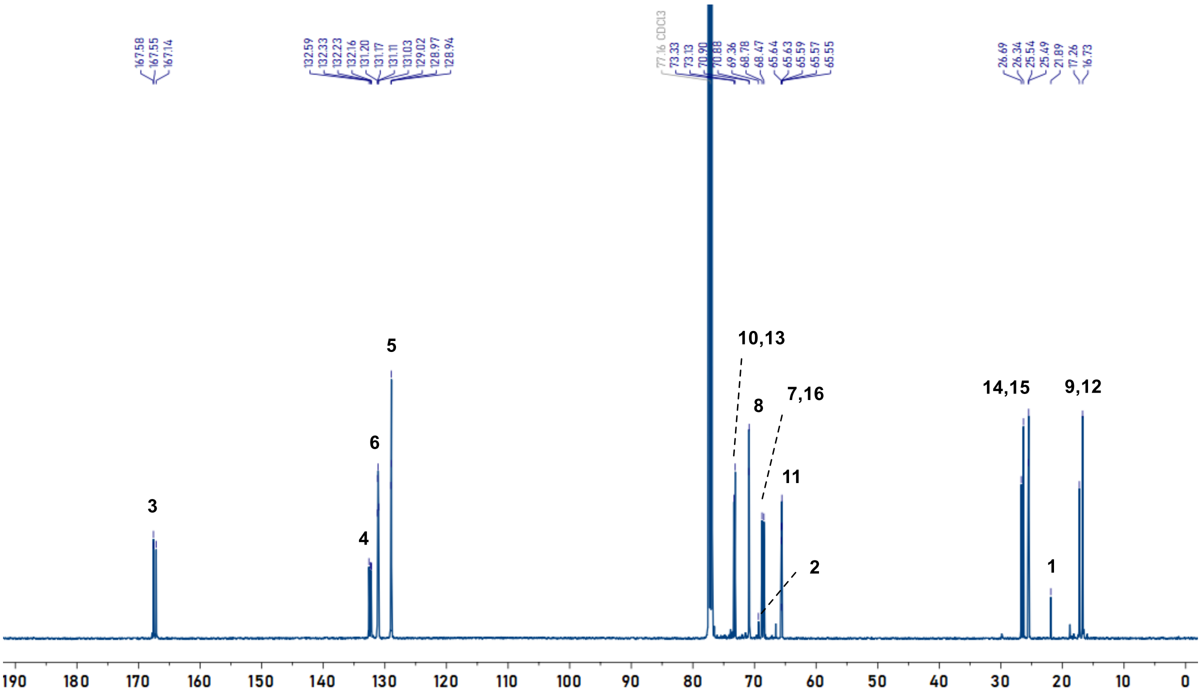

Figure S33: ^{13}C NMR spectrum of **P12** (128 MHz, $CDCl_3$). Reaction conditions: [Cat]:[PA]:[PO]:[THF] = 1: 50: 355: 924 after work-up in MeOH/DCM

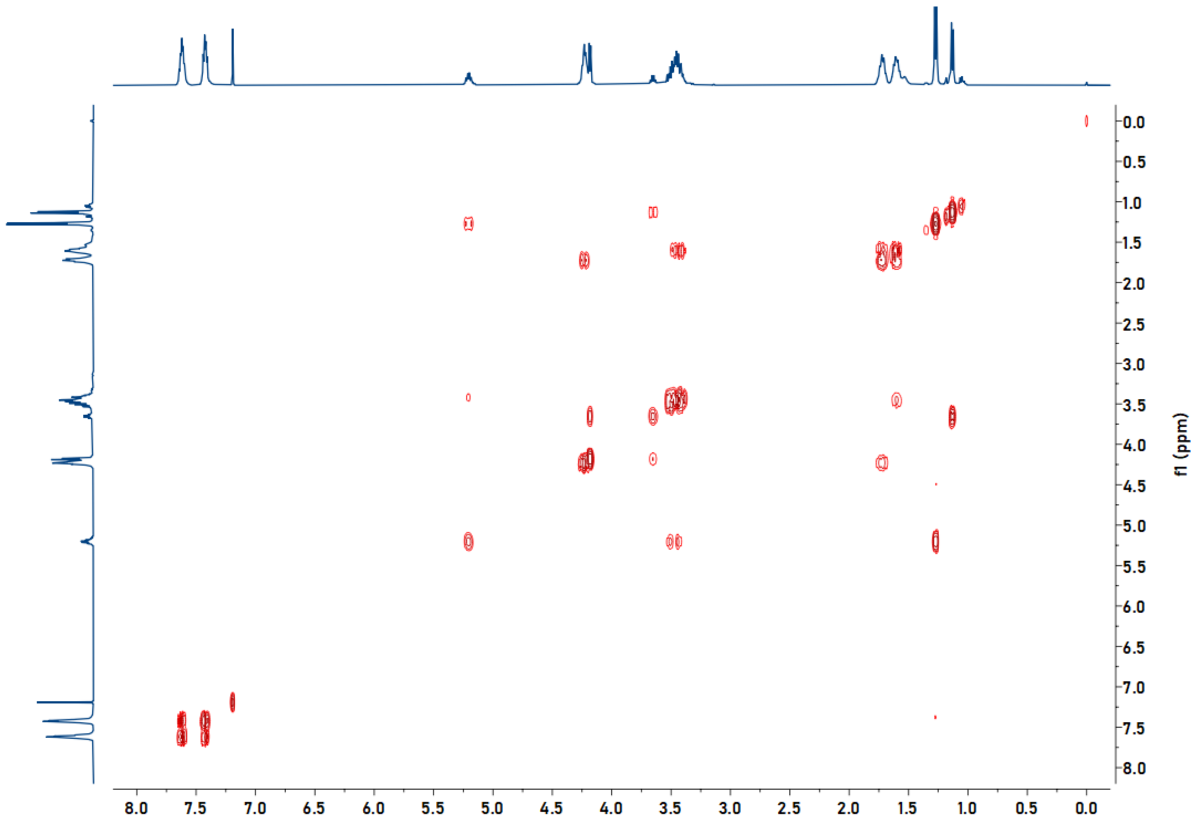

Figure S34: ^{1}H COSY NMR spectrum of **P12** (400 MHz, $CDCl_3$). Reaction conditions: [Cat]:[PA]:[PO]:[THF] = 1: 50: 355: 924 after work-up in MeOH/DCM

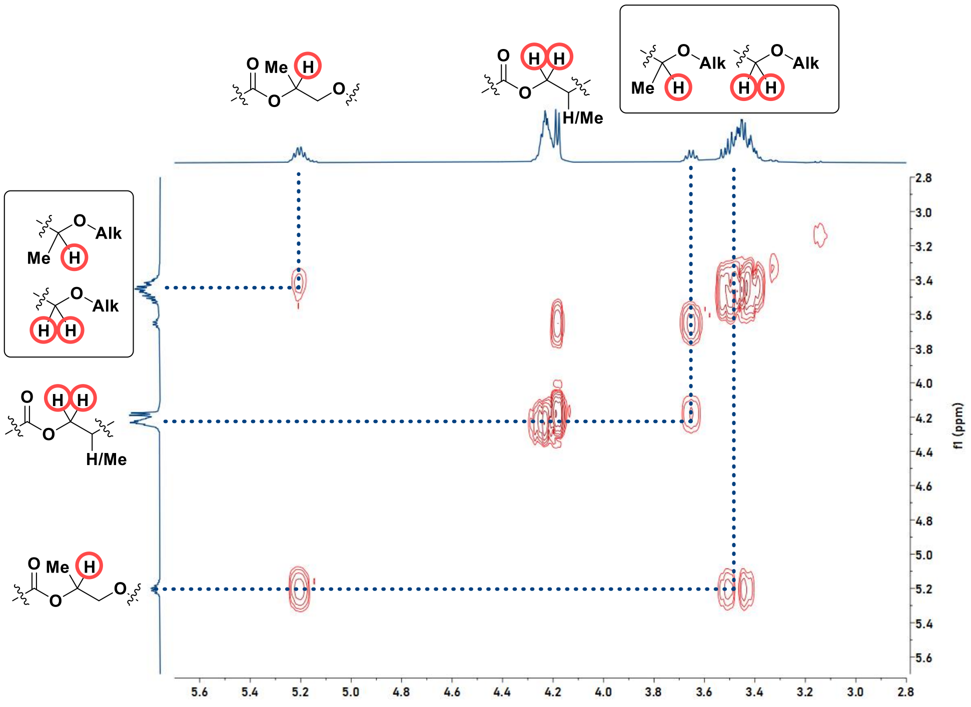

Figure S35: Enlarged ^{1}H COSY NMR spectrum of **P12** (400 MHz, $CDCl_3$). Reaction conditions: [Cat]:[PA]:[PO]:[THF] = 1: 50: 355: 924 after work-up in MeOH/DCM

2.8.3 Spectra for P14 P(PA-BO-BO)-(PA-BO-MeTHF) (Table 2)

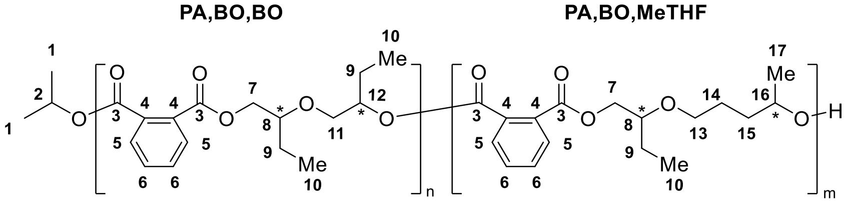

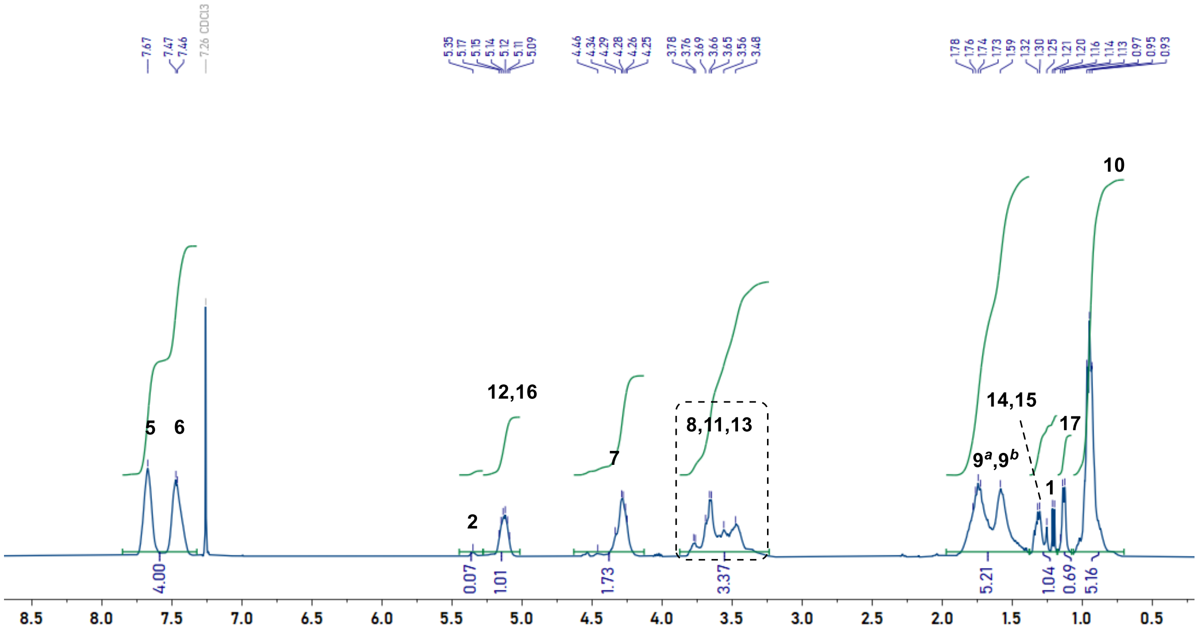

Figure S36: ^{1}H NMR spectrum of **P14** (400 MHz, $CDCl_3$). Reaction Conditions: [Cat]:[PA]:[BO]:[MeTHF] = 1: 50: 288: 744 after work-up in MeOH/DCM.

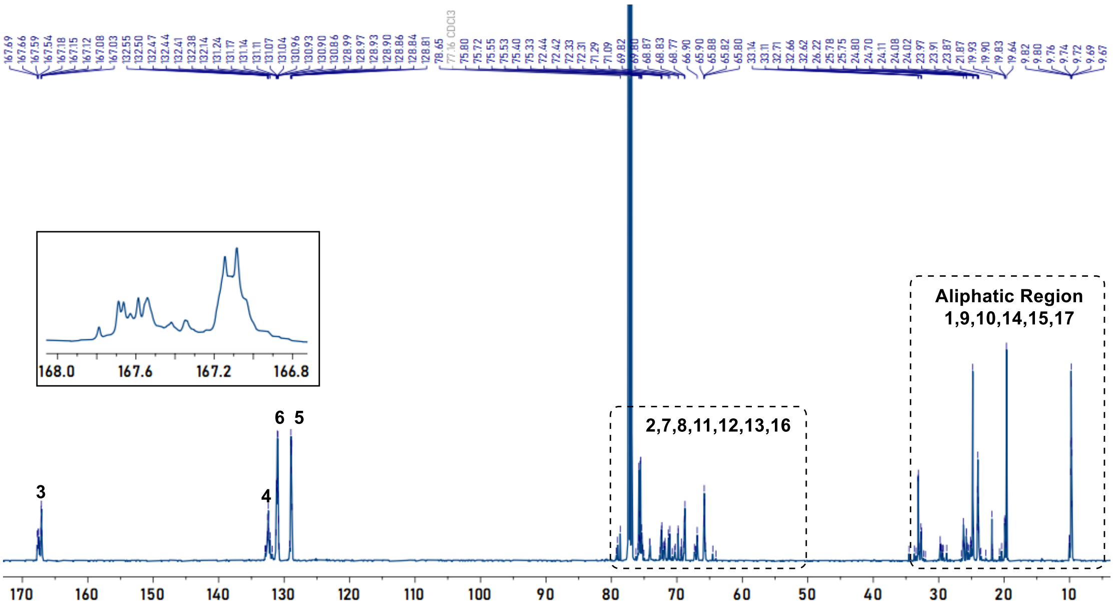

Figure S37: ^{13}C NMR spectrum for **P14** (128 MHz $CDCl_3$). Enlarged carbonyl region illustrates the complicated regio- and diastereoselectivity of this reaction which uses two racemic monomers. Reaction Conditions: [Cat]:[PA]:[BO]:[MeTHF] = 1: 50: 288: 744 after work-up in MeOH/DCM.

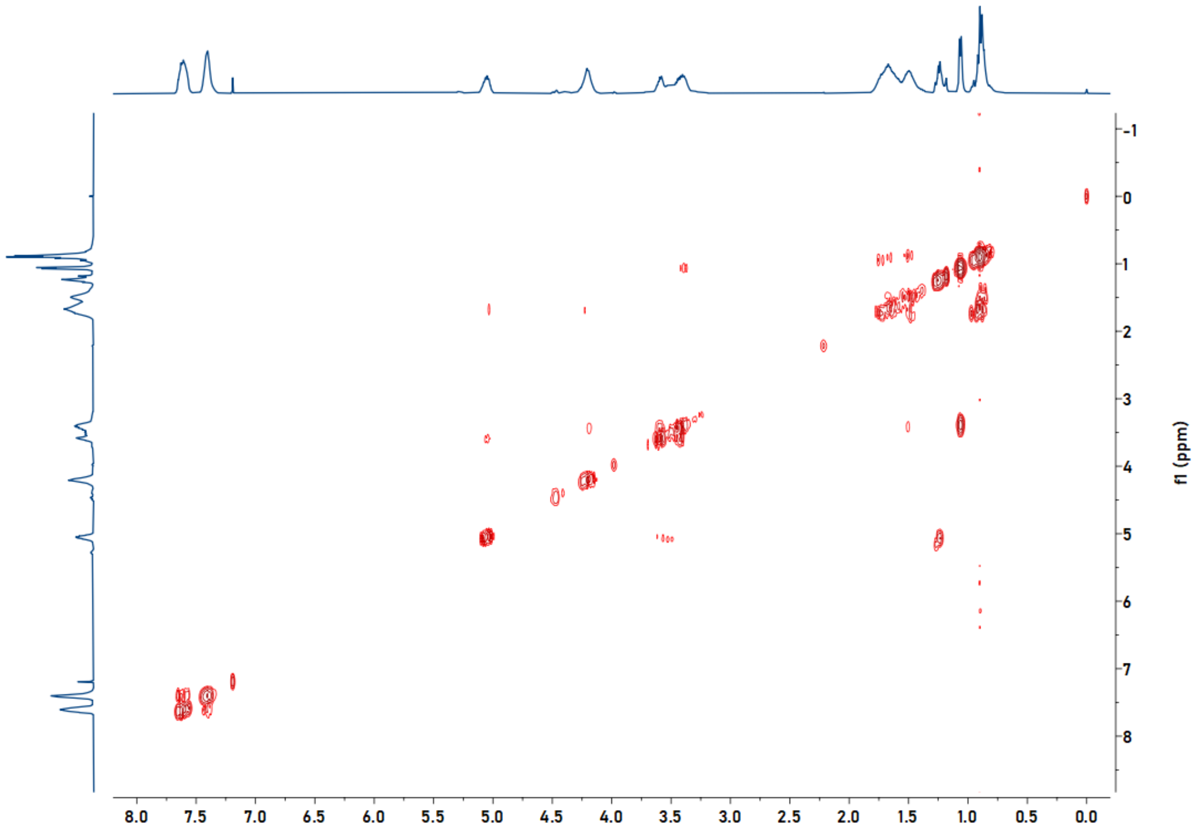

Figure S38: ^{1}H NMR spectrum of **P14** (400 MHz, $CDCl_3$). Reaction Conditions: [Cat]:[PA]:[BO]:[MeTHF] = 1: 50: 288: 744 after work-up in MeOH/DCM.

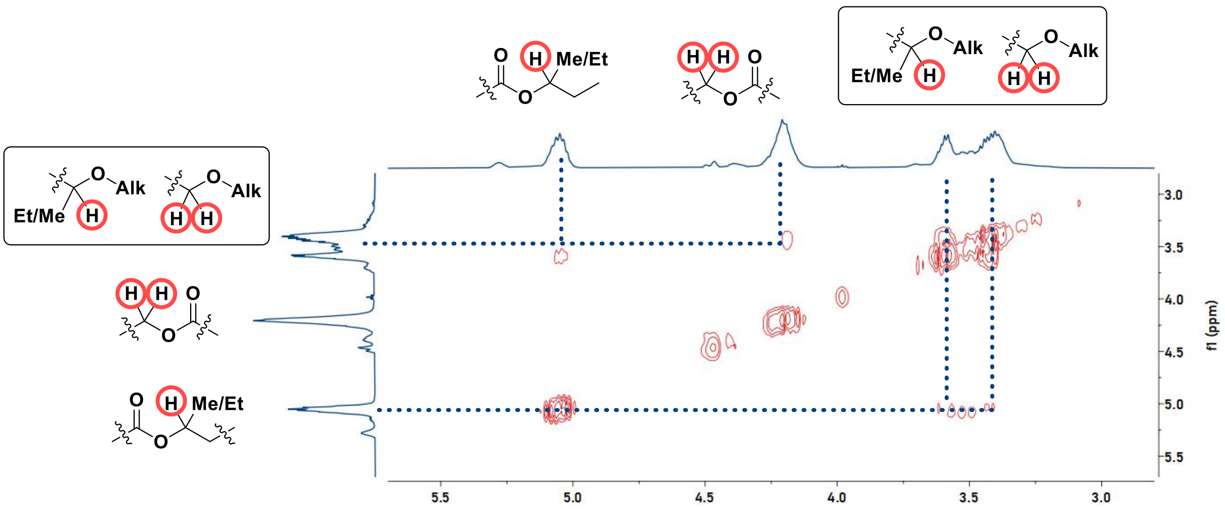

Figure S39: Magnified ^{1}H NMR spectrum of **P14** (400 MHz, $CDCl_3$). Reaction Conditions: [Cat]:[PA]:[BO]:[MeTHF] = 1: 50: 288: 744 after work-up in MeOH/DCM.

2.8.4 Spectra of P15 P(PA-OX-OX) (Table 2)

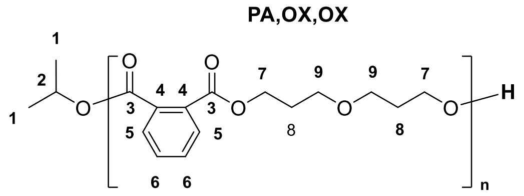

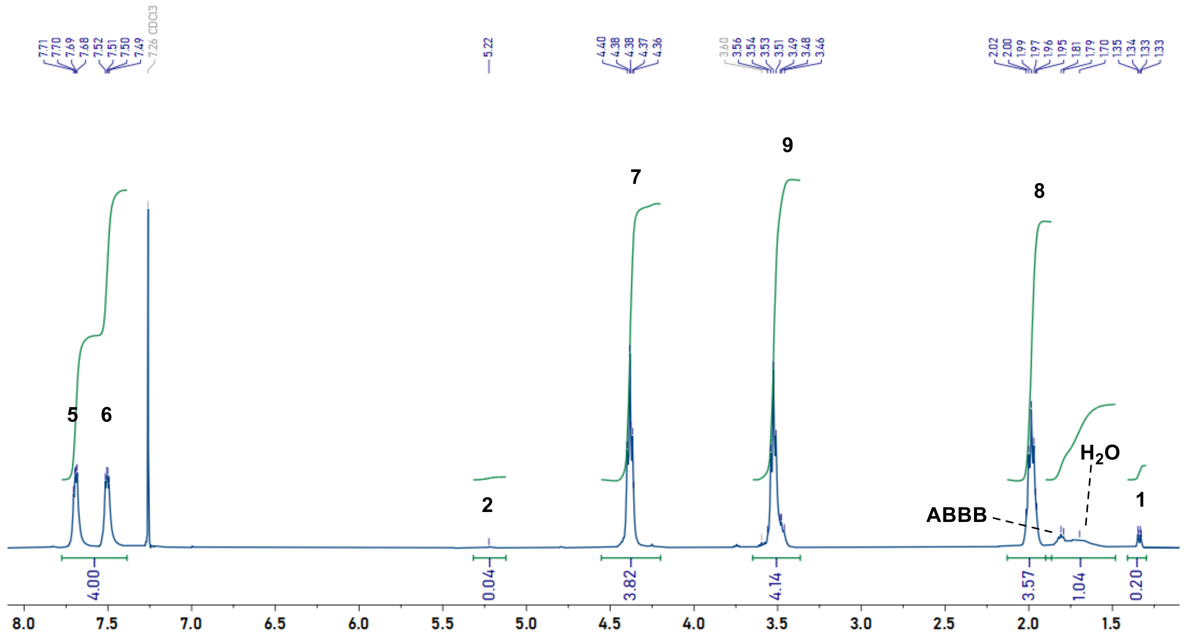

Figure S40: ^{1}H NMR spectrum of **P15** (400 MHz, $CDCl_3$). Reaction conditions: [Cat]:[PA]:[OX]:[THF] = 1: 50: 1527: 0 after work-up in Pentane/DCM.

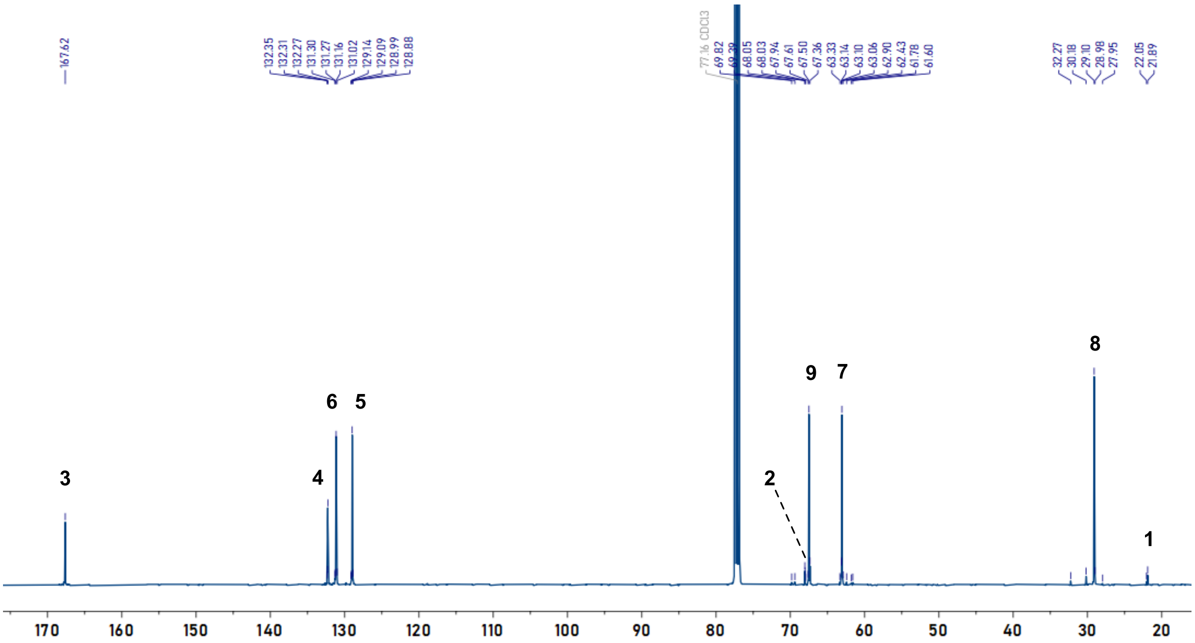

Figure S41: ^{13}C NMR spectrum of **P15** (128 MHz, $CDCl_3$). Reaction conditions: [Cat]:[PA]:[OX]:[THF] = 1: 50: 1527: 0 after work-up in Pentane/DCM.

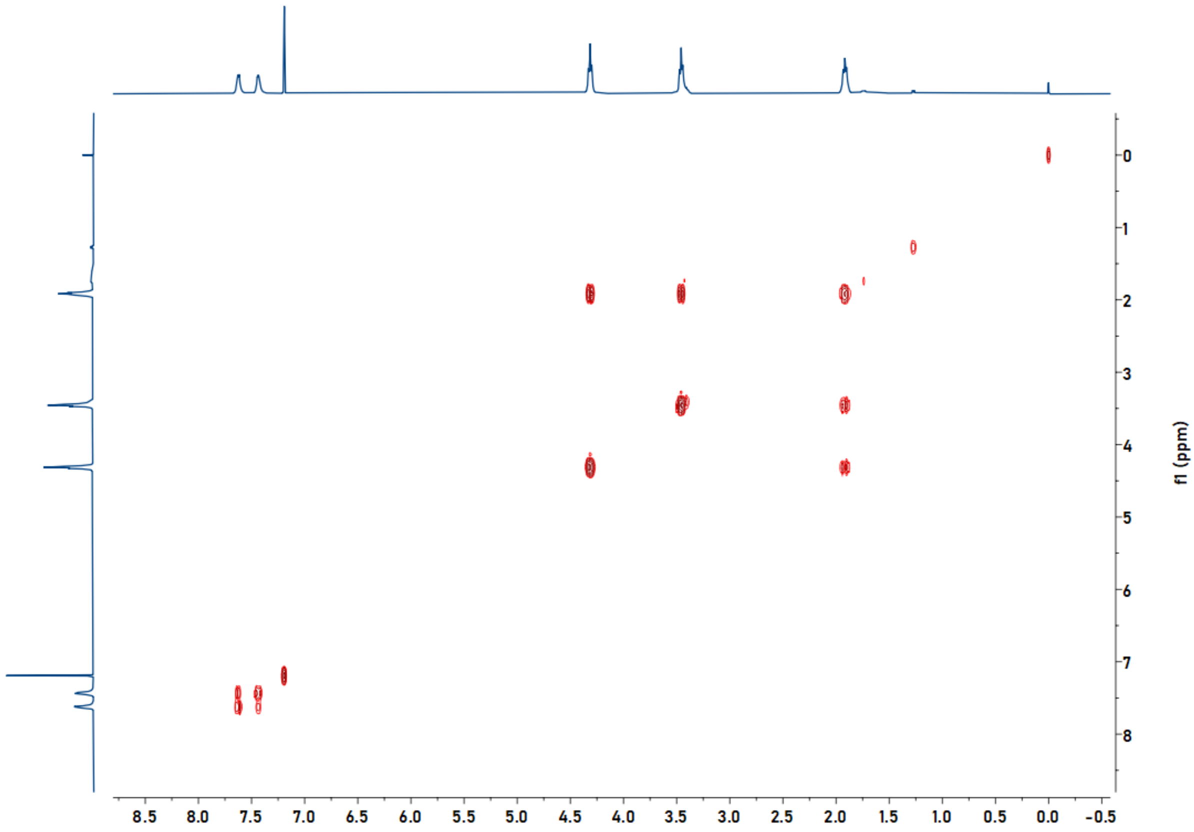

Figure S42: ^{1}H COSY NMR spectrum of **P15** (400 MHz, $CDCl_3$). Reaction conditions: [Cat]:[PA]:[OX]:[THF] = 1: 50: 1527: 0 after work-up in Pentane/DCM.

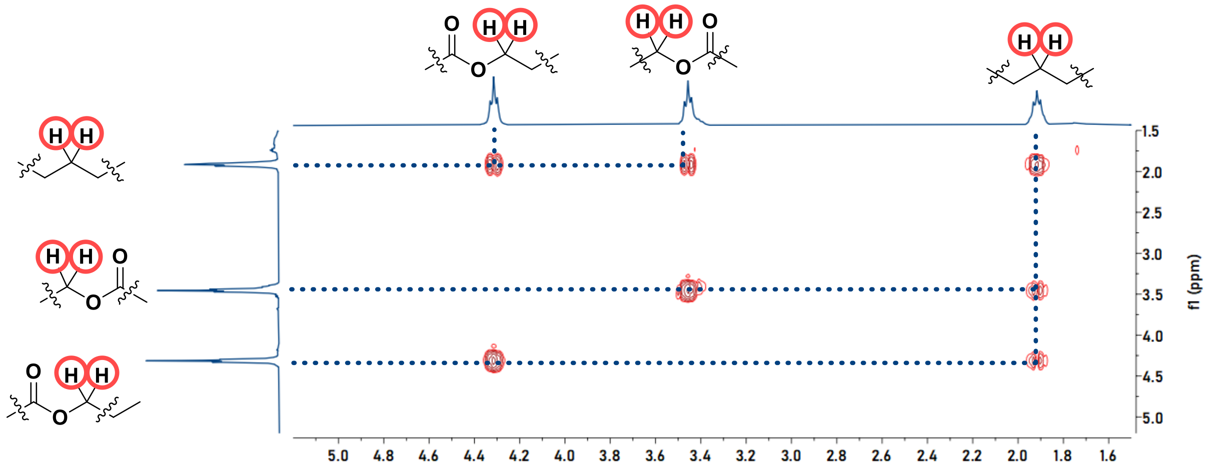

Figure S43: Magnified ^{1}H COSY NMR spectrum of **P15** (400 MHz, $CDCl_3$). Reaction conditions: [Cat]:[PA]:[OX]:[THF] = 1: 50: 1527: 0 after work-up in Pentane/DCM.

2.8.5 Spectra for P16 P(PA-OX-OX)-(PA-OX-THF) (Table 2)

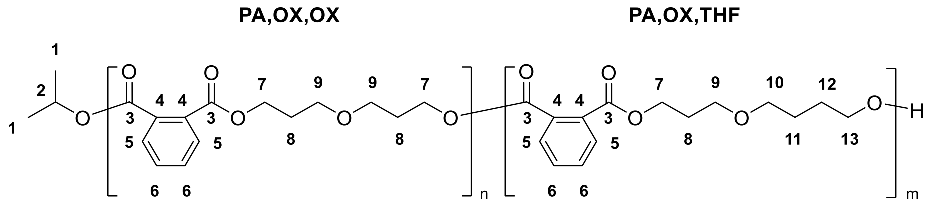

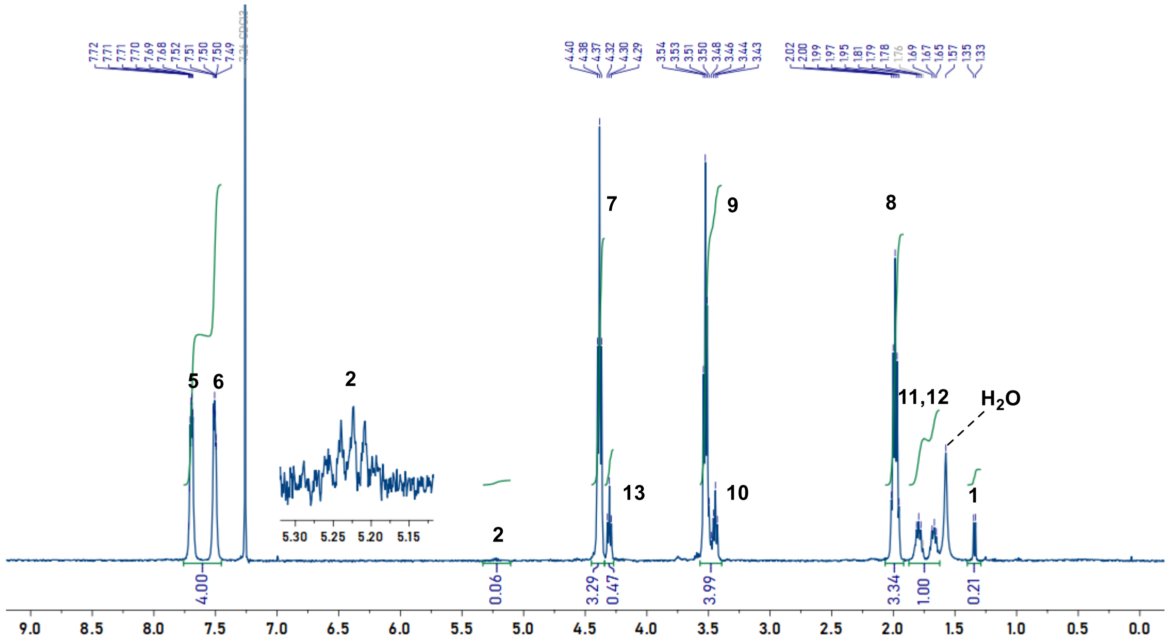

Figure S44: ^{1}H NMR spectrum of **P16** (400 MHz, $CDCl_3$). Reaction conditions: [Cat]:[PA]:[OX]:[THF] = 1: 50: 382: 924 after work-up in MeOH/DCM.

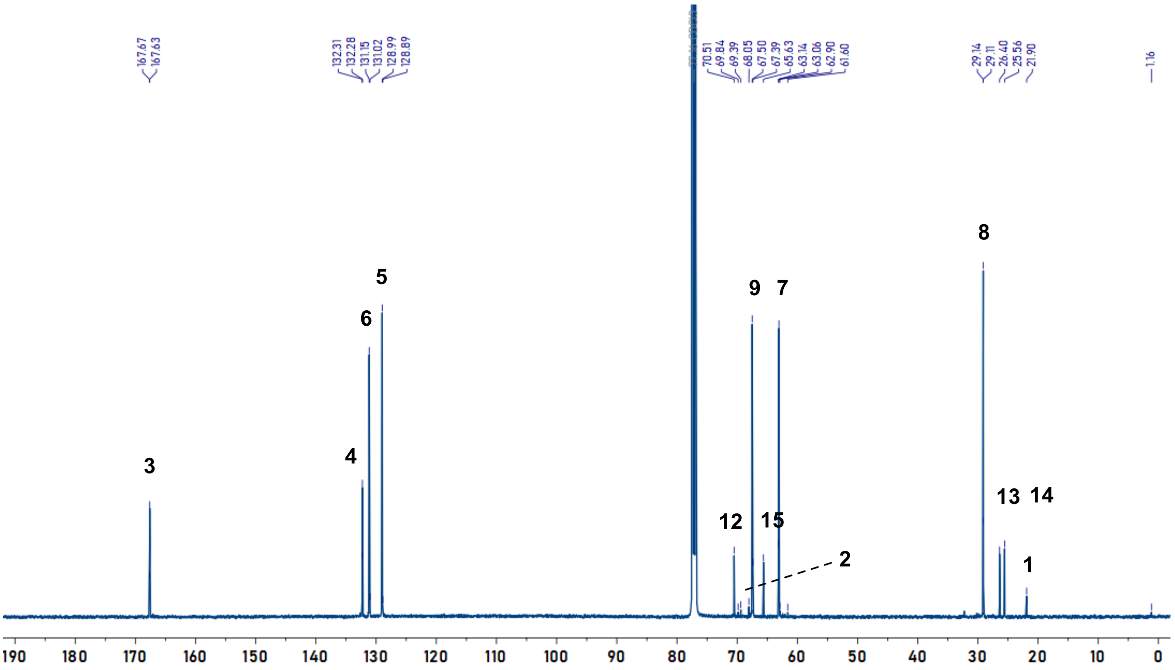

Figure S45: ^{13}C NMR spectrum of **P16** (128 MHz, $CDCl_3$). Reaction conditions: [Cat]:[PA]:[OX]:[THF] = 1: 50: 382: 924 after work-up in MeOH/DCM.

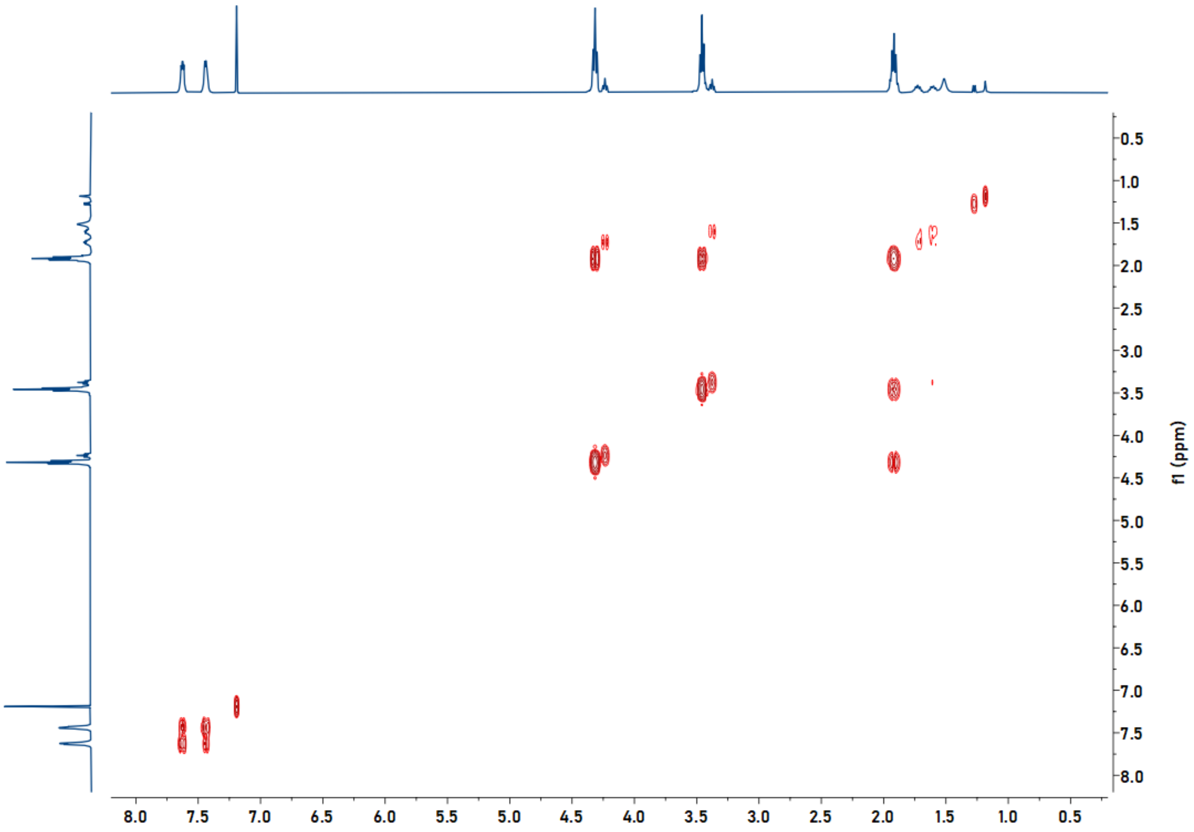

Figure S46: ^{1}H COSY NMR spectrum of **P16** (400 MHz, $CDCl_3$). Reaction conditions: [Cat]:[PA]:[OX]:[THF] = 1: 50: 382: 924 after work-up in MeOH/DCM.

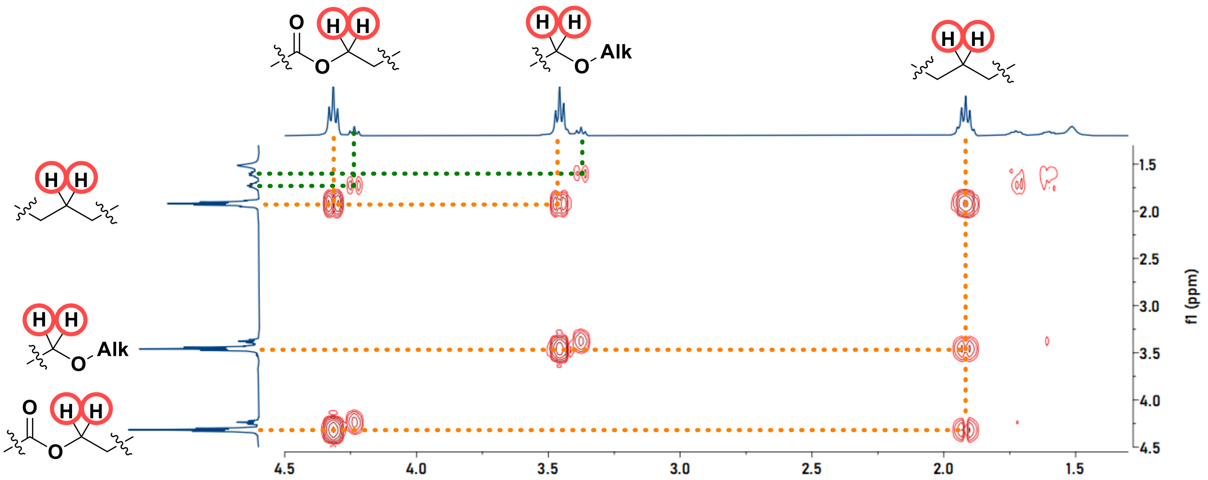

Figure S47: Magnified ^{1}H COSY NMR spectrum of **P16** (400 MHz, $CDCl_3$). P(OX) correlations (orange), P(THF) correlations (green). Reaction conditions: [Cat]:[PA]:[OX]:[THF] = 1: 50: 382: 924 after work-up in MeOH/DCM.

2.9 GPC Data for Polymers Described in Table 2

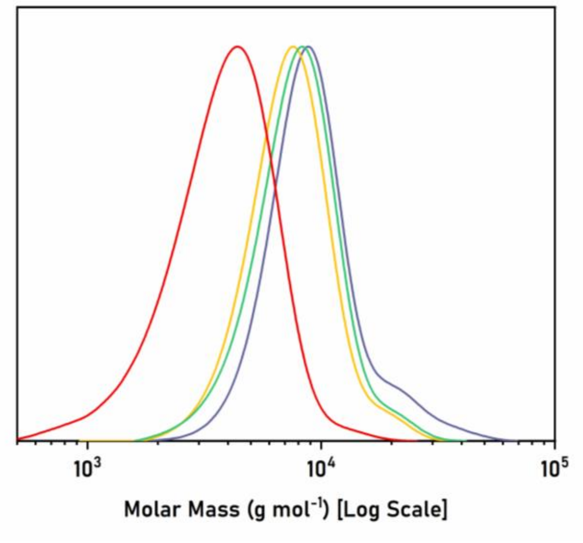

Figure S48: GPC chromatograms for polymers described in Table 2, **P9-12**: **P9** (♠) [Cat]:[PA]:[PO]:[THF] = 1: 50: 1419: 0, M_n = 3.2 kg mol^{-1}, Đ = 1.32, time = 1 h; **P10** (♣) [Cat]:[PA]:[PO]:[THF] = 1: 50: 1064: 308, M_n = 7.1 kg mol^{-1}, Đ = 1.20, time = 5 h; **P11** (♦) [Cat]:[PA]:[PO]:[THF] = 1: 50: 710: 616, M_n = 6.7 kg mol^{-1}, Đ = 1.24, time = 5 h. **P12** (♥) [Cat]:[PA]:[PO]:[THF] = 1: 50: 355: 924, M_n = 8.3 kg mol^{-1}, Đ = 1.24, time = 5 h.

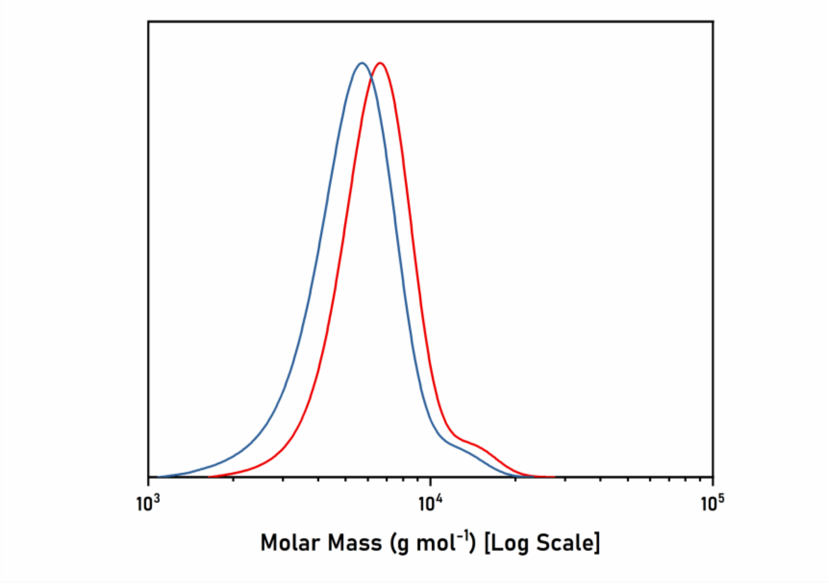

Figure S49: GPC chromatograms for polymers described in Table 2, **P13-14**: **P13** (♥) [Cat]:[PA]:[BO]:[MeTHF] = 1: 50: 863: 248, M_n = 4.9 kg mol^{-1}, Đ = 1.17, time = 5 h; **P14** (♠) [Cat]:[PA]:[BO]:[MeTHF] = 1: 50: 288: 744, M_n = 6.1 kg mol^{-1}, Đ = 1.24, time = 5 h.

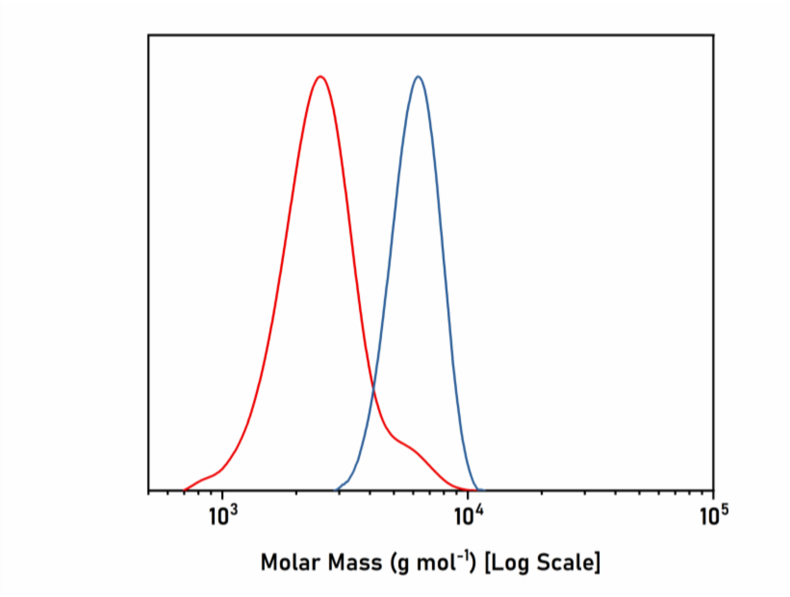

Figure S50: GPC chromatograms for polymers described in Table 2, **P15-P16**: **P15** (♠) [Cat]:[PA]:[OX]:[THF] =1: 50: 1527: 0, $M_{n,GPC}$ = 2.3 kg mol^{-1} ($M_{n,NMR}$ = 7.6 kg mol^{-1}), Đ = 1.16, time = 5 h; **P16** (♥) [Cat]:[PA]:[OX]:[THF] = 1: 50: 382: 924, M_n = 5.9 kg mol^{-1} ($M_{n,NMR}$ = 8.0 kg mol^{-1}), Đ = 1.05, time = 24 h.

2.10 DSC Data for Polymers Described in Table 2

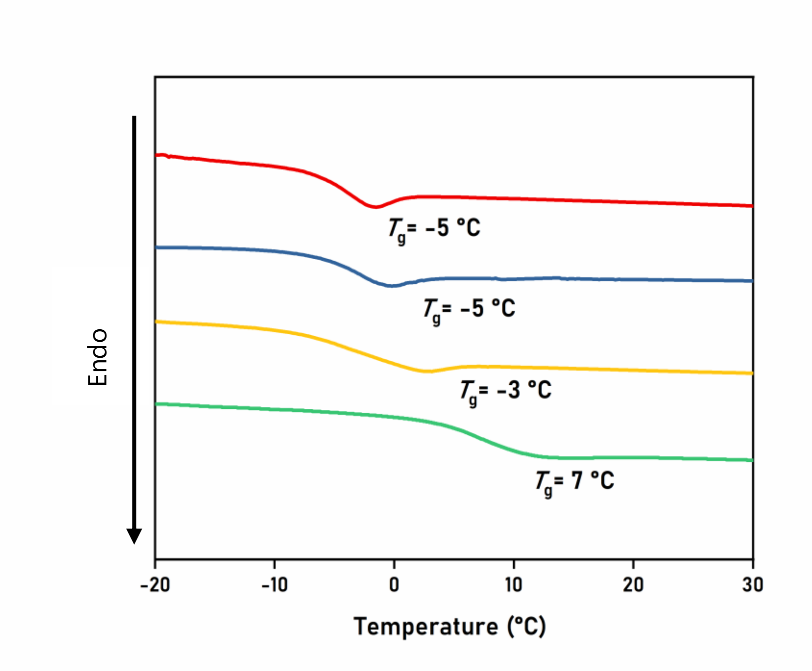

Figure S51: DSC Thermograms for Polymers **P9-12**. Data are presented at the glass transition temperatures.

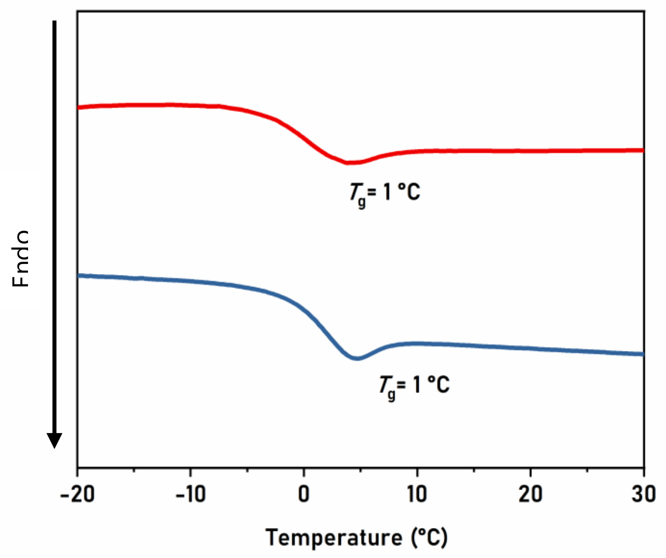

Figure S52: DSC thermograms for **P13** and **14**.

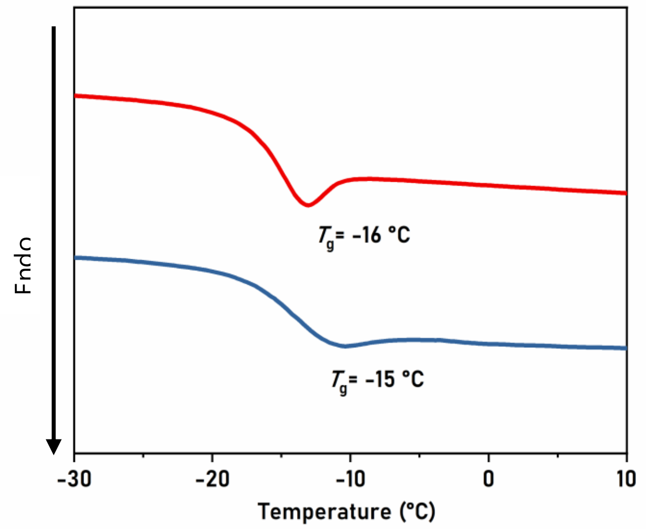

Figure S53: DSC Thermograms for **P15** and **16**.

3.0 Kinetic Experiments

3.1 Alternative fits for order in catalyst

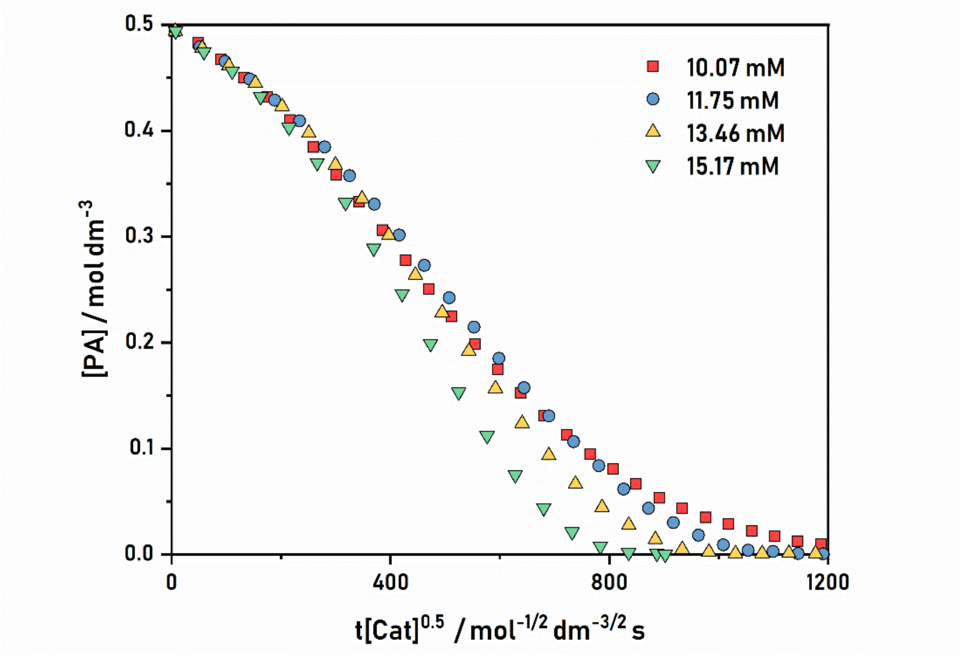

Figure S54: An inferior fit for the 0.5th order dependence on catalyst concentration is determined from the plots of [BO] versus t[catalyst]x, x = 0.5 where [catalyst] = 10.07–15.17 mM, [PA] = 0.494 M and BO = 11.5 M, 50 °C.

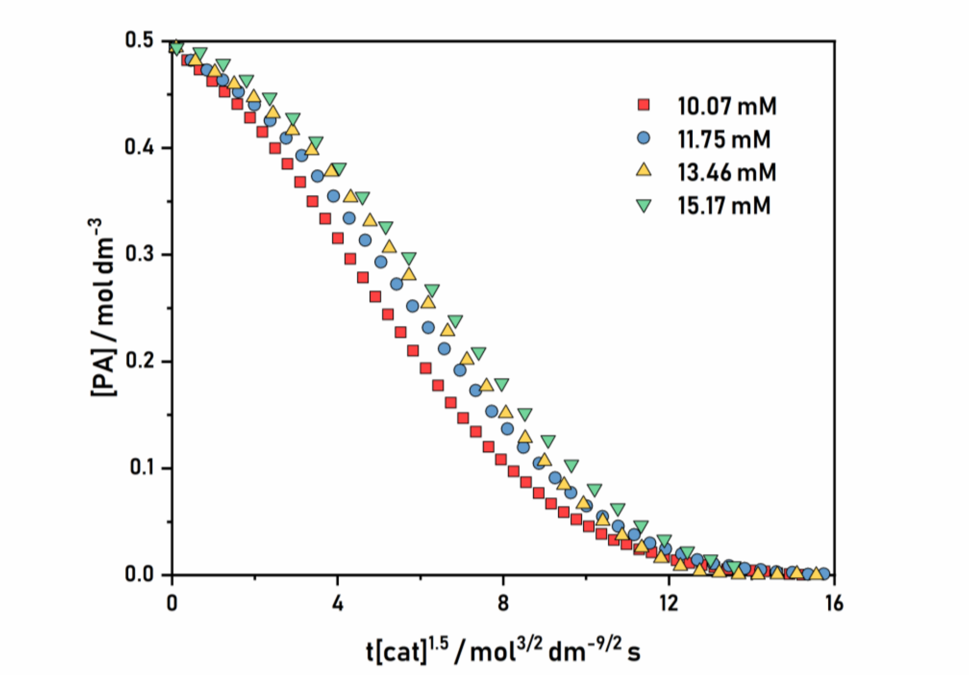

Figure S55: An inferior fit for the 1.5th order dependence on catalyst concentration is determined from the plots of [BO] versus t[catalyst]x, x = 1.5 where [catalyst] = 10.07–15.17 mM, [PA] = 0.494 M and BO = 11.5 M, 50 °C.

3.2 First Order Fits for Epoxide Concentration Dependence

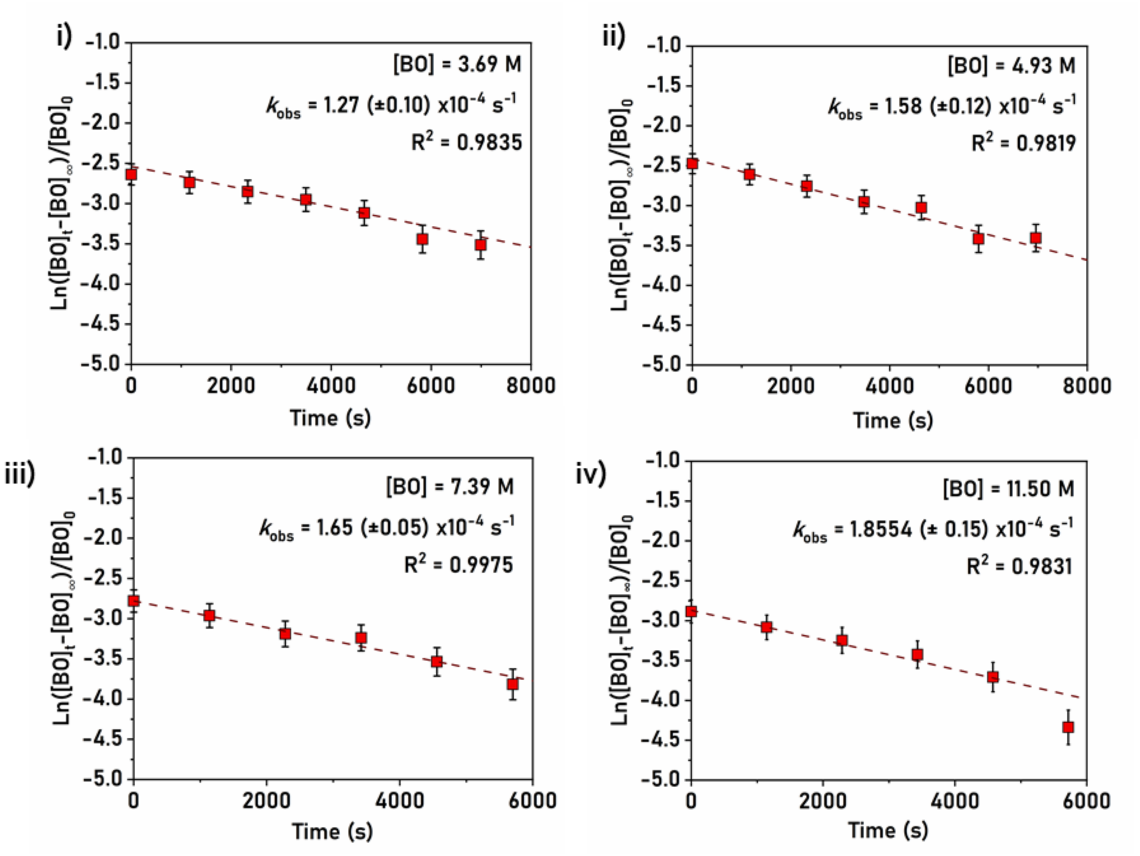

Figure S56: The order in epoxide concentration determined from the linear fit to plots of ln{$[BO]_t$-$[BO]_\infty$/$[BO]_0$} versus time where $[BO]_0$ = 3.7–11.5 M. Conversion data obtained from ^{1}H NMR spectroscopy kinetic experiments using method outlined in S2.1.3. Each plot is an average of duplicate runs, with an error of ±5%. Conditions: [catalyst] = 10 mM, [PA] = 420 mM**,** $[BO]_0$ = 3.7–11.5 M, [THF] = 0.0-6.6 M, total volume of BO+THF solution = 700 µL after mixing, 50 °C.

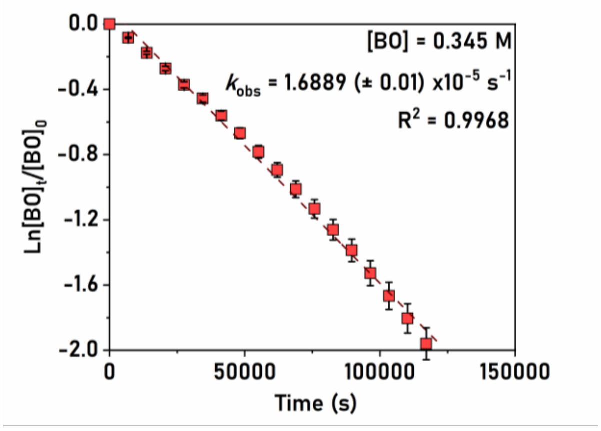

Figure S57: The order in epoxide concentration determined from the linear fit to plots of ln$[BO]_t$/$[BO]_0$ versus time, where $[BO]_0$ = 0.345 mM (a sub-stoichiometric quantity vs. [PA]). Data are determined from ^{1}H NMR

kinetic experiments and apply to Fig. 5A. Conditions: [catalyst] = 10 mM, [PA] = 420 mM, $[BO]_0$ = 0.345 M (21 µL), [d_8-THF] = 12.3 M (700 µL), 50 °C.

3.3 Reaction of 1 and Phthalic Anhydride

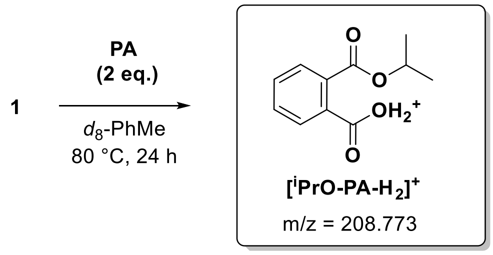

In a glovebox, **1** (47 mg, 50 µmol) and phthalic anhydride (15 mg, 100 µmol) were weighed into a vial and dissolved in d_8-toluene (≈ 0.7 mL). The reaction mixture was transferred to a Young Taps NMR tube, sealed and heated to 80 °C, for 3 hours. ^{1}H and ^{19}F NMR spectroscopic analysis was complex, but revealed consumption of **1** and the formation of new species showing signals for both PA and the ligand (Figs. S57-58). LC-MS analysis revealed the presence for the expected product of phthalic anhydride reaction with iso-propoxide, i.e. $[^iPrO\text{-}PA\text{-}H_2]^+$ (Fig. S59). Attempts to isolate single crystals from the reaction mixture produced only blocks of unreacted phthalic anhydride.

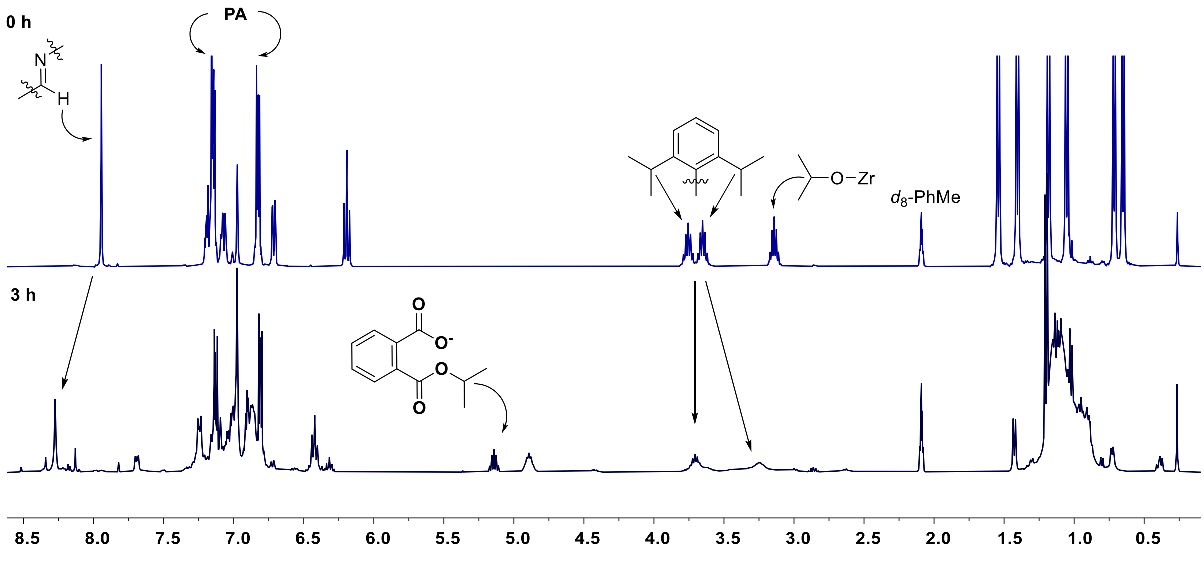

Figure S58: ^{1}H NMR spectrum of the product from the reaction of [**1**]:[PA] = 1: 2 at 0 h (top), and 3 h (bottom), (400 MHz, d_8-PhMe). The spectra show the depletion of **1** and the formation of broad resonances for the complex (ligands).

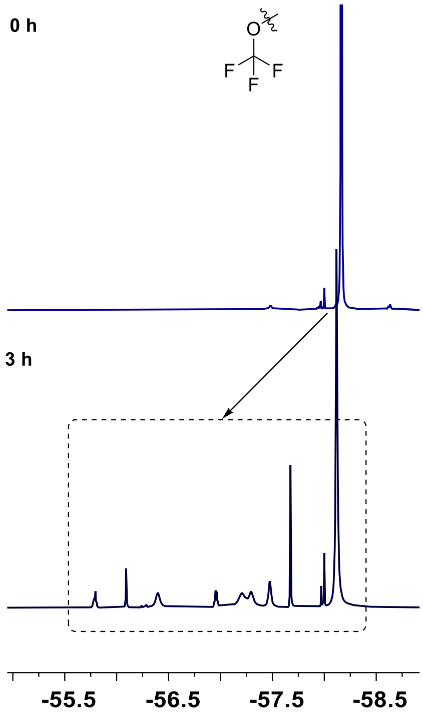

Figure S59: ^{19}F NMR spectrum of the product from the reaction of [**1**]:[PA] = 1: 2 at 0 h (top), and 3 h (bottom), (376 MHz, d_8-PhMe). The spectrum depicts the depletion of **1** and the formation of new species showing multiple ^{19}F resonances.

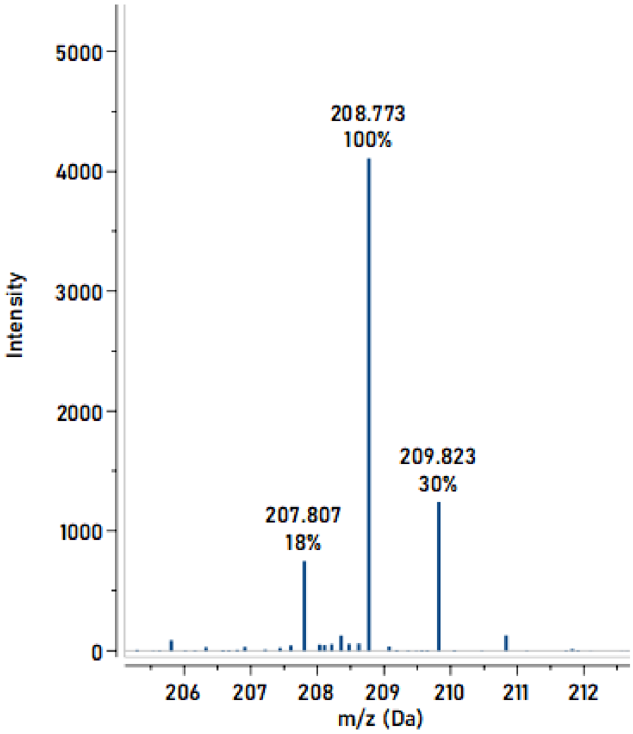

Figure S60: Negative ESI-MS spectrum for the product from the reaction of [**1**]:[PA] = 1: 2, at 3.44 minutes.

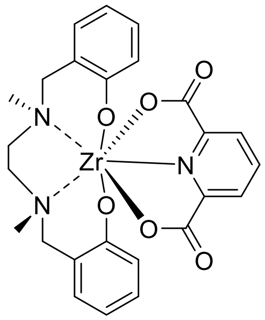

Huhn 2016

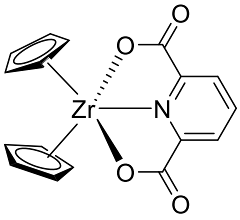

Brintzinger 1993

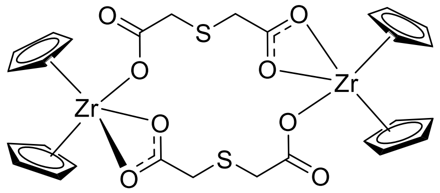

Hey-Hawkins 2001

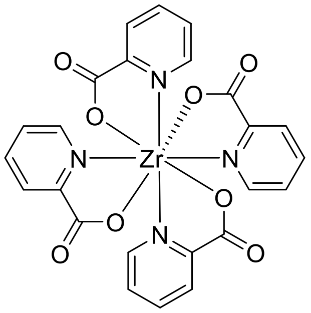

Steyn 2011

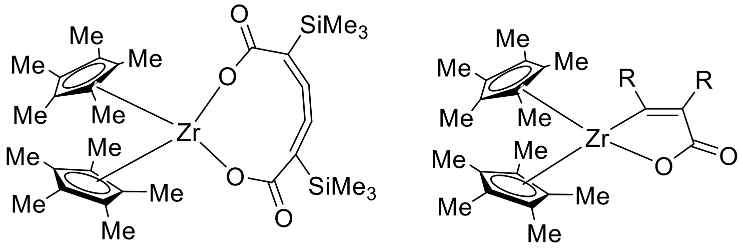

Rosenthal 1999

Chart S2: Examples of known Zr(IV)-carboxylate structures in the CCDC[5-9]

4.0 Single Crystal X-ray Diffraction Data for Complex 1[10-12]

**Crystal data**	
Local Code	002RWFK21
CCDC deposition number	2145249
Chemical formula	$C_{46}H_{56}F_6N_2O_6Zr{\cdot}0.25(C_6H_{14})$
M_r	959.69
Crystal system, space group	Triclinic, *P*-1
Temperature (K)	150
a, *b*, *c* (Å)	12.2492 (2), 19.5274 (5), 20.7866 (4)
α, β, γ (°)	102.186 (2), 97.720 (2), 97.518 (2)
V ($Å^3$)	4750.48 (18)
Z	4
Radiation type	Cu *K*α
μ (mm^{-1})	2.51
Crystal size (mm)	0.23 × 0.14 × 0.06
**Data collection**	
Diffractometer	SuperNova, Dual, Cu at home/near, Atlas
Absorption correction	Multi-scan<br>*CrysAlis PRO* 1.171.41.81a (Rigaku Oxford Diffraction, 2020) Empirical absorption correction using spherical harmonics, implemented in SCALE3 ABSPACK scaling algorithm.
T_{min}, T_{max}	0.702, 1.000
No. of measured, independent and observed [$I > 2\sigma(I)$] reflections	102635, 19734, 14505
R_{int}	0.103
$(\sin \theta/\lambda)_{max}$ ($Å^{-1}$)	0.631
**Refinement**	
$R[F^2 > 2\sigma(F^2)]$, $wR(F^2)$, S	0.049, 0.129, 1.02
No. of reflections	19734
No. of parameters	1151
H-atom treatment	H-atom parameters constrained
$\Delta\rho_{max}$, $\Delta\rho_{min}$ (e $Å^{-3}$)	0.80, −0.88

Computer programs: *CrysAlis PRO* 1.171.41.81a (Rigaku OD, 2020), *SHELXT* (Sheldrick, 2015), *SHELXL* (Sheldrick, 2015), Olex2 (Dolomanov *et al.*, 2009).

5.0 References

(1) Durr, C. B.; Williams, C. K., New Coordination Modes for Modified Schiff Base Ti(IV) Complexes and Their Control over Lactone Ring-Opening Polymerization Activity. *Inorg. Chem.* **2018,** *57* (22), 14240-14248.

(2) Deacy A. C.; Moreby, E.; Phanopoulos, A.; Williams, C. K., Co(III)/Alkali-Metal(I) Heterodinuclear Catalysts for the Ring-Opening Copolymerization of CO_2 and Propylene Oxide. *J. Am. Soc. Chem.* **2020,** *142* (45), 19150-19160.

(3) Bester, K.; Bukowska, A.; Myśliwiec, B.; Hus, K.; Tomczyk, D.; Urbaniak, P.; Bukowski, W., Alternating ring-opening copolymerization of phthalic anhydride with epoxides catalysed by salophen chromium(III) complexes. An effect of substituents in salophen ligands. *Polym. Chem.,* **2018,** *9* (16), 2147-2156.

(4) Kember, M. R.; Williams, C. K., Efficient Magnesium Catalysts for the Copolymerization of Epoxides and CO2; Using Water to Synthesize Polycarbonate Polyols, *J. Am. Chem. Soc.*, **2012**, *134*, 15676-15679.

(5) Schneider, F.; Zhao, T.; Huhn, T., Cytotoxic heteroleptic heptacoordinate salan zirconium(IV)-bis-chelates – synthesis, aqueous stability and X-ray structure analysis, *Chem. Commun.*, **2016**, *52*, 10151-10154.

(6) Niemann, U.; Diebold, J.; Troll, C.; Rief, U.; Brintziner, H.-H., Zirconocene dipicolinate and related dicarboxylate complexes: Coordination geometries and reactivities, *J. Organomet. Chem*, **1993**, *456*, 195-204.

(7) Wenzel, B.; Lönnecke, P.; Hey-Hawkins, E., Early-Transition-Metal Macrocycles as Metalloligands: Synthesis and Structure of Dinuclear Zirconocene Thioglycolates $[Cp°_2Zr\{OOC(CH_2SCH_2)_nCOO\}_2ZrCp°_2]$ (*n* = 1, 2) and Their Heterobimetallic Complexes with Mo^0 and Pd^{II}, *Inorg. Chem.*, **2002**, *21* (10), 2070-2075.

(8) Steyn, M.; Visser, H. G.; Roodt, A.; Muller, T. J., Tetrakis(picolinato-κ^2*N,O*)zirconium(IV) dihydrate**,** Acta Cryst., 2011, E67, m1240-m1241.

(9) Pellny, P.-M.; Kirchbauer, F. G.; Burlakov, V. V.; Baumann, W.; Spannenberg, A.; Rosenthal, U., Reactivity of Permethylzirconocene and Permethyltitanocene toward Disubstituted 1,3-Butadiynes: η^4- vs η^2-Complexation or C–C Coupling with the Permethyltitanocene, *J. Am. Chem. Soc.* **1999**, *121*, 8313-8323.

(10) Dolomanov, O. V.; Bourhis, L. J.; Gildea, R. J.; Howard, J. A. K.; Puschmann, H., OLEX2: a complete structure solution, refinement and analysis program. *J. App. Cryst.* **2009,** *42* (2), 339-341.

(11) Sheldrick, G., SHELXT - Integrated space-group and crystal-structure determination. *Acta Cryst. A* **2015,** *71* (1), 3-8.

(12) Sheldrick, G., Crystal structure refinement with SHELXL. *Acta Cryst. C* **2015,** *71* (1), 3-8.
